# Strike at the root: exploring the transferability of heat stress tolerance in tomatoes by reciprocal grafting

**DOI:** 10.3389/fpls.2025.1549737

**Published:** 2025-07-24

**Authors:** Robin T. Biermann, Linh T. Bach, Christian M. Steuer, Julia J. Reimer, Dietmar Schwarz, Frederik Börnke

**Affiliations:** ^1^ Plant Adaptation, Leibniz Institute of Vegetable and Ornamental Crops (IGZ), Großbeeren, Germany; ^2^ Institute of Biochemistry and Biology, University of Potsdam, Potsdam, Germany; ^3^ Plant Quality and Food Security, Leibniz Institute of Vegetable and Ornamental Crops (IGZ), Großbeeren, Germany; ^4^ Faculty of Life Sciences and Technology, Berliner Hochschule für Technik, Berlin, Germany; ^5^ Faculty of Technology, Molecular Biosciences, University of Applied Sciences Emden/Leer, Emden, Germany

**Keywords:** Solanum lycopersicum, tomato, grafting, abiotic stress, heat stress, stress tolerance, phenotyping, transcriptomics

## Abstract

Heat stress (HS) poses a significant threat for tomato (*Solanum lycopersicum* L.) cultivation, leading to reduced yield throughout the production cycle. In addition to breeding, a promising approach to enhance HS tolerance is through grafting. For this, rootstocks obtained from tolerant genotypes are joint with susceptible scions that possess superior fruit traits. This study aims to test whether *a priori* knowledge of tolerance levels can be used to facilitate the identification of suitable grafting combinations, while simultaneously exploring molecular and physiological changes caused by grafting that further our understanding of the transferability of HS tolerance by grafting. The HS tolerance of tomato plants was evaluated using information about biomass development and flowering traits obtained for a diversity panel of 56 tomato genotypes comprising Mediterranean landraces cultivated under control (22/18°C) and HS (35/25°C) conditions. As result genotype T12 was identified with superior HS tolerance. In addition to this, a genotype with inferior HS tolerance, T48, was selected to perform reciprocal grafting experiments. Here transcriptomics data obtained from leaf tissue of grafted plants after a seven-day treatment period indicated global changes in gene expression with a special impact on components of the photosystem. Alongside, transcription factors and regulators such as ARID (Solyc01g111280.3.1), DDT (Solyc11g006200.2.1), GNAT (Solyc02g064690.3.1), and Jumonji (Solyc01g006680.4.1) were identified as potentially important targets for tolerance breeding. Long-term cultivation of grafted plants including eleven weeks of treatment supported the tolerance classification of the genotypes by the means of biomass and yield. Eventually, yield data indicated that the HS susceptible genotype (T48) lowered the yield of the usually tolerant scion (T12). Observed influences on the photosystem of the grafted plants were associated with the treatment rather than the grafting. In summary, these experiments indicated that HS tolerance or susceptibility, respectively can be conferred by grafting. However, more sophisticated screening techniques might be needed to successfully predict stress alleviation by grafting pair selection. Eventually, HS adaptation responses of the tomato plants might offer a potential for targeted breeding or engineering of tolerant genotypes, with a special focus on genes involved in epigenetic remodelling.

## 1 Background

Driven by climate change, abiotic stressors are increasingly affecting tomato production worldwide at multiple growing stages (Hazra et al., 2007; Wahid et al., 2007; Golam et al., 2012). Among them, heat stress (HS), in particular, has the potential to cause drastic yield losses (Berry and Uddin, 1988; Hazra et al., 2007; Ayankojo and Morgan, 2020). When examining the effects of stress on plant growth, it is fundamental to reflect the basic definitions of stress and growth. The term HS is defined as the occurrence of temperatures that supersede the optimal range of growing temperatures leading to adverse effects on growth, development, and cellular processes (Lichtenthaler, 1998; Wahid et al., 2007). While in general the optimal growing temperature for tomato plants ranges between 21°C to 26°C during the day and 15°C to 20°C during the night, actual optimal growing temperatures vary depending on the genotype (Hazra et al., 2007). A comprehensive review on the potential impact of HS on yield was published by Hazra et al. (2007), which summarised information on flower development-related traits, oxidative stress at the cellular level, influences on the photosystem, as well as alterations in plant hormone levels which can overall affect plant development. Out of the physiological implications of HS, pollen viability must be highlighted as a crucial aspect for fruit set under temperature stress as it directly causes implications on fruit set and yield (Peet et al., 1997, 2003; Sato et al., 2000). The impairment of pollen viability in tomato is correlated negatively with the mean daily temperature (Peet et al., 1997), while an impairment of pollen germinability and number has also been observed at increased temperatures (Sato et al., 2000). In addition to temperature, Peet et al. (2003) reported about the effects of changing vapour pressure deficits (VPD) on viability of pollen, highlighting that high humidity combined with high temperatures can result in developmental anomalies of pollen in some tomato genotypes.

Growth is fundamentally the increase in plant dry matter. Therefore, the biomass can be used to reflect the quality of overall growth conditions over a certain period (Poorter et al., 2012). Both abiotic and biotic stressors trigger changes in physiological processes which can cause deviations from optimal functioning (Golam et al., 2012; Reimer et al., 2021; Mishra et al., 2023). The concept of stress in plants, as introduced by Lichtenthaler (1998), highlights that the level and duration of a certain stress, as well as the plant’s capability to adapt and tolerate a stress are essential to discriminate between eustress and distress. The ability to adapt to fluctuating environments is crucial for plants since developmental processes such as growth and flower development are linked to and governed by abiotic factors (Monteith, 1977; Quinet and Kinet, 2007). Yet, the extent of adaptation and optimal growing conditions are determined by the genotype (Hazra et al., 2007) which allows to distinguish plants that tolerate a certain stress by their capacity to survive the stress and maintain physiological activity, growth, or yield (Thiry et al., 2016). This eventually necessitates a deeper understanding of HS tolerance and exploration of possible mitigation strategies for tomato production, as tomatoes are cultivated year-round on fields and in greenhouses (Ayenan et al., 2019; Ayankojo and Morgan, 2020).

A prominent surrogate marker that evaluates abiotic stress tolerance based on physiological changes is the measurement of the chlorophyll fluorescence and particularly the maximum quantum efficiency. Since the maximum quantum efficiency is a highly conserved chlorophyll fluorescence characteristic that reflects the efficiency of the photosystem II to capture light energy (Björkman and Demmig, 1987; Baker and Rosenqvist, 2004), deviations of its theoretical optimum indicate the presence of stress. Hence, some studies successfully utilised this measure for the selection of stress-tolerant genotypes (Yamada et al., 1996; Poudyal et al., 2019).

To assess the agronomic performance of genotypes exhibiting superior physiological stress markers, various indices were developed to quantify the tolerance or resilience further. As the concept of tolerance is relatively broadly defined, the term resilience is used to specify the maintenance of yield under conditions of stress, as outlined by Davies and Ribaut (2017). Furthermore, yield has been and still is one of the most important traits for breeding (Scott et al., 2013; Bhandari et al., 2023; Graci and Barone, 2024). Therefore, a variety of indices has been developed to estimate the impact of a stressor on the yield. For this purpose, the stress susceptibility index [SSI, (Fischer and Maurer, 1978)], the stress tolerance index [STI, (Fernandez, 1993)], the yield index [YI, (Gavuzzi et al., 1997)], the yield stability index [YSI, (Bouslama and Schapaugh, 1984)] and the relative stress index [RSI, (Fischer and Wood, 1979)] are frequently used. Each of these indices evaluate the productivity of different genotypes under control and stressful conditions, providing a quantitative measure of the goodness of tolerance and resilience, respectively, which can be incorporated into breeding programs.

Although modern tomato varieties gained increased yield and diversity in fruit-related traits throughout the breeding process (Schouten et al., 2019), traits that confer tolerance to abiotic stresses have been neglected in the past (Tanksley and McCouch, 1997; Zhang et al., 2017). This focus has inadvertently led to a reduction in genetic diversity among modern tomato varieties (Alonge et al., 2020). However, early domesticated genotypes and landraces have been identified as a reservoir for genetic diversity. At present, numerous studies investigate those traditional tomato plants for their potential application in trait discovery, marker development and breeding programs (Zhang et al., 2017; Fernie and Yan, 2019; Ruggieri et al., 2019).

Among the modern horticultural techniques used to improve plant productivity and tolerance is an ancient one: grafting (Tsaballa et al., 2021). Grafting is the process of joining two tomato plants of different genotypes. If the plants are compatible, traits such as tolerance to abiotic stress, resistance to biotic stress, or fruit flavour can be transferred and influenced from the rootstock to the scion and *vice versa* (Schwarz et al., 2010; Colla et al., 2017; Zhou et al., 2022). However, the extent and transferability of desired traits remain unpredictable (Tsaballa et al., 2021). Therefore, superior rootstocks are usually identified by screening numerous combinations of hetero grafts and comparing them with homo-grafts and non-grafted control plants under greenhouse conditions. Special rootstock varieties such as the commercially available tomato rootstocks ‘Maxifort’ or ‘Optifort’ are widely used for the cultivation of tomato plants in greenhouses with the aim of year-round production, as they have been proven to promote growth or transfer cold tolerance to scions (Mišković et al., 2009; Lee et al., 2010). Both of these varieties are characterised by a vigorous root system, which has been used as a predictor of rootstock quality (Colla et al., 2017). Recently, intra-species grafting with wild tomato species and other Solanaceae has also been a focus of research, as some studies indicated that these grafts can ameliorate diverse abiotic stresses (Schwarz et al., 2010; Colla et al., 2017; Lee et al., 2023). As rootstock and scion can exchange signals, such as hormones or small RNAs, to modulate gene expression in distant tissues (Tsutsui and Notaguchi, 2017), extensive phenotyping or targeted trait observations are carried out to unravel how novel grafting combinations affect below- and above-ground plant traits.

In this study, a diversity panel comprising tomato landraces was screened for HS tolerant and susceptible genotypes by phenotyping early developmental stages. The resulting classification was used to test whether *a priori* knowledge of tolerance levels can decrease the number of screenings needed to identify superior graft combinations and rootstocks, respectively. Hereafter, HS response and adaptation of reciprocal grafts involving two tomato genotypes of contrasting HS tolerance were investigated using transcriptomics data obtained from leaf tissue. This explorative approach was used to extend the knowledge about how rootstocks can influence HS tolerance related traits in leaf tissue of scions on the molecular level. Using this insight, targeted observations of stress-related traits, such as the influence on the photosystem, biomass, and yield, were obtained in a long-term grafting experiment. This eventually allowed to test and quantify the modulation of HS tolerance in scions and investigate the hypothesis that HS tolerant rootstocks can ameliorate the impact of long-term HS on yield.

## 2 Materials and methods

**Figure 1:**
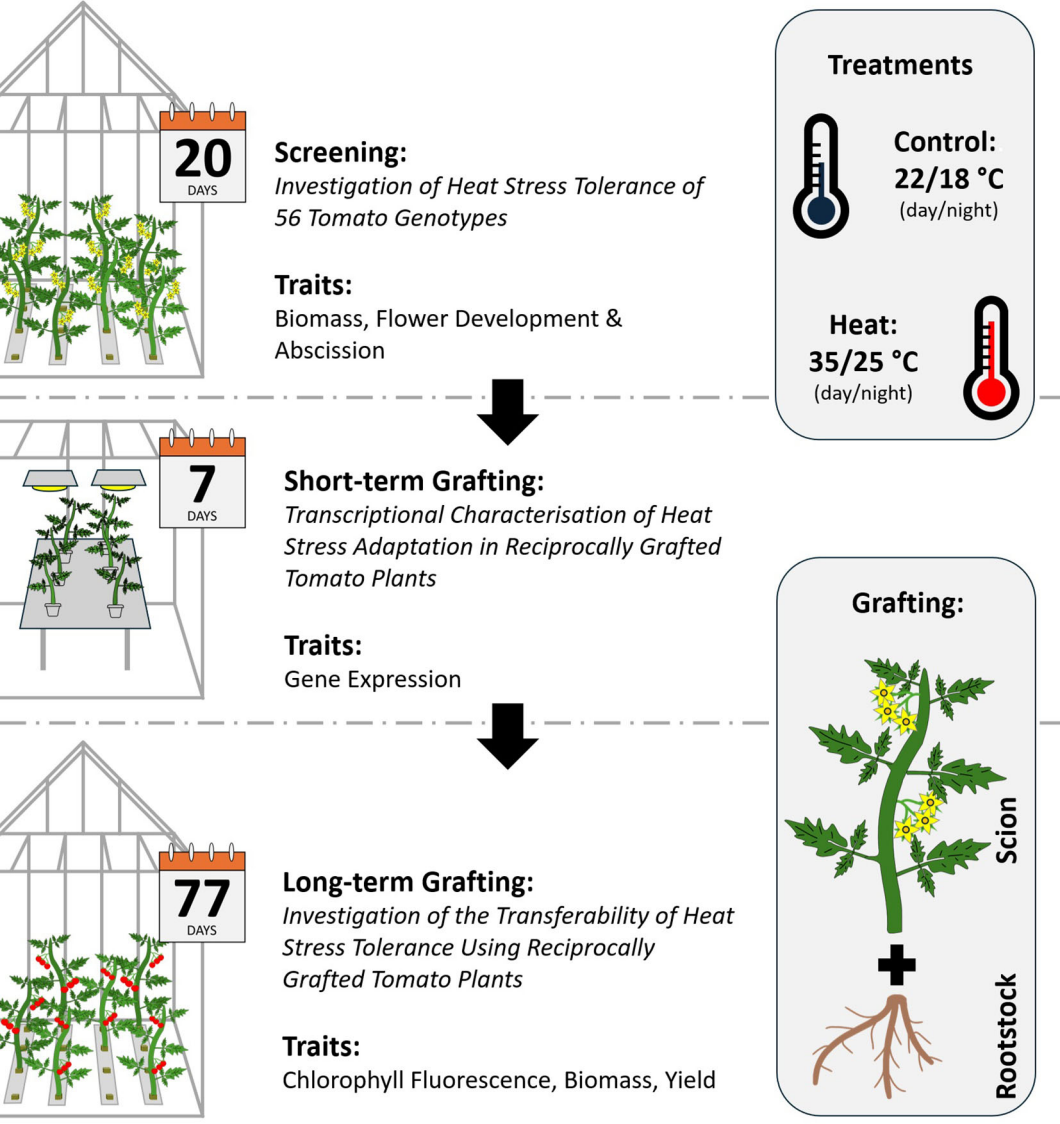
Schematic overview of the experimental design. Experiments were conducted in three consecutive steps. First, a screening of heat stress (HS) tolerance was conducted to identify tolerant and susceptible tomato genotypes amongst a diversity panel comprising Mediterranean tomato landraces. Second, a tolerant and a susceptible genotype were selected for reciprocal grafting. HS responses and adaptations were investigated with transcriptomics in leaf tissue of scions. Third, reciprocally grafted tomato plants were cultivated for targeted investigations of chlorophyll fluorescence, biomass and yield to test the transferability of HS tolerance traits.

The following section describes the methodologies applied for three lines of screening and grafting experiments with *Solanum lycopersicum* L. The first experiment aimed to identify HS tolerant and susceptible tomato genotypes amongst a panel of 56 tomato accessions. Hence, the experiment is referred to as ‘screening’. With the results of the initial screening, two genotypes of contrasting HS tolerance were selected for reciprocal grafting experiments. Here, grafted plants were again cultivated under control and HS conditions. The graft combinations are denoted as rootstock/scion (R/S). The first short-term grafting experiment is referred to as ‘transcriptomics experiment’ and was conducted to investigate transcriptional changes in leaves of young tomato plants. Simultaneously, a ‘long-term grafting’ experiment was initiated to investigate selected phenotypic traits, that were emphasized by the results of the transcriptomics experiment and evaluate the impact on yield. A brief overview of the experimental design is indicated in **Figure 1**.

### 2.1 Location

The screening and long-term grafting experiment were conducted in a Venlo greenhouse with a cabin size of 60 m^2^ and eight gutters per cabin located at 52°20’56.6”N, 13°18’35.8”E. Early plant cultivation and the transcriptomics experiment were carried out in walk-in climate chambers (l = 2.4 m, w = 3.85 m, h = 2.2 m; YORK^®^ GmbH, Mannheim, Germany), located on the perimeter of the site. In those, up to 36 metal halide lamps (MT400DL/BH, Iwasaki Electric Co., Japan) were used to mimic greenhouse-light conditions as defined in the following sections. Both, the greenhouse and the climate chamber were automatically controlled and monitored.

### 2.2 Plant material

The 56 different tomato genotypes were denoted with a “T” followed by a consecutive number (**Supplementary Table S1**). Information on the primary provider, variety, source code, material type, and fruit type can be found in **Supplementary Table S1**. In general, tomato seeds for the screening experiment were initially obtained from VEG-ADAPT partners (https://www.veg-adapt.unito.it/), except genotype T56 which was obtained from CULINARIS - Saatgut für Lebensmittel (Rosdorf, Germany). Seeds for the long-term and transcriptomics experiment conducted with reciprocally grafted tomato plants (T12 and T48) were propagated and supplied by GAUTIER Semences (Eyragues, France).

### 2.3 Plant cultivation

Tomato seeds were germinated in trays filled with coarse sand (grain of 0.5-1.0 mm, RIGK GmbH, Wiesbaden, Germany) under controlled environmental conditions (20/18°C (day/night), 60/85% rel. hum. (day/night), 300 μmol m^-2^ s^-1^ for 16 h) in walk-in climate chambers (YORK^®^ GmbH, Mannheim, Germany). The seedlings were irrigated with water and were cultivated until the formation of the first true leaves. Hereafter, the plants were either transferred into 10 x 10 x 6.5 cm rockwool cubes (Plantop, Grodan, Roermond, Netherlands) for the screening experiment or prepared for grafting by transplanting them into grafting trays (JP 3050/72 P, Pöppelmann GmbH & Co. KG, Lohne, Germany).

Splice grafting was performed as described by Singh et al. (2017). Briefly, seedlings were divided into rootstock and scion by horizontal stem cuts above the cotyledons. The cuttings were then reconnected with flexible silicon clips (Volmary GmbH, Münster, Germany) in a reciprocal manner. After this procedure, plants were regenerated at constant environmental conditions of 20°C, 95% rel. hum. and lighting of 80-90 μmol m^-2^ s^-1^ for 12 h for seven days in walk-in climate chambers (YORK^®^ GmbH, Mannheim, Germany). Afterwards, plants were transferred into 10 x 10 x 6.5 cm rockwool cubes (Plantop, Grodan, Roermond, Netherlands) for the long-term phenotyping experiment or planting pots (d = 15 cm; V = 1.3 L, Pöppelmann GmbH & Co. KG, Lohne, Germany) filled with coarse sand (grain of 0.5-1.0 mm, RIGK GmbH, Wiesbaden, Germany) for the transcriptomics experiment.

During cultivation of plants in the greenhouse, senescent leaves and side branches were pruned. The removed tissue was collected and accounted to the whole biomass. Parasitic wasps (*Encarsia formosa*, Katz Biotech AG, Baruth, Germany) were used as preventive plant protection measure in the greenhouse. Flowers were manually pollinated three times per week to ensure consistent pollination in the long-term grafting experiment. To diminish unintended experimental influences on the transcriptomics experiment, no nursing procedures were applied to young plants. All plants were regularly checked for infections by visual inspection. No infections were observed.

### 2.4 Fertigation

After transplanting, seedlings and young plants were fertigated daily with a De Kreij nutrient solution adapted for tomato (De Kreij et al., 1997). Similar to Biermann et al. (2022), the nutrient solution for young plants (electric conductivity (EC) = 1.5 dS m^-1^, pH = 5.6) was composed of following nutrients: nitrate (10.75 mmol L^-1^), ammonium (1 mmol L^-1^), potassium (6.5 mmol L^-1^), phosphate (1.25 mmol L^-1^), magnesium (1 mmol L^-1^), sulfate (1.5 mmol L^-1^), calcium (2.75 mmol L^-1^), iron (15 µmol L^-1^), manganese (10 µmol L^-1^), zinc (4 µmol L^-1^), boron (20 µmol L^-1^), copper (0.75 µmol L^-1^), molybdenum (0.5 µmol L^-1^). For fertigation of plants in the greenhouse and application of a nutrient film technique system, stock solutions were used and mixed with distilled water by a computer supported irrigation system to produce nutrient solution with an EC of 2.0 dS m^-1^. The nutrient film technique system irrigated the gutters for 30-60 s every 5 minutes with nutrient solution ensuring wetting of roots at all developmental stages. Excess nutrient solution was recirculated, replenished, and exchanged every other week as suggested by Halbert-Howard et al. (2021).

### 2.5 Treatments and experimental design

#### 2.5.1 Initial screening of tomato plants towards heat stress tolerance

For the screening of the tomato diversity panel (**Supplementary Table S1**) towards HS tolerance, seeds were sown on 03.03.2020. Upon germination and transplanting into rockwool cubes, tomato plants were cultivated at control conditions in six greenhouse cabins with a heating setpoint of 20/18°C (day/night) and ventilation setpoint of 22°C. Each of the eight gutters per greenhouse cabin contained up to three plants of seven randomly positioned genotypes. Hence a total of up to three experimental replicates per genotype and treatment were investigated. The HS treatment was initiated in three cabins on 17.04.2020. The applied treatment consisted in elevated day/night set points of 33/25°C for heating and 35°C for ventilation. After 20 days of treatment, the experiment was terminated on 06.05.2020. During the treatment period, the average temperatures and relative humidity were 21.1/18.5°C (day/night) and 54.7/68.4% (day/night) under control conditions, and 31.1/24.1°C (day/night) with 61.5/63.7% (day/night) under HS conditions. Detailed information on actual temperature, humidity, Vapor Pressure Deficit (VPD) and radiation in the greenhouse are available in **Supplementary Table S2**. Overall, plant biomass (see 2.6.1) and flowering traits (see 2.6.2) were investigated during the initial screening experiment.

#### 2.5.2 Transcriptomics experiment on young, grafted tomato plants

For the transcriptomics experiments with young, grafted plants, seeds were sown on 14.01.2022 and grafted on 26.01.2022. After seven days of regeneration, plants were grown at control conditions which were set to 20/18°C (day/night), 60/85% rel. hum. (day/night), 500 μmol m^-2^ s^-1^ for 16 h and 400 ppm CO_2_. On 22.02.2022 half of the plants were transferred into an identical walk-in climate chamber in which the plants were adapted to higher temperatures with heating settings of 29/21°C (day/night). One day later, the HS treatment was initiated, and temperatures were increased to 35/25°C (day/night). The experiment was terminated on 01.03.2022, after seven days, with the sampling of leaf tissue from the 5^th^ leaf from top of the plant for further use in transcriptomic analysis. Here, a total of four biological replicates were sampled between 9:30-10:30 a.m. and around 3.5-4.5 h after onset of the day-conditions, respectively. During the cultivation, plants were randomised and shuffled twice a week. Eventually, the transcriptomics experiment was carried out to investigate influences of heat stress on gene expression (see 2.8).

#### 2.5.3 Long-term phenotyping of grafted tomato plants

The long-term phenotyping experiment started with the sowing of the two tomato genotypes T12 and T48 on 26.01.2022. Upon successful reciprocal grafting, plants were transferred into two identical greenhouse cabins on 22.02.2022 and were cultivated in a fully randomized manner under control conditions (heating set points: 20/18°C (day/night); ventilation set point: 22°C). Each of the greenhouse cabins contained eight gutters which were used to cultivate three plants per graft each. The outmost gutters, as well as the last plant in each row, were considered as marginal planting and were neglected for data analysis. Hence, up to 18 biological replicates per treatment and graft were investigated. On 06.04.2022 the HS treatment period was initiated in one of the greenhouse cabins. For this, the heating setpoint was elevated to 33/23°C (day/night) and ventilation was initiated above 35°C. Simultaneous with the start of the treatment, a recently developed leaf was marked with a white plastic label, which served as a reference for phenotypic observations. After 11 weeks, the experiment was terminated on 23.06.2022. During the treatment period, the average temperatures and relative humidity were 21.9/17.7°C (day/night) and 76.2/83.8% (day/night) under control conditions, and 27.5/20.5°C (day/night) with 70.7/68.0% (day/night) under HS conditions. Detailed information on actual temperature, humidity, VPD, and radiation in the greenhouse are available in **Supplementary Table S2**. Throughout the long-term grafting experiment, data on growth, yield, and leaf physiological traits was collected (see 2.6).

### 2.6 Collection of phenotypical and physiological data

#### 2.6.1 Plant biomass

Plant biomass was determined by weighing the shoot fresh weight. Subsamples of plants from the greenhouse obtained within the screening and long-term grafting experiment were subsequently dried at 80°C for five days in a drying cabinet (FP 720, BINDER GmbH, Tuttlingen, Germany) to determine the dry weight. Root dry weight was determined upon five days of drying at 80°C in a drying cabinet and removal of residual sand. A precision scale (Kern^®^ PCB3500-2, KERN & SOHN GmbH, Balingen, Germany) was used to weigh in subsamples, and dried material. A platform scale (Kern^®^ 880-22, KERN & SOHN GmbH, Balingen, Germany) was used to determine the fresh weight of whole plants from the greenhouse.

#### 2.6.2 Yield, flower abscission rate, and related traits

To examine the influence of long-term HS on potential yield loss during the vegetative growth phase of tomato, the proportion of aborted to the total number of developed flowers was investigated within the screening experiment. To do so, the number of developed and dropped flowers was counted manually for the first four trusses of each plant. The counting was conducted weekly during the treatment.

As pivotal measure of stress tolerance, yield was investigated during the long-term grafting experiment. To do so, fruits of four selected trusses per plant that developed during the treatment were observed. Ripe fruits were harvested and weighed on a weekly basis. The number of tomatoes per plant was recorded. To compare differences, the relative number of fruits was normalized to the respective mean of each graft combination under control treatment conditions.

#### 2.6.3 Ion leakage

To assess the influence of long-term HS on membrane permeability in grafted plants, ion leakage was measured on 13.05.2022, the 38^th^ day of treatment, using the 5^th^ leaf from top, following an adapted protocol from Camejo et al. (2005). In brief, leaf tissue was cut and rinsed three times with distilled water to remove excess cell fluids from the cut tissue. Hereafter, the leaf tissue was placed in a 20 mL polyvial (Zinsser Analytic GmbH, Eschborn, Germany) filled with 10 mL distilled water with a subsequent rotating incubation at room temperature. After 24 h, EC of the water was determined with a conductometer (GMH 3410, Greisinger, Regenstauf, Germany) to obtain the EC_24_. The tissue was subsequently lysed by boiling at 95°C for 1 h. After cooling to room temperature, the EC_max_ was determined. Ion leakage was calculated as EC [%] = 100 x EC_24_/EC_max_.

#### 2.6.4 Chlorophyll content

For non-invasive quantification of chlorophyll content, the marked leaf of the grafted plants subjected to long-term HS, were investigated with a Dualex Scientific DX18099 (FORCE-A, Orsay, France). Measurements were taken on 10.06.2022, the 66^th^ day of treatment. Up to 5 technical replicates were measured for the adaxial side of the leaves.

#### 2.6.5 Chlorophyll fluorescence

To investigate the influence of long-term HS on the plant’s photosystem, the maximum quantum efficiency (F_v_/F_m_) and non-photochemical quenching (NPQ) were measured with two LI-6800 (LI-COR Biosciences, Lincoln, Nebraska, USA) systems on 11.05.2022, the 36^th^ day of treatment. Here, a leaflet of the marked leaves from the long-term grafting experiment was investigated. Fluorescence characteristics were detected after dark adaptation with a lightproof Dark Adapting Clip Kit (LI-COR Biosciences, Lincoln, Nebraska, USA) for 20 min and subsequent exposure to a light flux of 1000 μmol m^-2^ s^-1^. The values of F_0_ and F_m_ were measured on dark adapted leaves, and F_s_, F_m’_ and F_0’_ on light adapted leaves using the rectangular measuring mode. The instruments chamber settings and fluorescence measuring mode setting were set as listed in **Supplementary Table S3**.

### 2.7 Statistical evaluation of phenotypic and physiological data

Before testing for significant changes, data was checked for normal distribution and homoscedasticity. Following visual inspection of normal distribution of residuals a Shapiro-Wilk’s test was performed. In case data deviated from normal distribution, normalisation and standardisation was performed with bestNormalize (Peterson, 2021). Variance homogeneity was tested with a Levene’s and a Fligner-Killeen’s test. Outliers were removed according to the 1.5 interquartile range criterion, if necessary. Two-sided t-tests or Wilcoxon rank sum tests were used to compare significant changes for individual genotypes of the screening experiment. Two-way-ANOVAs followed by Tukey’s *Post-hoc* tests, or Scheirer-Ray-Hare tests with subsequent pairwise Wilcoxon Rank Sum tests, respectively were calculated for the identification of homogeneous groups were used for phenotyping data of the long-term grafting experiment. If necessary, White-correction of the ANOVA model was performed to account for heteroscedasticity (Cribari-Neto, 2004). Due to experimental constraints, the main effect of temperature might be confounded with cabin-specific factors and should be interpreted with caution. However, the reciprocal grafting design ensures that graft effects and interactions with temperature should remain valid within the experimental setup.

Yield data was evaluated based on selected stress indices, that were included in the iPASTIC tool (Pour-Aboughadareh et al., 2019). The stress indices applied in this study were: SSI (Fischer and Maurer, 1978), STI (Fernandez, 1993), YI (Gavuzzi et al., 1997), YSI (Bouslama and Schapaugh, 1984), and RSI (Fischer and Wood, 1979).

### 2.8 Bioinformatics and gene expression studies

#### 2.8.1 RNA extraction, quality control, and sequencing strategy

For bioinformatics analysis of gene expression, leaf tissue sampled from young tomato plants at the end of the transcriptomics experiment was used. After sampling, leaf tissue was flash-frozen in liquid nitrogen and stored at -80°C until further use.

Up to 100 mg of plant material were homogenized with a bead mill (MM400, Retsch GmbH, Haan, Germany) using 5 mm steel beads. Frozen samples were homogenized at a frequency of 30 Hz for 45 s. Afterwards leaf material was transferred into pre-cooled 2 mL reaction tubes (SARSTEDT AG& Co., Nümbrecht, Germany) for subsequent total RNA extraction. For this, QIAGEN Plant RNeasy Mini Kits (QIAGEN GmbH, Hilden, Germany) were used, following the manufacturer’s protocol.

An initial quantification of the total RNA was done with an Implen NanoPhotometer^®^ (Implen GmbH, München, Germany). Hereafter, RNA integrity numbers were investigated. For this, 2 µL of total RNA was analysed with an Agilent 2100 Bioanalyzer (Agilent Technologies, Waldbronn, Germany) and the Agilent RNA 6000 Nano Kit (Agilent Technologies, Waldbronn, Germany), following the manufacturers protocol and the settings for “Plant RNA Nano”.

After RNA quality control, total RNA was shipped to Novogene (United Kingdom) for sequencing. Here, RNA libraries were prepared using NEBNext^®^ Ultra RNA Library Prep Kit for Illumina (New England Biolabs, Inc., Ipswich, MA, USA) and subsequently sequenced with an Illumina NovaSeq 6000 (Illumina, Inc., San Diego, CA, USA). To summarize the process of library construction briefly, the first step was a mRNA purification with poly-T oligo-attached magnetic beads. Hereafter, mRNA was fragmented. Which was followed by synthesis of the first cDNA strand using random hexamer primers, second strand cDNA synthesis, end repair, A-tailing, adapter ligation, size selection, amplification, and purification. Eventually, libraries were sequenced on an Illumina NovaSeq 6000 S4 flow cell (Illumina, Inc., San Diego, CA, USA) as 150-nt paired-end reads, yielding an average of 25.5 M paired-end reads per sample. Descriptive sequencing statistics can be found in **Supplementary Table S4**.

#### 2.8.2 Processing and evaluation of RNA-seq data

For an initial quality control, RNA-Seq raw data was checked with FastQC (v0.11.9; Andrews, 2010). Hereafter, adapter contaminations and reads of inferior quality were trimmed with trimmomatic (v0.39; Bolger et al., 2014a) using the following settings: ILLUMINACLIP:2:30:5; MAXINFO:40:0.4; MINLEN:36. Hisat2 (Kim et al., 2019) was used to align the reads to the latest tomato reference genome (SL4.0; Hosmani et al., 2019). Mapping of the aligned reads was done with SAMtools (Li et al., 2009) using the ITAG4.1 annotation (Hosmani et al., 2019). Detailed mapping statistics can be found in **Supplementary Table S4**. Stringtie and prepDE.py (Pertea et al., 2015) were used to obtain gene count data. Finally, DESeq2 (Love et al., 2014) was used to identify differentially expressed genes (DEGs) for each graft. After variance stabilized transformation, gene expression within the biological replicates was analysed using a PCA and a correlation plot, identifying two outliers (**Supplementary Figures S1**, **S2**). After removal of outliers, genes with a false discovery rate (FDR) < 0.001 and a log2 fold change |log2FC| > 2 were considered as differentially expressed genes (DEGs).

Gene Ontology (GO) enrichment analysis was performed with the R package clusterProfiler (Yu et al., 2012; Wu et al., 2021). Significantly enriched GO terms were identified with regard to the classes: molecular function (MF), biological process (BP) and cellular component (CC). Obtained *p*-values were transformed to FDRs using the Benjamini-Hochberg method, and a threshold of *p* < 0.05 was applied. GO Annotations for SL4.0 and ITAG4.1, respectively, were identified with GOMAP (Wimalanathan and Lawrence-Dill, 2021) and were provided by the Lawrence-Dill Group (Lawrence-Dill, 2023).

### 2.9 Software tools

Additionally, to the mentioned software, R (R Core Team, 2022) and RStudio (RStudio Team, 2020) were used for data evaluation and visualization. The utilised packages were: bestNormalize (Peterson, 2021), car (Fox and Weisberg, 2019), clusterProfiler (Yu et al., 2012), ComplexHeatmap (Gu, 2022), DESeq2 (Love et al., 2014), dplyr (Wickham et al., 2020), ggbeeswarm (Clarke et al., 2017), ggplot2 (Wickham, 2016), ggpubr (Kassambara, 2018), moments (Komsta and Novomestky, 2015), multcompView (Graves et al., 2019), rcompanion (Mangiafico and Mangiafico, 2017), rstatix (Kassambara, 2023), and see (Lüdecke et al., 2021) as well as their dependencies.

## 3 Results

### 3.1 Screening for tolerant and susceptible tomato genotypes

**Figure 2:**
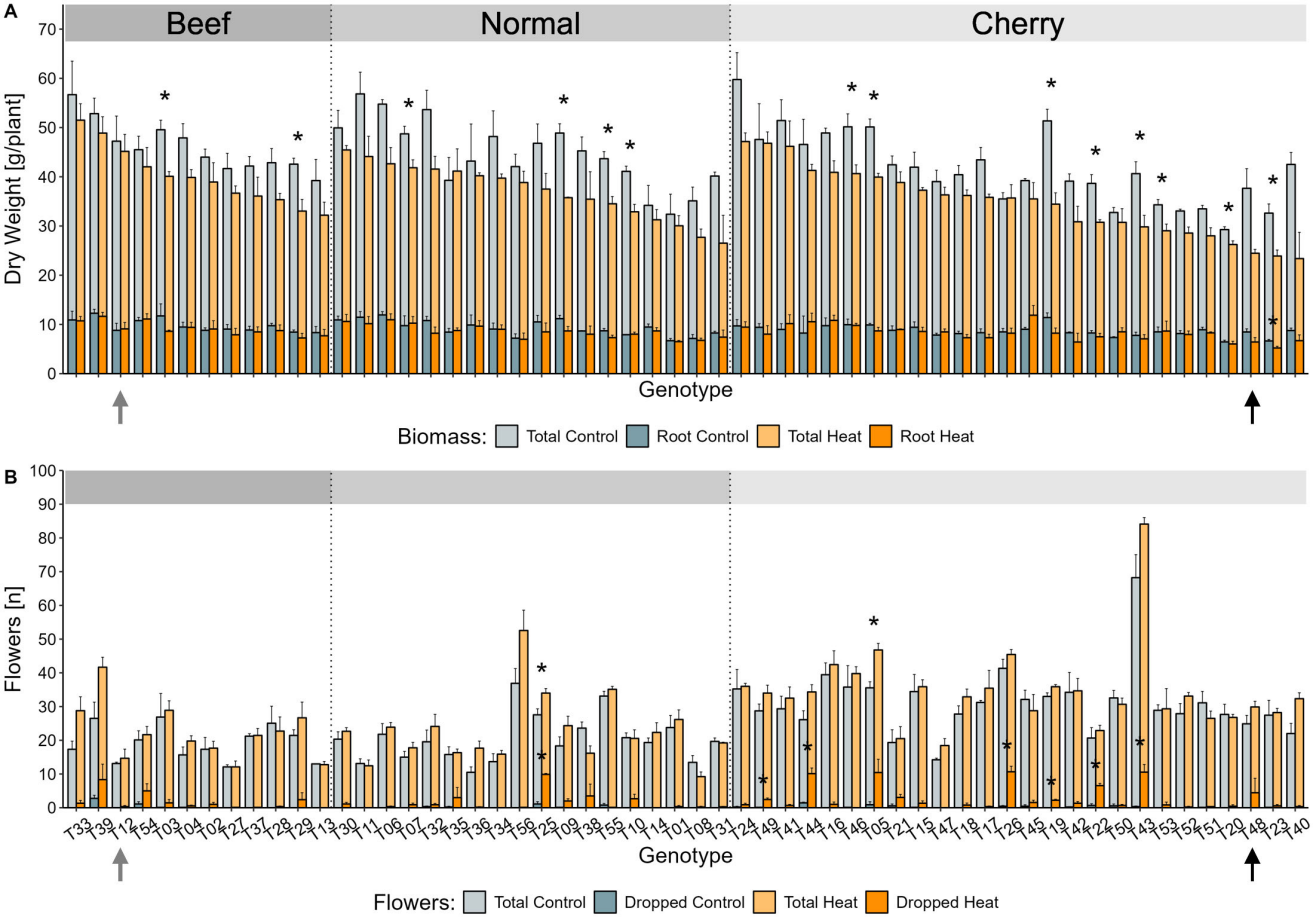
Selection of heat stress tolerant and susceptible genotypes using phenotypical screening data. Candidate genotypes for reciprocal grafting, T12 (grey arrow) and T48 (black arrow) were selected based on the combination of relative changes in biomass and flower traits. **(A)** The total plant biomass was calculated as sum of the mean shoot and root dry weight per genotype and greenhouse cabin. The proportion of the root dry weight is indicated in bright colours. Plant biomass is presented as mean ± SE for up to three experimental replicates [exception: T40 control (n = 2)]. **(B)** The number of developed (pale colours) and dropped (bright colours) flowers were manually counted for the first four developed trusses of individual plants, averaged per greenhouse cabin, and calculated as mean ± SE for up to three experimental replicates [exception: T40 control, T01 heat, T38 heat, T51 heat, T53 heat (n = 2)]. Significant differences between the treatments were tested per genotype with a two-sided t-test for the biomass traits and the number of developed flowers. Significant differences for dropped flowers were evaluated with a two-sided Wilcoxon rank sum test. Asterisks indicate a p < 0.05 between treatments.

To investigate the transferability of HS tolerance by grafting, it was essential to identify suitable genotypes of contrasting tolerance. To do so, a screening experiment was carried out with a diversity panel of 56 tomato genotypes mainly comprising landraces and commercial references (**Supplementary Table S1**; **Dataset S1**). Over the course of the trial, tomato plants developed an average number of approximately 3.7 (control) and 4.2 (heat) trusses (**Supplementary Table S5**) and rarely reached the BBCH principal growth stage 7 (development of fruits). Hence, three phenotypic traits: total plant biomass, root biomass, and the flower abscission rate (**Figure 2**), were considered relevant for an evaluation of relative plant performance and led to the selection of the putatively HS tolerant beef tomato genotype T12 and the putatively susceptible cherry tomato genotype T48. The individual results leading to this selection are discerned hereafter, with a special focus on the two genotypes.

As an indicator for the ability to maintain growth under long-term HS conditions, a key objective of the screening was to evaluate the plant biomass accumulation and maintenance under stress. Therefore, relative changes between treatments were considered and overweighted in the selection of suitable genotypes of contrasting HS tolerance, emphasizing genotypes with stable biomass (tolerant) and unstable biomass (susceptible).

Overall, prolonged HS caused a significant reduction of 16% in total biomass which was manifested in an average dry weight of around 43.5 g under control conditions, and 36.5 g under HS conditions (**Figure 2A**). Within the diversity panel, the putatively tolerant genotype T12 was observed with a slightly above average biomass accumulation under control treatment (47.2 g), which was maintained at a similar level when grown under HS conditions (45.2 g) (**Figure 2A**). The putatively susceptible tomato genotype T48 on the other hand, did only accumulate subpar amounts of biomass under both temperature conditions (control: 37.7 g, heat: 24.5 g), which displayed a tendency towards a reduction in biomass of approximately 35% under HS conditions (**Figure 2A**).

In addition to a sustained total biomass, a stable root-to-shoot ratio alongside a high amount of root biomass was considered advantageous for the selection of a tolerant rootstock. Overall, HS caused a significant reduction of the average root biomass, which was observed with 9.1 g under control and 8.6 g under HS conditions (**Figure 2A**). Those numbers reflect that the proportional contribution of roots to overall biomass were approximately 20.8% (control) and 23.5% (heat). The susceptible genotype T48 displayed a pronounced reduction of root biomass from 8.5 g to 6.4 g under HS conditions. Contrary to the global trend, the putatively tolerant genotype T12 maintained a stable root biomass of 8.8 g (control) and 9.1 g (heat) under both temperature conditions.

The second key objective of the screening was to obtain information on the potential implications of the prolonged HS treatment on yield potential. Here, the relative difference in the flower abscission rate as analysed as a predictor for possible yield losses in young tomato plants and was considered in the selection of genotypes with less weight than the biomass. In line with the increased number of developed trusses (**Supplementary Table S5**) as mentioned earlier, the average number of developed flowers increased from 25.1 (control) to 28.2 (heat) in a non-significant manner (**Figure 2B**). This observation corresponded to an average number of about 6.8 (control) and 6.7 (heat) flowers per truss, without taking the fruit types into account.

The majority of observed tomato plants did not suffer from dropped flowers under control conditions (**Figure 2B**). However, up to 30% flower loss was observed in some genotypes, particularly T22, T44, and T25, upon the exposure to prolonged HS (**Figure 2B**). The putatively tolerant beef tomato T12 did not drop any flower under control conditions and only as little as 2.3% upon HS treatment. On the other hand, the putatively susceptible cherry tomato T48 dropped an average of 4.4 flowers per plant during the HS treatment, reflecting a future yield loss of at least 14.9%.

In summary, the screening of biomass and flowering traits of the tomato diversity panel resulted in the identification of tolerant and susceptible genotypes. The beef tomato genotype T12 demonstrated remarkable resilience to long-term HS, maintaining favourable biomass and flowering characteristics, along with a robust root system. Conversely, the cherry tomato genotype T48 exhibited significantly reduced biomass and flower drop, indicating a high potential for yield loss under HS. Therefore, T48 was selected as the most heat-susceptible genotype within the diversity panel.

### 3.2 Gene expression in leaves of young, grafted tomato plants

**Figure 3:**
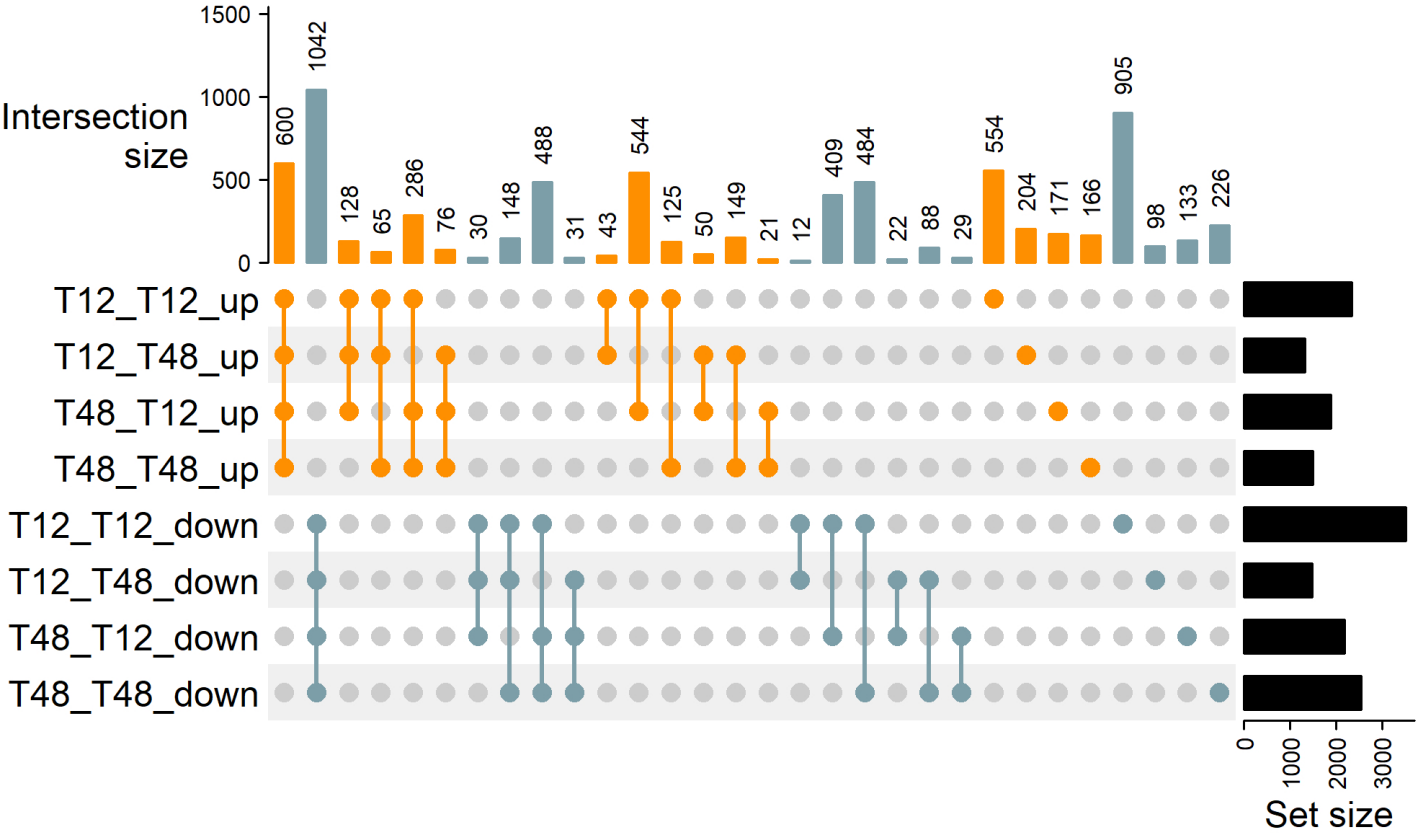
Observed differentially expressed genes are influenced by grafting combination. Upon determination of differentially expressed genes by RNA-Seq (FDR < 0.001 and |log2FC| > 2) distinct DEGs were grouped using an UpSet plot. The sets of genes identified within a graft are denoted as Rootstock_Scion_Expression on the left side of the plot. The panel Intersection size on top of the graph indicates the number of genes that are commonly induced or repressed within the selected set of grafting combinations indicated with connected dots below. The set size indicates the total number of DEGs that were identified and investigated for each graft.

**Figure 4:**
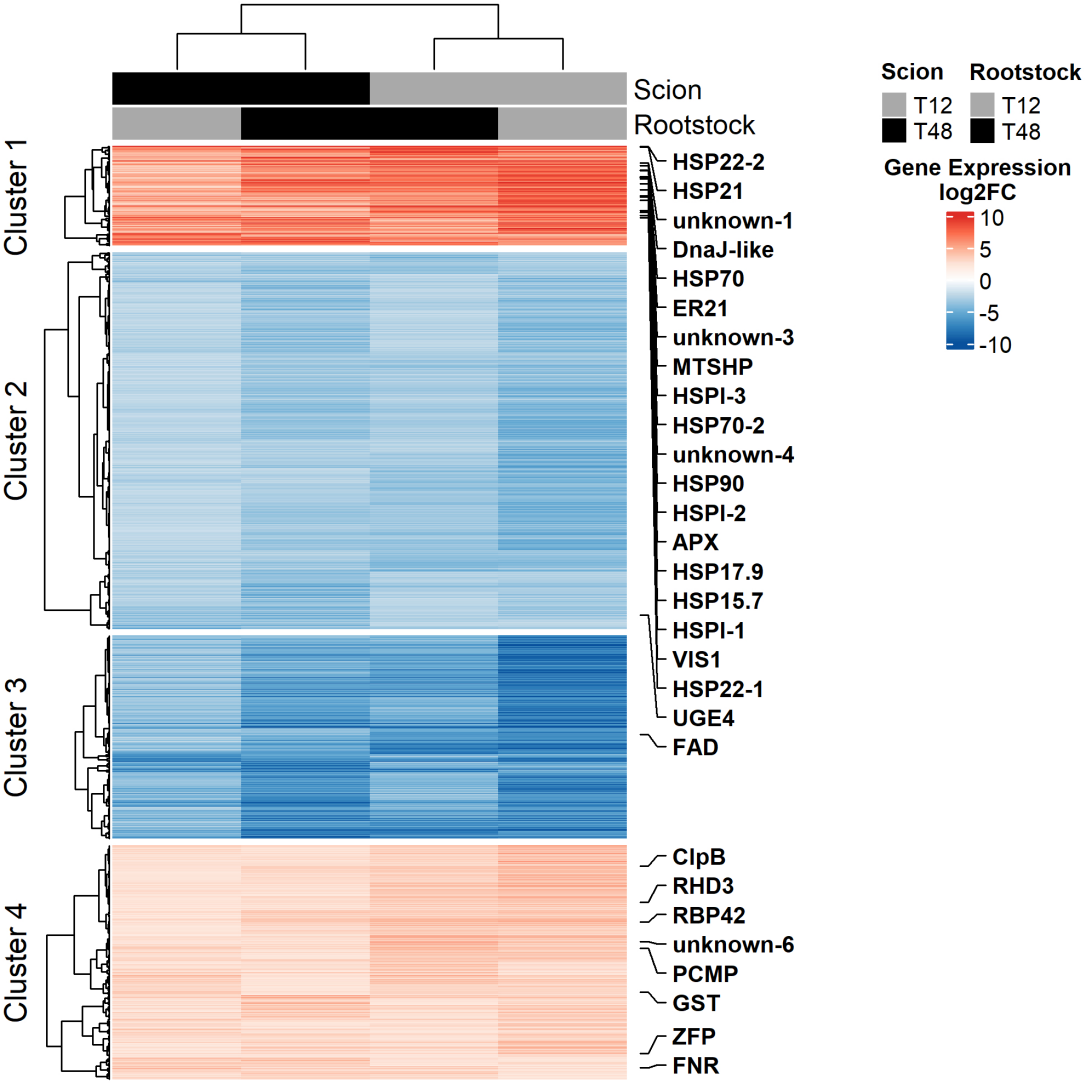
Gene expression in leaf tissue of grafted tomato plants is governed by the scion. Heatmap visualisation of log2 fold change (log2FC) transformed gene expression data. Gene expression data was obtained from leaf tissue of young, grafted tomato plants upon seven days of HS and control treatment under controlled environmental conditions. A total of 1642 DEGs (600 ↑, 1042 ↓; FDR < 0.001 and |log2FC| > 2) were clustered using Pearson distance and Ward D2. K-means clustering (k = 4) was applied to distinguish potential sub-patterns. Here, the transcripts were mapped in rows and mean gene expression in columns. Genes identified as HS core response in tomato in a meta-analysis by Psaroudakis et al. (2024) were highlighted.

To investigate the transferability of HS tolerance traits using conventional horticultural approaches, the two genotypes were used for reciprocal grafting experiments and RNA-Seq, respectively. This approach provided information on core transcriptional responses as well as changes that might have occurred due to the particular combination of tolerant and susceptible tomato plants by grafting. Applying strict criteria for DEG identification (FDR < 0.001 and |Log2FC| > 2) a total of 1642 DEGs (600 ↑, 1042 ↓) have been identified as a core response in all four grafts (**Figures 3**, **4**). While the individual number of DEGs per graft ranged between 2800 to 5879 DEGs (**Figure 3**; **Supplementary Table S6**). Not only did the DEGs contain key heat stress markers, but they also pointed to the possibility of an influence of the grafting process on the photosystem, as will be described hereafter.

To test the hypothesis, that rootstocks can transfer heat-stress-tolerance traits, intersecting DEGs found in leaf tissue of grafts with the same rootstock were investigated (**Figure 3**). The number of DEGs shared by grafts with a T12 rootstock was 55 (43 ↑, 12 ↓). GO enrichment analysis of terms belonging to biological processes (BP), molecular functions (MF) and cellular components (CC) revealed that within the set of down-regulated genes, the genes associated with the GO terms “mitotic M phase” (BP), “phenylpropanoid biosynthetic process” (BP), and “response to salicylic acid” (BP) were the top three enriched categories (**Supplementary Figure S3**). On the side of up-regulated genes, the top enriched GO terms were: “xyloglucan:xyloglucosyl transferase activity” (MF), “trans-zeatin O-beta-D-glycosyltransferase activity” (MF), and “single-stranded DNA binding” (MF).

Grafts with a T48 rootstock shared a total number of 50 DEGs (21 ↑, 29 ↓). Among the up-regulated genes, GO terms associated with “serine-type endopeptidase inhibitor activity” (MF), “plastid small ribosomal subunit” (CC), and “nucleolar ribonuclease P complex” (CC) were enriched (**Supplementary Figure S4**). Interestingly, the down-regulated genes were enriched in the terms “photosystem I” (CC), “chlorophyll binding” (MF), and “electron transport chain” (BP), highlighting a possible influence of the rootstock on the photosystem of the scion.

Subsequently, the transcriptional core adaptation response to HS common to all grafts was analysed in more detail. Hierarchical clustering of gene expression data revealed that the overall gene expression patterns observed in leaf tissue have been mainly influenced by the scion (**Figure 4**). Which is supported by the result of a principal component analysis (PCA) performed on unfiltered gene expression data (**Supplementary Figure S2**).

**Figure 5:**
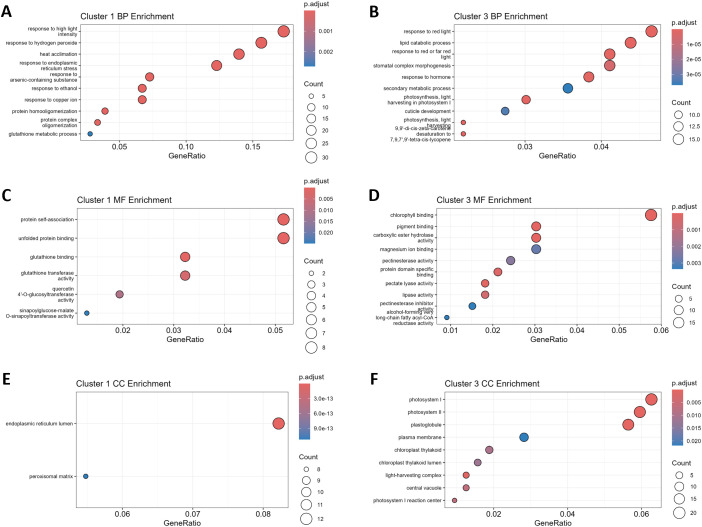
Gene ontology (GO) enrichment reveals potentially conserved heat stress adaptation responses. The panels represent significantly over-represented GO terms associated with biological processes (BP), molecular functions (MF), and cellular components (CC) for clusters 1 and 3 identified as core response upon clustering of gene expression data (**Figure 4**). **(A, B)** BP enrichment for cluster 1 and 3, respectively. **(C, D)** MF enrichment for cluster 1 and 3, respectively. **(E, F)** CC enrichment for cluster 1 and 3, respectively. The size of the circles indicates the number of genes associated with each GO term, while the colour gradient reflects the adjusted p-value (p.adjust) of the enrichment analysis.

K-means clustering (k = 4) was applied to distinguish potential sub-patterns within up- and down-regulated genes, enabling stratification into finer-grained expression trends. Out of these, cluster 1 represents the 179 most affected DEGs of the 600 up-regulated genes. GO enrichment analysis revealed, that GO terms related to the biological processes “response to high light intensity”, “response to hydrogen peroxide”, “heat acclimation”, and “response to endoplasmic reticulum stress” were the most enriched terms (**Figure 5A**). In line with that, the most enriched molecular functions were “protein self-association”, “unfolded protein binding” as well as “glutathione binding” and “glutathione transferase activity” (**Figure 5C**). On the level of the cellular compartments, the endoplasmic reticulum lumen and the peroxisomal matrix were identified as enriched within Cluster 1 (**Figure 5E**).

On the side of the down-regulated genes, Cluster 3 represented the 365 most affected genes out of the 1042 down-regulated DEGs. The most affected biological processes observed belonged to the GO terms “response to red light”, “lipid catabolic process”, “stomatal complex morphogenesis”, and “response to hormone” (**Figure 5B**). On the level of molecular functions, “chlorophyll binding”, “pigment binding”, “carboxylic ester hydrolase activity”, and “magnesium ion binding” were enriched in the gene set of Cluster 3 (**Figure 5D**). Together with the previous results, enrichment of GO terms related to the cellular compartments “photosystem I”, “photosystem II”, and “plastoglobule” highlighted that the photosystem might have suffered from considerable influences of HS, which made it a target for subsequent phenotypical studies (**Figure 5F**).

Within the complete set of 1642 DEGs, a total number of 86 transcription factors (TFs) and transcription regulators (TRs) have been identified as DEGs (16 ↑, 70 ↓; **Supplementary Table S6**). The group containing the down-regulated TFs and TRs was enriched in GO terms belonging to the biological processes: “cell differentiation”, “leaf morphogenesis”, “regulation of protein metabolic process”, “regulation of translation”, and “response to red light”. However, no significant enrichment of GO terms was observed for the up-regulated TFs (**Supplementary Figure S5**).

Manual inspection of the TF and TR classes revealed that the majority of the differentially expressed TFs and TRs are involved in the regulation of processes that belong to the categories: “stress response”, “ROS scavenging and oxidative stress”, “photosynthesis and biomass accumulation”, “development and differentiation”, “chromatin remodelling and regulation of transcription”, as well as “gene expression regulation and signalling” (**Supplementary Table S6**). The mode of action of the detected TFs aligns well with the enriched GO terms observed in the transcriptomes. Among the differentially expressed TFs, a heat shock transcription factor (Solyc08g062960.5.1), which is a master regulator of HS response was identified with a high abundance under HS conditions. Transcripts of a cold shock protein (Solyc01g111280.3.1), on the other hand were less abundant under HS conditions. Further classes of TFs that play a role in the heat stress response were observed. Several transcripts of TFs belonging to the basic-leucine zipper (BZIP) and MYB families were found to be less abundant under heat stress conditions. Some transcripts of WRKY family TFs were also observed to be less abundant, while others were found to be more abundant in leaf tissue after treatment. Interestingly, TRs of the classes ARID (AT-rich interacting domain-containing proteins; Solyc01g111280.3.1), DDT (DDT domain-containing proteins, Solyc11g006200.2.1), GNAT (Gcn5-related N-acetyltransferases, Solyc02g064690.3.1), and Jumonji (Jumonji family proteins, Solyc01g006680.4.1), which are responsible for modification of chromatin, have been identified as well.

In summary, leaf transcriptomics revealed that the prolonged HS treatment has been the primary factor influencing gene expression in leaf tissue of grafted tomato plants. The observed core adaptation response was resembled by DEGs that encode a set of well-known HS markers such as HSPs, antioxidative enzymes and related TFs and TRs. Furthermore, gene expression was clearly influenced by the investigated scions, and the grafting itself merely affected gene expression. Nevertheless, the limited number of DEGs that were identified with matching expression patterns in leaf tissue of grafts sharing the same rootstock in combination with distinct scions suggested the possibility of an influence on the photosystem caused by grafting.

### 3.3 Effects of grafting on physiological and agronomical traits in a long-term experiment

**Figure 6:**
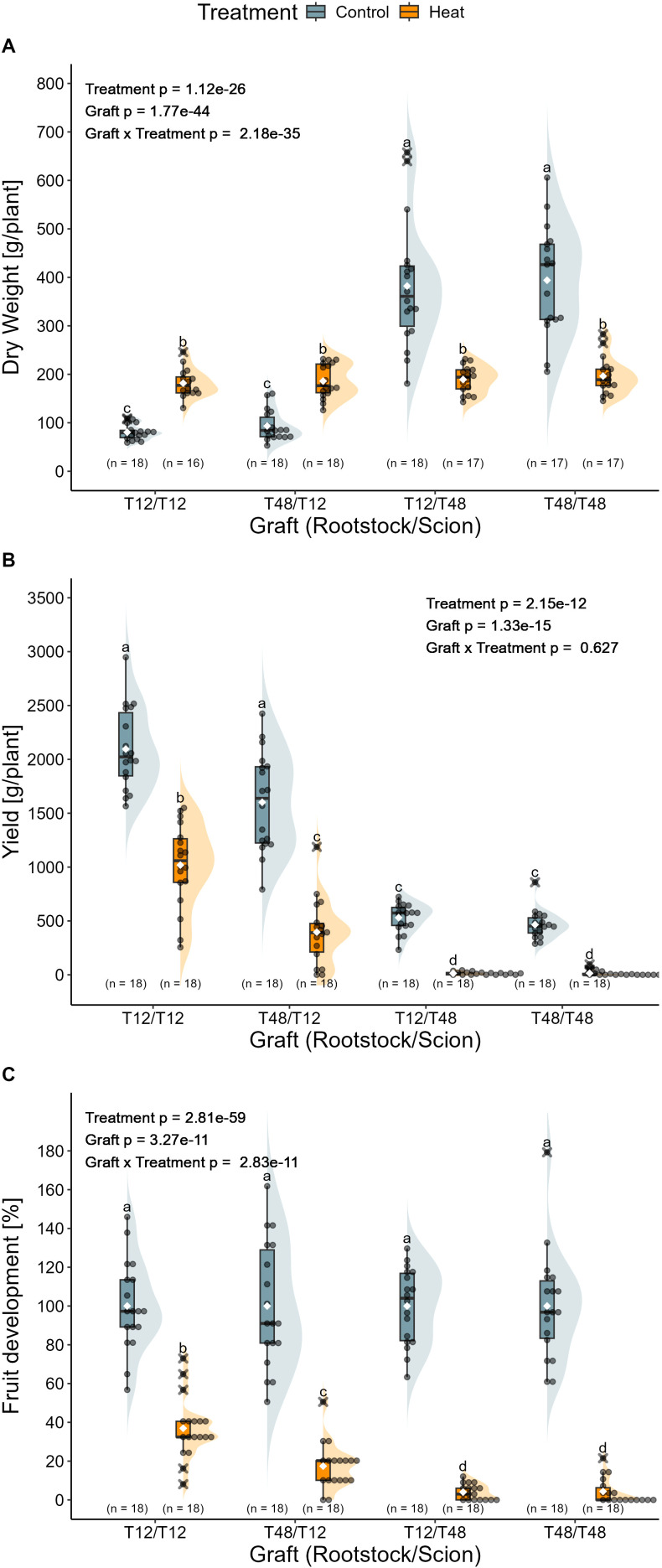
Impact of long-term heat stress on the biomass, yield and fruit development of grafted tomato plants. **(A)** Total biomass presented as dry weight, **(B)** total yield of four trusses, and **(C)** relative fruit development normalised to the control treatment are shown for the different graft combinations. Grafted plants were cultivated in a greenhouse and exposed to the treatment conditions for 11 weeks. Statistical significance of biomass and fruit development was tested using normalized data. Statistical analysis was performed with a two-way ANOVA followed by Tukey’s HSD *post-hoc* test. The ANOVA model for biomass included a White-correction. For the analysis of yield, a Scheirer-Ray-Hare test with subsequent pairwise Wilcoxon rank sum tests was used. Significant differences are indicated as letters for the interaction Graft x Treatment (p < 0.05), while the level of significance for the factors is indicated as text element in the graph. The number of biological replicates after outlier removal is noted above the x-axis.

After the transcriptomics experiment on young plants, a long-term experiment with reciprocally grafted tomato plants was carried out in the greenhouse which included a treatment duration of 11 weeks. The aim of the long-term grafting experiment was to validate the screening methods and examine the impact of grafting on agronomically and physiologically relevant traits. In order to test the suitability of the selection method used for screening of young tomato plants during vegetative growth, the influence of HS on the biomass of reciprocally grafted plants is focused on first. Overall, significant influences were detected for treatment, graft, and for the interaction of both factors. Observations of the self-grafted tomato plants T12 and T48 resembled the findings from the screening experiment. T12/T12 again displayed an increased biomass from 80 g/plant to 182 g/plant after HS treatment. While T48/T48 showed a biomass reduction of 50%, resulting in the observation of 394 g/plant under control conditions and 196 g/plant under HS conditions (**Figure 6A**). Regardless the rootstock, shoot biomass accumulation seemed to be mainly influenced by the scion, as T48/T12 and T12/T48 followed the trends observed for the self-grafted T12/T12 and T48/T48, respectively. After the 11 weeks of HS treatment, there was almost no difference between the biomass of T12/T48 (382 g/plant (control) and 189 g/plant (heat)) and the self-grafted T48/T48. Similarly, the combination T48/T12, displayed no significant increase in biomass when compared to the self-grafted T12, leading to the measurement of 93 g/plant under control and 186 g/plant under HS treatment.

To confirm the different HS tolerances between T12 and T48 tomato plants, and test the hypothesis, that grafting can influence HS resilience, yield was monitored for four selected trusses and resulted in the observation of significant effects of the treatment and the graft. Overall, scions of the tolerant genotype T12 produced the highest yield under both temperature regimes. The best performing graft combination was the self-grafted T12, reaching an average yield of 2096 g under control conditions and 1018 g under HS conditions (**Figure 6B**). When T48 served as rootstock for a T12 scion, yield was significantly reduced to an average of 1602 g (control) and 396 g (heat). Regardless of the rootstock, T48 scions did produce roughly similar yields of around 531 g (T12/T48) and 465 g (T48/T48) under control conditions. HS caused a significant reduction to as little as 14 g in both grafts. In addition to that, the number of plants that developed any fruit was reduced by up to 56% (n = 8) for the self-grafted T48, supporting the classification of T12 as HS tolerant and T48 as HS susceptible genotypes.

To quantify the impact of the HS treatment on the plants, a set of yield-based stress stability indices were calculated (**Table 1**). The resulting numbers for the Stress Susceptibility Index (SSI), Stress Tolerance Index (STI), Yield Index (YI), Yield Stability Index (YSI), and Relative Stress Index (RSI), supported the observation, that the self-grafted T12 had superior HS tolerance over all other graft combinations and T48, respectively. Additionally, the overall better performance of T12 scions was highlighted by the results again, as T48/T12 was indicated as the second-best performing graft.

**Table 1:** Assessment of yield stability produced by grafted tomato plants under heat stress (HS) conditions indicated transferability of susceptibility.

Graft (rootstock/scion)	Yield control (g)	Yield treatment (g)	SSI	STI	YI	YSI	RSI
T12/T12	2096	1018	0.74	1.55	2.82	0.49	1.58
T48/T12	1602	396	1.09	0.46	1.10	0.25	0.80
T12/T48	531	14	1.41	0.01	0.04	0.03	0.08
T48/T48	466	14	1.40	0.00	0.04	0.03	0.10

The table shows the average yield obtained from four trusses of 18 plants per graft and treatment (**Figure 6B**). Alongside derived yield-based stress stability indices: SSI (Stress Susceptibility Index), STI (Stress Tolerance Index), YI (Yield Index), YSI (Yield Stability Index), and RSI (Relative Stress Index) are provided.

The relative number of fruits per plant, normalised to the control treatment, aligned with yield observations (**Figure 6C**). Overall, significant influences were observed for the treatment, the grafts, and for the interaction. Plants with T48 scions produced only about 4% of the fruit observed under control conditions, whereas T12 self-grafts reached approximately 37%. The combination of T48 rootstocks and T12 scions resulted in a reduced fruit count, representing 17% of the control performance.

**Figure 7:**
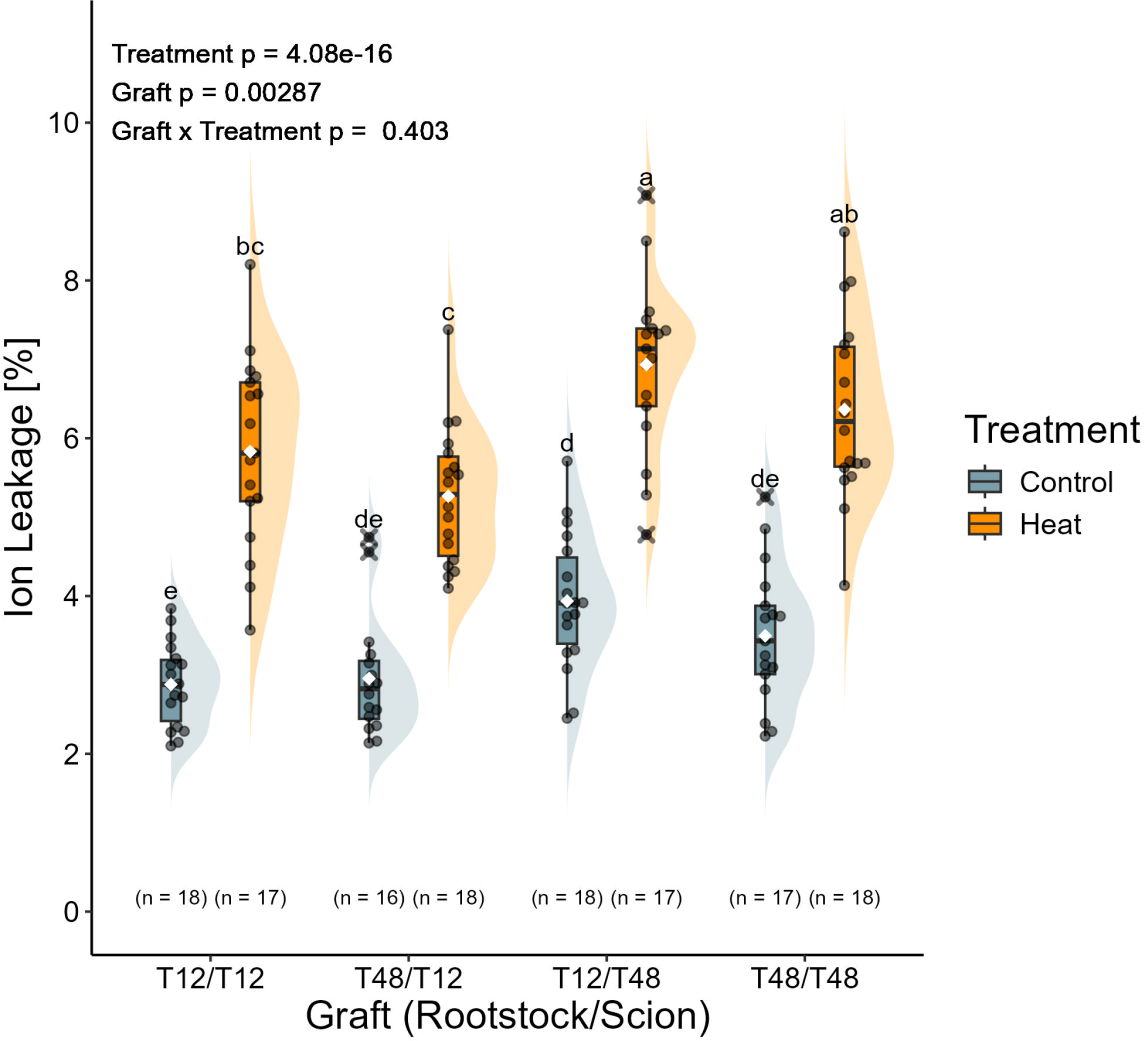
Heat stress increased membrane permeability regardless of the graft. Membrane permeability was assessed with tissue of the 5^th^ leaf from top, which developed during the treatment. Sample collection was carried out after 37 days of treatment. Grafted plants were cultivated in a greenhouse environment. A two-way ANOVA followed by Tukey’s HSD *post-hoc* test was used to analyse statistical significance. Significant differences are indicated by different letters for the interaction Graft x Treatment (p < 0.05), while the level of significance for the factors is indicated as text element in the graph. The number of biological replicates after outlier removal is noted above the x-axis.

Leaf ion leakage indicated a significant influence for the treatment and the grafts, but not for the interaction. The leaves of all plants grown under control conditions were observed with an average ion leakage of around 3.3%, which barely differed significantly among the grafts. The average ion leakage measured for leaves upon long-term HS exposure was at around 6.1%. Among the significantly affected plants, the graft combination T12/T48 displayed the highest measured ion leakage of around 6.9% under HS conditions, while the other reciprocal graft T48/T12 did only reach a level of 5.3%, which was significantly lower than the former (**Figure 7**). However, the ion leakage of both reciprocal grafts did only deviate from the self-grafted T12 (5.8%) and T48 (6.4%) in a non-significant manner.

**Figure 8:**
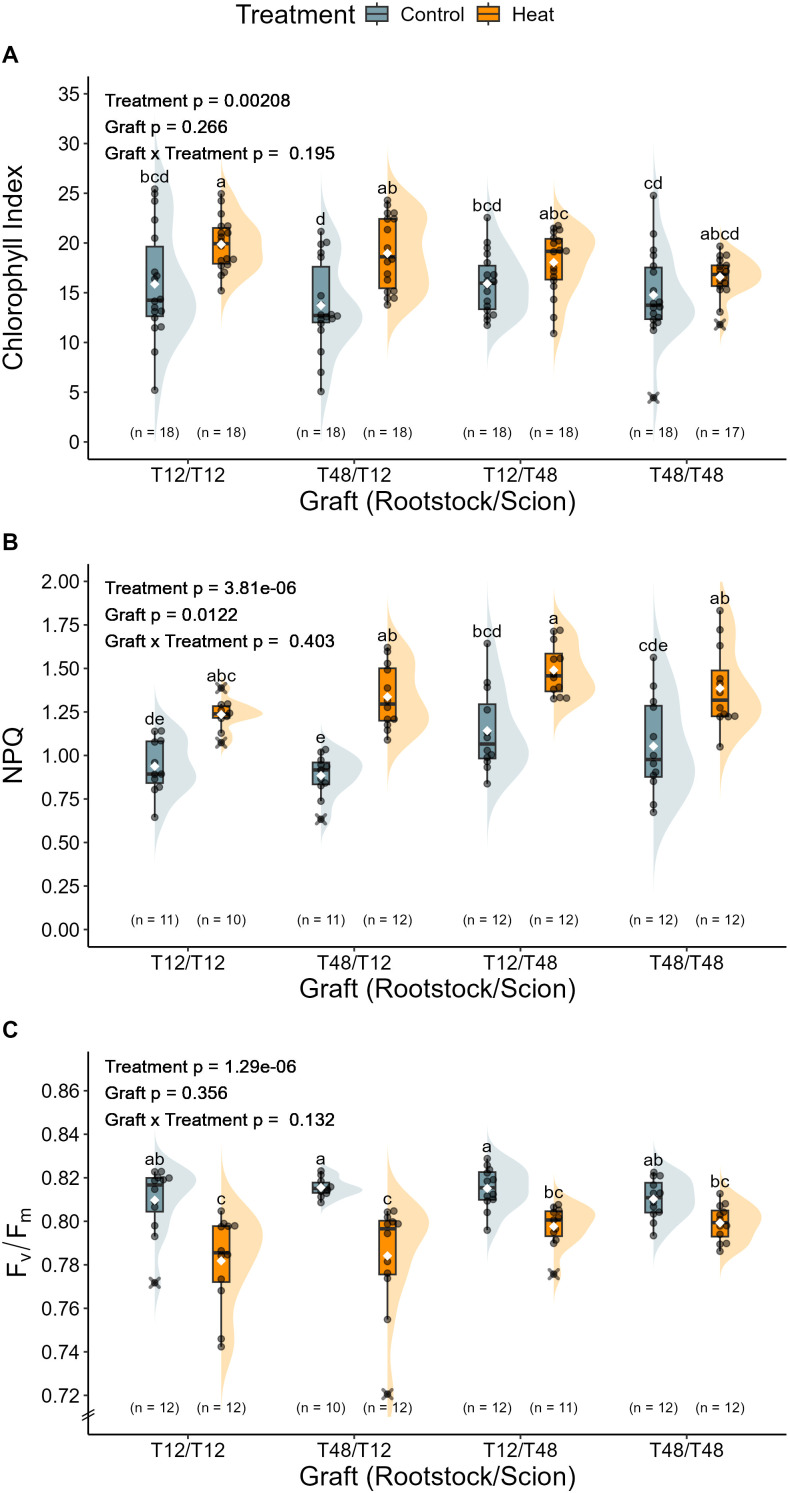
Photosystems of grafted tomato plants were mainly affected by heat stress. Influences on the photosystem as predicted from transcriptomics data were investigated on three levels: **(A)** Chlorophyll Index, **(B)** Non-photochemical quenching (NPQ), and **(C)** Maximum quantum yield of PSII (F_v_/F_m_). The grafted tomato plants were cultivated and treated under greenhouse conditions. Measurements of the chlorophyll content were performed during the 66^th^ day of treatment and NPQ as well as F_v_/F_m_ during the 35^th^ day of treatment. Measurements were taken on the marked leaf that was already developed before the treatment period. A two-way ANOVA followed by Tukey’s HSD *post-hoc* test was used to analyse statistical significance. NPQ and F_v_/F_m_ data was normalised and the ANOVA models for chlorophyll and NPQ included a White-correction for statistical analysis. Significant differences are indicated by different letters for the interaction Graft x Treatment (p < 0.05), while the level of significance for the factors is indicated as text element in the graph. The number of biological replicates after outlier removal is noted above the x-axis.

To obtain further insight in the physiological implications of long-term HS, influences on the leaf chlorophyll content as well as selected characteristics of the photosystem were investigated. In tomato leaves of grafts with a T12 scion, a significant increase of chlorophyll content was measured under HS conditions. This was resembled by an increase of the chlorophyll index from an average of around 14.8 to 19.4. The same tendency was observed for T48 scions, which were measured with a chlorophyll index value of 15.3 under control conditions and 17.3 under HS conditions (**Figure 8A**). However, the magnitude of change was not significant.

To check whether dissipation of excess light energy via non-photochemical quenching (NPQ) increased due to the HS treatment, measurements of the maximum fluorescence yield were taken. NPQ was mainly governed by the scion and was overall significantly influenced by the graft and the treatment (**Figure 8B**). The lowest values for NPQ were measured for T12 scions, which on average were recorded with values of 0.91 under control and 1.29 under HS condition. Scions of the genotype T48 displayed slightly higher values on average than T12. Under control conditions NPQ of T48 scions was at 1.10 and at 1.44 under HS conditions.

To investigate the functionality and condition of the photosystem, the maximum quantum yield of photosystem II (F_v_/F_m_) was determined. Of all tested graft combinations, none departed from the theoretical optimal F_v_/F_m_ of around 0.8 under control conditions, as the plants displayed values of around 0.813 under control conditions (**Figure 8C**). HS on the other hand, caused a significant reduction to values of around 0.783 in T12 scions, and 0.798 in the T12/T48 graft and non-significant reductions to 0.80 in T48/T48. The respective difference between control and HS conditions indicates a moderate impairment of maximum quantum yield.

## 4 Discussion

### 4.1 Harnessing genetic diversity in tomato for heat stress tolerance

To harness the genetic diversity of tomato landraces and use their untapped genetic potential for breeding against abiotic stresses it is paramount to identify suitable germplasm (Zhang et al., 2017; Fernie and Yan, 2019). Driven by decades of breeding, genetic diversity of vegetative traits underwent bottleneck events (Harlan, 1975; Tanksley and McCouch, 1997; Zhang et al., 2017; Fernie and Yan, 2019). Those eventually led to modern elite tomato cultivars that produce good yields but are prone to environmental changes and abiotic stresses when compared to wild tomato species (Bolger et al., 2014b; Wolter et al., 2019; Bhandari et al., 2023). Instead of further optimization of yield under optimal conditions, climate change forces the breeding industry to adapt crops to stressful abiotic conditions to ameliorate predicted yield losses (Hazra et al., 2007; Ayenan et al., 2019). In the past, simulations of tomato yield under elevated average daily temperatures, predicted yield loss potential of up to 85% (Ayankojo and Morgan, 2020), which has been supported by experiments investigating the effect of HS on tomato yield and this study (Rainwater et al., 1996).

### 4.2 Identification of heat stress tolerant genotypes through phenotypic screening

To overcome the limitations of genetic diversity in modern cultivars and to support the reintroduction of neglected traits from tomato landraces, this study aimed to identify HS tolerance in a screening population of 56 tomato genotypes and to investigate the transferability of tolerance-related traits by grafting. Therefore, three steps were taken: Screening of a diversity panel for HS tolerance and potential yield loss, investigation of potentially affected traits in grafted plants, and evaluation of the performance of grafted plants under greenhouse conditions. The latter included testing traits in old tomato plants, focusing on those possibly affected by HS and observed initially in young plants. These trials were conducted at control and elevated temperatures that exceeded the range of optimum growing temperatures during the day and night. The reported optimal temperatures for tomato growth are between 21°C and 26°C during the day and between 15°C and 20°C during the night (Hazra et al., 2007). The control conditions had temperatures of 22/18°C (day/night) and were therefore within the optimal temperature range. The rationale for the HS temperature conditions of 35/25°C (day/night), which resemble a moderate HS (Ayenan et al., 2019; Mishra et al., 2023), was that many tomato genotypes can enter developmental arrest phases at temperatures above 35°C and display severely affected reproductivity at temperatures exceeding 35°C (Berry and Uddin, 1988). Therefore, the intensity of the stress was resembled by the duration of its application and favoured the study of adaptations to long-term HS (Lichtenthaler, 1998).

The identification of two tomato genotypes with contrasting HS tolerance among the screened diversity panel of 56 accessions was essential for subsequent grafting experiments and focused on vegetative and flowering traits. After the application of HS for 20 days, plants grown under HS conditions displayed a developmental advance over the control treated plants, as judged by the average number of trusses which coincided with an increased number of developed flowers per truss (**Supplementary Tables S5**, **S7**). These observations highlighted the likelihood of the heat-stressed plants reaching the BBCH principal growth stage seven (development of fruits) earlier (Feller et al., 1995; Meier, 2018). The advancement in development might be explained by the higher average daily temperature which is supported by the concept of thermal time (Monteith, 1977; Heuvelink, 1989; Boote et al., 2012). As previously discussed, the treatment conditions were designed to remain within boundaries that allow for the growth of tomato plants (Maynard and Hochmuth, 2006; Jones, 2007; Wahid et al., 2007). Considering that plant growth is expected to continue as long as the intensity and duration of a stressor remains below the individual threshold for acute damage and within the phase of maximal tolerance (Lichtenthaler, 1998), it is likely that the measurements were taken after the plants had adapted to the stress and resided in the stage of tolerance. To investigate whether adaptation to elevated temperatures influenced growth, dry weight was investigated. In addition, this measure was crucial for assessing stress tolerance, since the ability to sustain and build up biomass are integral parts of the definition of stress tolerance (Thiry et al., 2016). Despite the observed developmental advantages, biomass accumulation was significantly diminished by the HS. While this effect has been observed in other studies (Giri et al., 2017; Poudyal et al., 2019; Mukhtar et al., 2020), some genotypes displayed a behaviour contrary to the global trend and maintained similar amounts of dry weight under both temperature conditions (**Figure 2A**), which made them excellent candidates for tolerant genotypes, and *vice versa*. Among the examined tomato plants, genotypes T12 (tolerant) and T48 (susceptible) were identified. As the selected genotypes were expected to be used in reciprocal grafting experiments, root biomass was also investigated, as it has been successfully used as predictor for superior rootstocks (Colla et al., 2017; Singh et al., 2017). Here, the tomato genotype T12 exhibited an overall high amount of root biomass (**Figure 2A**), which supported the choice to investigate it as a tolerant genotype (Colla et al., 2017; Singh et al., 2017).

The necessity to conduct the screening under controlled environmental conditions limited the duration of the HS treatment of the initial screening experiment and led to its termination in spring, in order to circumvent unstable temperature conditions in the control cabins (**Supplementary Table S2**). Consequently, data on flower development and abscission were utilised instead of yield data to predict the effects of temperature treatment as a proxy for potential future yield loss. Flower abscission has been employed in different studies to investigate the influence of abiotic stress as a predictor of future yield loss (Berry and Uddin, 1988; Peet et al., 1997; Reichardt et al., 2020). In our studies, some genotypes indicated the potential of up to 30% future yield loss (**Figure 2B**). However, the absence of abscission in T12 and the incidence of up to 14.8% flower abscission in T48 supported the classification of T12 as HS tolerant and T48 as HS susceptible.

### 4.3 Grafting as a tool for trait transfer and stress adaptation

Following the identification of two genotypes of contrasting HS tolerance, reciprocal grafting experiments were conducted to investigate HS adaptation and the transferability of HS tolerance by grafting. Given that grafting is an accessible horticultural technique that has been utilised to increase resistance towards biotic stressors and tolerance against abiotic stresses (Schwarz et al., 2010; Keatinge et al., 2014), it is notable that many of the molecular changes it causes remain elusive (Mahmoud, 2020; Tsaballa et al., 2021). Consequently, two lines of research were pursued. The first was an investigation of molecular adaptations to HS by transcriptomics, while the second was an analysis of the effects of grafting on selected phenotypic traits and yield.

The study of molecular adaptations to HS and grafting was focused on leaf tissue, since many of the adaptations to plant metabolism affect the photosynthetic active tissue (Wahid et al., 2007; Balfagón et al., 2020), which ultimately provides the energy used by plants to grow and develop reproductive organs (Asseng et al., 2002). A total number of 2800 to 5879 genes has been observed to be differentially expressed in leaves after seven days of stress in young plants, indicating considerable adaptations to HS (**Figure 3**). These numbers fit in the range of previous observations (Ding et al., 2020a; Psaroudakis et al., 2024). Among the DEGs identified in all graft combinations, a core response of genes with a highly similar expression pattern has been identified (**Figure 3** and **Figure 4**). Similar to previous studies on HS adaptation, prominent markers such as members of the heat shock protein (HSP) family and a related heat shock transcription factors were induced (Guo et al., 2016; Yang et al., 2016; Jacob et al., 2017). Overall, roughly 50% of a core response observed in a meta-analysis of diverse tomato tissues exposed to different intensities and durations of HS (Psaroudakis et al., 2024), were resembled in the presented data sets (**Figure 4**). Similar to the meta-analysis by Psaroudakis et al. (2024), GO-enrichment analysis supported viability of the highly affected gene set in cluster 1, by highlighting the enrichment of terms such as “heat acclimation” (BP), “protein self-association” (MF), “unfolded protein binding” (MF) (**Figure 5**). Eventually, differential expression of TFs, belonging to the classes of WRKY, bZIP, HSF and MYB has been revealed by RNA-Seq (**Supplementary Table S6**). These may have orchestrated the observed changes, as they have been identified as key players in response to HS (Mittler et al., 2012; Guo et al., 2016; Balyan et al., 2020; Ding et al., 2020b). In addition, TFs and TRs of classes like ARID, DDT, GNAT, and Jumonji were identified (**Supplementary Table S6**), which are prominent for their influence on chromatin structure (Mazzio and Soliman, 2012; Gan et al., 2015), likely have been supporting the global changes in gene expression that were observed in the leaves of tomato plants. Consequently, these TFs may be suitable targets for breeding and genetic engineering of vegetative traits (Oberkofler and Bäurle, 2022).

Among the repressed genes, GO-enrichment analysis revealed that terms indicating an involvement of the photosystem have been affected by the HS treatment (**Figure 5**). This is in line with the previously described impairment of photosynthetic activity and influences on the chloroplast as one major HS adaptation response (Wahid et al., 2007). Despite the overall adaptation response towards HS observed in any graft, only a few DEGs were identified as potentially influenced by grafting (**Figure 3**). Therefore, it is noteworthy that GO terms belonging to photosynthesis-related categories, were also enriched in a gene set of repressed genes that were exclusively shared by plants with a T48 rootstock (**Supplementary Figure S4**), indicating the possibility of a trait that has been influenced by grafting (Schwarz et al., 2010). Thus, photosynthetic traits such as the maximum quantum efficiency, which has been employed to successfully identify HS tolerant tomato genotypes (Poudyal et al., 2019), were drawn into focus as target for further investigation in the subsequent long-term grafting experiment. Here, reciprocally grafted tomato plants were subjected to HS conditions for a period of 11 weeks.

### 4.4 Physiological implications of grafting

As abiotic stresses can adversely influence photochemistry, causing alterations to the dissipation of excess light energy (Baker, 2008), and gene expression data obtained from leaf tissue highlighted potential influences of the grafting and the HS treatment on the photosystem (**Figure 5**) a closer look was taken on maximum quantum efficiency (F_v_/F_m_) and non-photochemical quenching (NPQ) in plants exposed to HS in the long-term grafting experiment. Since all grafting combinations showed significantly decreased F_v_/F_m_ values upon HS treatment, besides T48/T48, an influence of HS on the efficiency of the photosystem can be concluded (**Figure 8C**). Previous studies of HS in tomato plants have observed similar influences on the maximum quantum efficiency (Camejo et al., 2005; Poudyal et al., 2019). In contrast to expectations, the observed decline in F_v_/F_m_ in tolerant T12 scions is not in line with the anticipated impact of HS on F_v_/F_m_ in tolerant genotypes. This observation may be attributed to the short period of time considered for dark adaptation (Baker, 2008) and the observed increase in non-photochemical quenching across all graft combinations (**Figure 8B**). Consequently, it would be beneficial to conduct further experiments utilising adapted experimental setups that consider the impact of HS in naturally dark-adapted states. Yet, it is noteworthy that an elevated chlorophyll index has been observed in leaves following HS (**Figure 8A**). This phenomenon may be indicative of an adaptive process to cope with adverse influences on photochemistry, as previous studies on the impact of long-term HS have also observed an increase in chlorophyll content (Bhattarai et al., 2021; Lee et al., 2023).

An additional indicator of the presence and severity of HS at the cellular level was the membrane permeability observed for leaf tissue (Camejo et al., 2005). Here, an overall significant increase in ion leakage was observed upon HS treatment, indicating the presence of stress at the cellular level for all graft combinations (**Figure 7**). Increased membrane permeability upon HS causes a cascade of processes that include the accumulation of ROS and support the recruitment of HS proteins (Graci and Barone, 2024), which correlates well with the increased expression of genes encoding enzymes related to redox metabolism and HS as observed in leaf tissue of younger plants (**Figure 4**) (Kapoor et al., 2015).

### 4.5 Validation of screening results and agronomic implications of grafting

Another main objective of the long-term grafting experiment was the validation of the assigned tolerance levels of the tomato genotypes T12 and T48, as determined by the screening data. For this purpose, biomass and yield data from self-grafted plants were considered, which eventually confirmed the HS tolerance of genotype T12 and the HS susceptibility of T48. Overall, the biomass data were consistent with the observations from the screening, with T12 self-grafts displaying an increased biomass and T48 self-grafts simultaneously displaying a diminished biomass (**Figure 2A** and **Figure 6A**). Again, these findings were in line with previous studies on HS tolerance (Giri et al., 2017; Poudyal et al., 2019; Mukhtar et al., 2020). Hence, the classification based on the criterium of growth were consistent with the definition of stress tolerance (Thiry et al., 2016).

As information on yield is paramount to investigate stress resilience (Davies and Ribaut, 2017), which is a crucial trait for breeding (Harfouche et al., 2019), the predicted influences on yield loss by flower abscission rate (**Figure 2B**) had to be investigated too. While the percentage of flowers dropped during the screening was generally low, long-term HS caused pronounced implications on fruit development and yield for both tomato genotypes (**Figure 2B**, **Figures 6B, C**). Here, plants of the T48 genotype exhibited a markedly reduced yield of as little as 3% and a relative number of fruits of 4% compared to control plants, indicating that the genotype is susceptible to high temperatures. In contrast, tomato plants of the T12 genotype maintained a yield of approximately 49% and 37% of the fruits, which highlights the superior HS tolerance over T48. Regardless the pronounced effects, the results from self-grafted tomato plants were consistent with those observed during the screening.

When reflecting possible causes for the observed differences in yield and the number of fruits produced by the two genotypes, viability of pollen might play a considerable role. While pollen viability has not been a direct focus of this study, the number of fruits as well as yield might serve as surrogate markers (**Figures 6B, C**). Here the constant low amount of yield and number of fruits observed in scions of T48 might suggest that pollen viability has been impaired considerably by the treatment. Studies by Peet et al. (1997, 2003) and Sato et al. (2000) suggest that the underlying cause for this might reside in a genotype dependent susceptibility of pollen towards increased mean daily temperatures or altered VPD, respectively. A study by Abdelmageed and Gruda (2009) showed that grafting can affect production of pollen grains. Whether the observed decrease in the number of fruits and yield in the T48/T12 has been a result of altered pollen viability, remains to be answered in future studies.

From literature and practical applications, it is well known that grafting can influence yield (Lee et al., 2010; Schwarz et al., 2010; Kyriacou et al., 2017). For this reason, superior rootstocks such as ‘Maxifort’ and ‘Beaufort’ are commonly used in commercial tomato production, as they have been proven to boost yield and longevity (Mišković et al., 2009; Lee et al., 2010; Kyriacou et al., 2017). In the present study, grafting seemingly affected yield as well, since yield data of the reciprocally grafted plants indicated a significantly negative influence of the susceptible T48 rootstock on the number of tomatoes and amount of yield produced from T48/T12 plants under either temperature conditions (**Figures 6B, C**; **Table 1**). This observation also holds true for the investigated yield-based tolerance indices, which displayed an impaired persistence and HS tolerance of T12 scions in combination with a T48 rootstock when compared to the T12 self-graft (**Table 1**). Hence, the HS susceptibility of T48 rootstocks might have decreased the thermotolerance of T12 scions, which makes this heterograft a particularly interesting grafting combination to explore the potential cause for the impaired yield.

### 4.6 Outlook

Recapitulating the above-mentioned effects of HS on biomass, yield, and gene expression in the reciprocal grafts, under the premise that a T48 rootstock might have been able to influence the productivity of a grafted scion, the following two observations could be relevant for further investigations: First, genes contributing to photosynthesis-related GO terms were repressed in leaf tissue of grafts with a T48 rootstock, potentially affecting the overall energy availability in the plants (**Figure 5**). Second, while no significant difference in the overall biomass of the vegetative tissue was observed for either graft under HS conditions (**Figure 6A**), differences in yield were recorded (**Figure 6B**). This eventually suggests that energy partitioning for tissue development or flowering traits might have been modified by the grafting.

To further elucidate the impact of grafting, future studies should focus on a targeted analysis of genes, proteins, hormones, and metabolites involved in energy partitioning, development, and flowering modulation. For this, a higher number of biological replicates can be recommended to enhance the statistical power for the detection of small changes. Although this was beyond the scope of the current study, such investigations could provide a deeper understanding of the observed macro-effects.

### 4.7 Conclusion

In conclusion, this study successfully identified two tomato genotypes with contrasting HS tolerance from a screening population of 56 accessions, which was limited to simple vegetative and flowering traits. The selected genotypes, T12 (tolerant) and T48 (susceptible), were used in reciprocal grafting experiments, where self-grafted plants confirmed the tolerance classification based on biomass accumulation, yield, and related stress tolerance indices. The findings suggest that the workload in selecting HS-tolerant genotypes can likely be reduced by focusing on early developmental stages. However, the study also highlights that the predictability of grafting effects may not always align with genotype-specific tolerance characteristics.

To investigate the transferability of HS tolerance, transcriptomic data were obtained from grafted plants. While other studies have focused on transcriptional changes in reproductive tissues, our study examined the adaptation of leaf tissue in young, grafted tomato plants to HS under strictly controlled environmental conditions. The overall observed adaptations to HS corresponded well with existing meta-studies on HS core responses in tomato leaves, highlighting the importance of heat shock proteins and ROS detoxification processes, as well as the modulation of energy-consuming developmental processes and photosynthesis. The nuanced changes in the transcript abundance of key TFs and TRs, involved in chromatin remodelling, such as ARID, DDT, GNAT, and Jumonji, suggest that further systemic investigation is needed to foster the understanding of HS adaptation and tolerance in tomato, as the related genes might serve as valuable targets for breeding of heat-adapted tomato genotypes.

GO-enrichment analysis revealed a potential effect of grafting on the chloroplasts and photosynthesis, respectively, which have been influenced by the susceptible rootstock (T48). However, a long-term experiment indicated that the main factor affecting surrogate stress markers of the photosystems, such as NPQ and F_v_/F_m_, was the treatment itself. Interestingly, the long-term experiment with reciprocal grafts of T12 and T48 tomatoes revealed alterations in yield, suggesting a modulation of HS tolerance by rootstocks. In this case, the susceptible rootstock (T48) reduced the yield from tolerant scions (T12).

Taken together, this study provides a foundation for future exploration of the underlying mechanism and underscores the need for systemic investigations that could enhance targeted rootstock selection for the modulation of complex traits.

## Data Availability

Sequencing raw data can be accessed from the NCBI repository using the BioProject ID PRJNA1194332 (https://www.ncbi.nlm.nih.gov/sra/PRJNA1194332).
