# Supplementary material for: Strike at the root: exploring the transferability of heat stress tolerance in tomatoes by reciprocal grafting
Source: Front Plant Sci. 2025 Jul 24;16:1549737. doi: 10.3389/fpls.2025.1549737 (PMC12328372; doi:10.3389/fpls.2025.1549737)
Supplement: Supplementary file 1 [file DataSheet1.pdf]

Supplementary Material

1 Supplementary Data

Sequencing raw data can be accessed from the NCBI repository using the BioProject ID PRJNA1194332 (https://www.ncbi.nlm.nih.gov/sra/PRJNA1194332).

Additional data provided as graph or table are described in the respective section.

Dataset S1: Images of the 56 tomato genotypes under heat stress and control conditions.

2 Supplementary Figures and Tables

2.1 Supplementary Figures

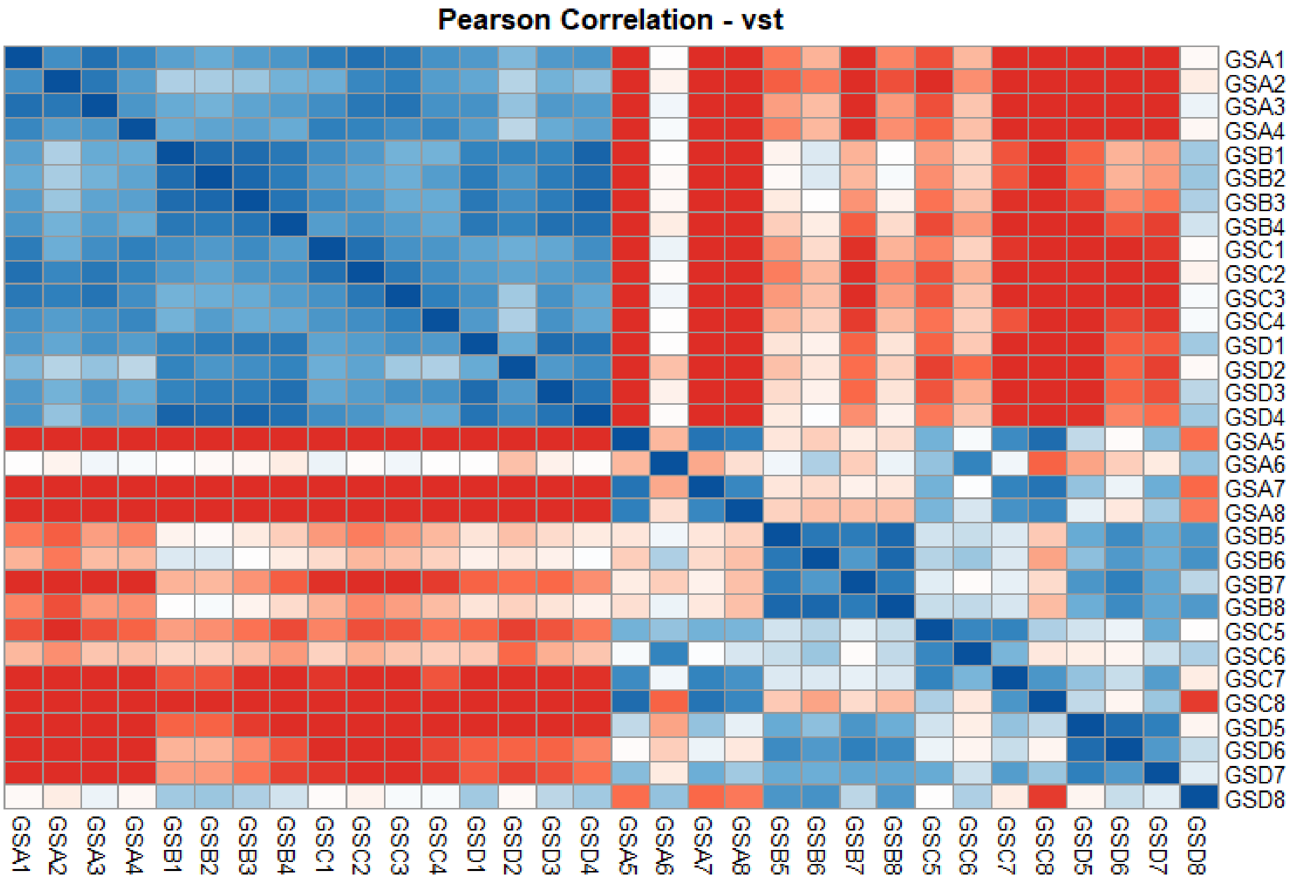

Figure S1: Pearson correlation of gene expression in all samples: Heat map of the correlation matrix of all samples using pairwise Person correlation revealed two outliers (GSA6 and GSD8) which were excluded from further analysis. Heat map was generated with normalized gene expression values resulting from DESeq2 by applying variance stabilized transformation.

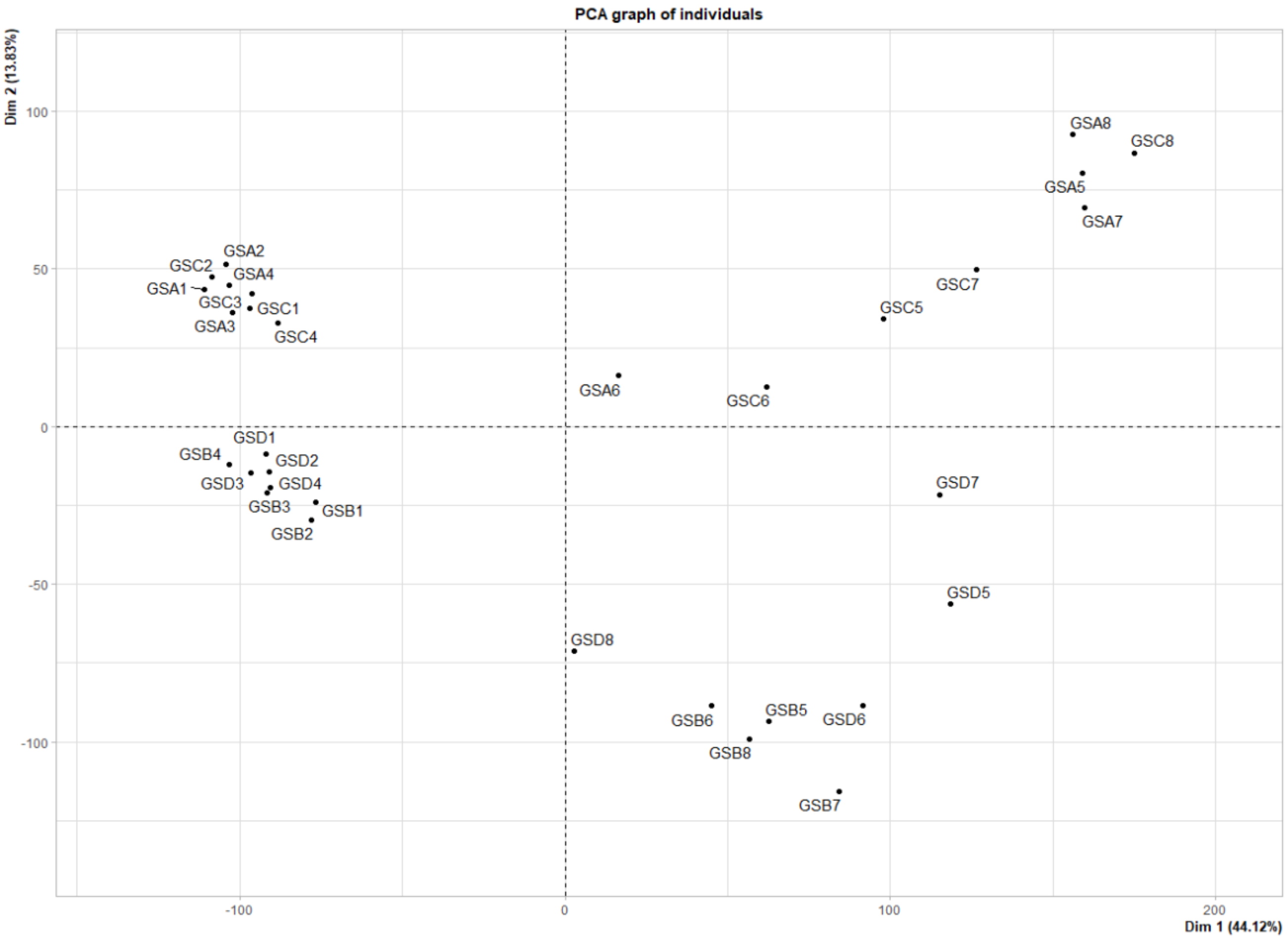

Figure S2: PCA of gene expression in all samples: PCA was generated with normalized gene expression data resulting from DESeq2 by applying variance stabilized transformation (vst). PCA confirmed the outliers GSA6 and GSD8 which were excluded from further analysis.

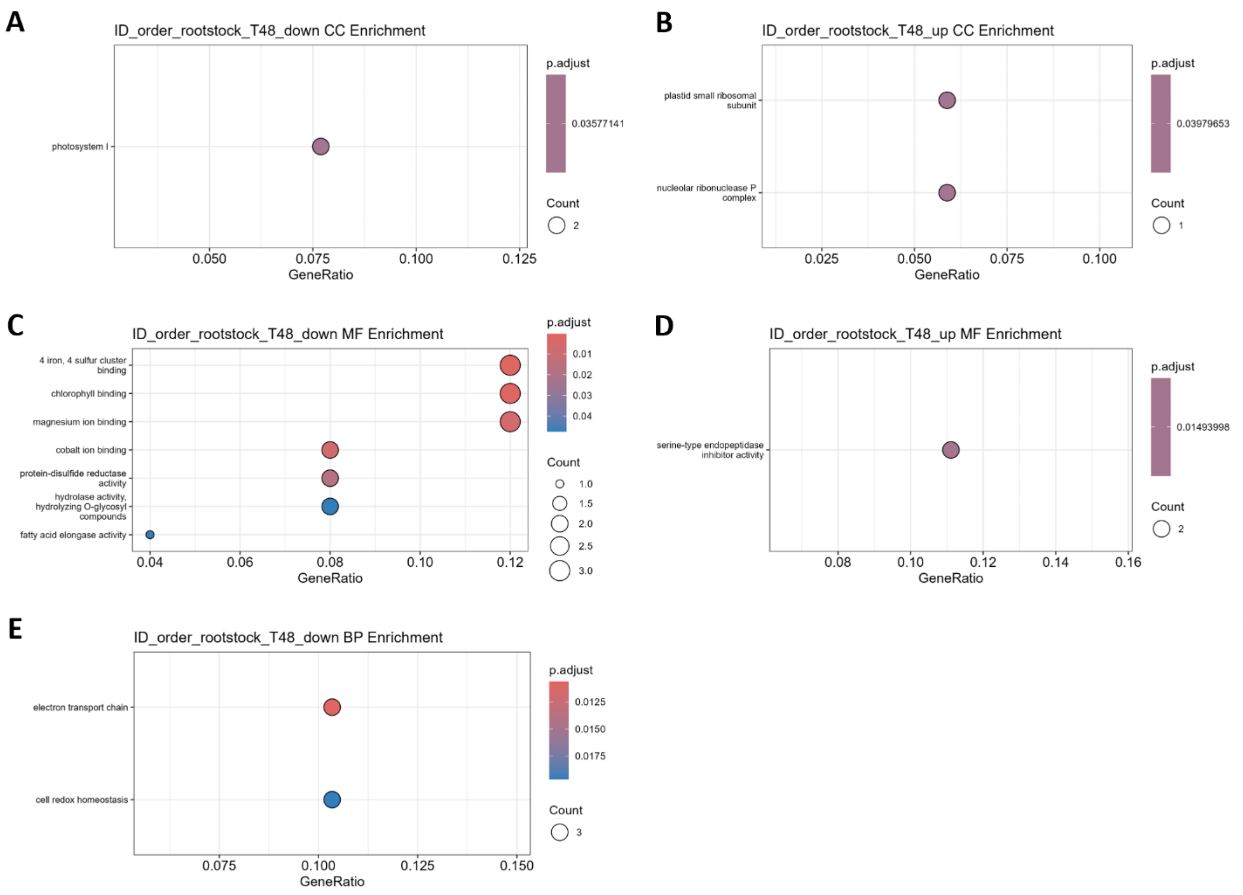

Figure S3: Gene Ontology (GO) enrichment of DEGs shared by grafts with a T48 rootstock. The panels represent significantly over-represented GO terms associated with biological processes (BP), molecular functions (MF), and cellular components (CC) for DEGs identified in grafts with a T48 rootstock, as indicated in the upset plot expression data (Figure 3). **A**, **B** CC enrichment for down- and upregulated DEGs, respectively. **C**, **D** MF enrichment for down- and upregulated DEGs, respectively. **E** enrichment for downregulated DEGs. No significantly enriched BP terms were identified for upregulated DEGs. The size of the circles indicates the number of genes associated with each GO term, while the colour gradient reflects the adjusted p-value (p.adjust) of the enrichment analysis.

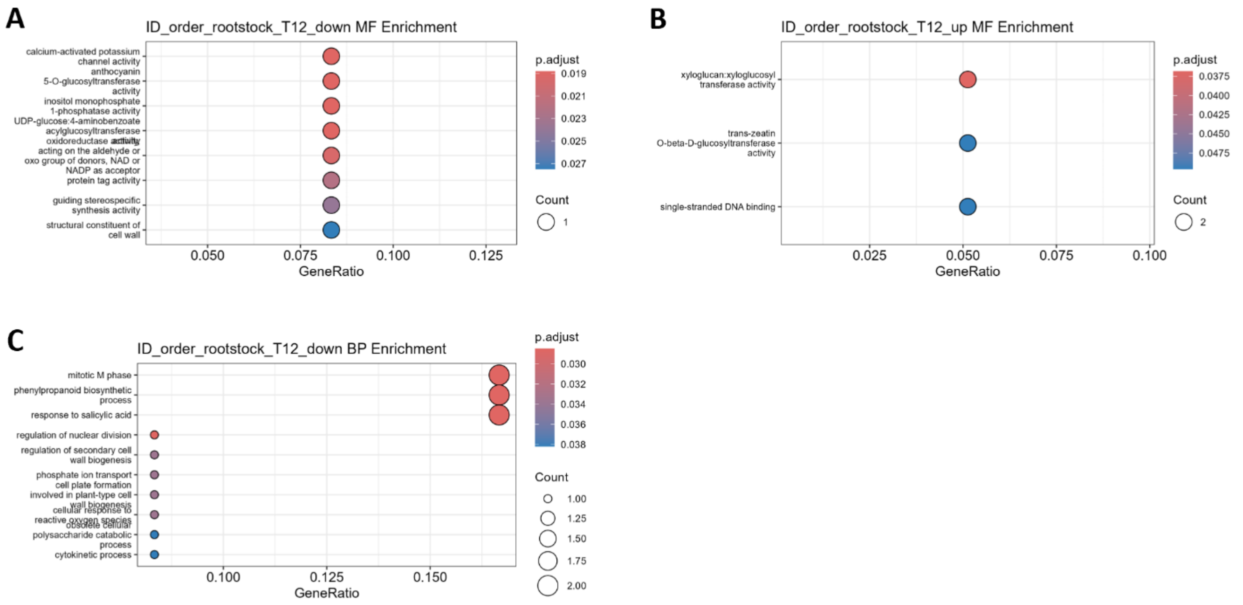

Figure S4: Gene Ontology (GO) enrichment of DEGs shared by grafts with a T12 rootstock. The panels represent significantly over-represented GO terms associated with biological processes (BP), molecular functions (MF), and cellular components (CC) for DEGs identified in grafts with a T48 rootstock, as indicated in the upset plot expression data (Figure 3). **A**, **B** MF enrichment for down- and upregulated DEGs, respectively. **C** BP enrichment for downregulated DEGs. No significantly enriched BP terms were identified for upregulated DEGs and for CC-related terms. The size of the circles indicates the number of genes associated with each GO term, while the colour gradient reflects the adjusted p-value (p.adjust) of the enrichment analysis.

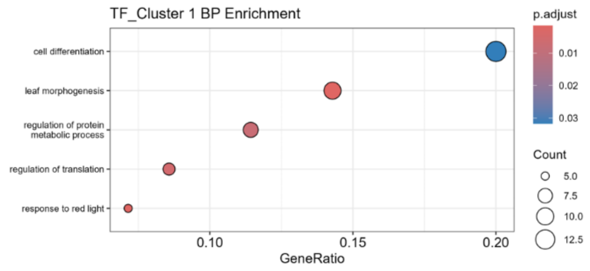

Figure S5: Gene Ontology (GO) enrichment of differentially expressed TFs and TRs reveals potentially influence of heat stress adaptation on cell development. Significantly over-represented GO terms associated biological processes (BP). No significant enriched terms were identified for molecular functions (MF), and cellular components (CC). The size of the circles indicates the number of genes associated with each GO term, while the colour gradient reflects the adjusted p-value (p.adjust) of the enrichment analysis.

2.2 Supplementary Tables

Due to size, all supplementary tables are provided as individual .xlsx files. Captions are provided below.

Table S1: Investigated tomato genotypes, denotation and primary source. Seeds of the tomato diversity panel were provided by AUA (Agricultural University of Athens, Greece), Culinaris (Culinaris – Saatgut für Lebensmittel, Germany), EGE (Ege University, Turkey), GAUTIER (Gautier Semences, France), INRA (Institut National de la Récherche Agronomique - Génétique et Amélioration des Fruits et Légumes, France), UIB (University of Balearic Islands, Spain) and UNITO (Università degli Studi di Torino, Italy) for the screening experiment. For the grafting experiments, seeds of T12 and T48 were produced and provided by GAUTIER. The variety and source code refers to the genotype identifiers used by the primary providers. The material type discriminates between commercial cultivars and genotypes obtained as landraces.

Table S2: Environmental conditions during the greenhouse experiments. Information on the recorded temperature, humidity, PAR and CO_2 concentration (only for the screening experiment) in the individual greenhouse cabins

Table S3: Settings of the LI-COR devices. Information on chamber as well as fluorescence measurement settings.

Table S4: Mapping Statistics. Mapping statistics for the RNA-Seq data.

Table S5: Development of trusses during the screening. Evaluation of mean truss development per genotype investigated during the screening experiment.

Table S6: Identified differentially expressed TFs and TRs, as well as DEGs per graft. Information on the gene names (ITAG4.1) for the TFs, TRs, and DEGs identified in leaf tissue of grafted tomato plants obtained within the transcriptomics experiment.

Table S7: Experimental raw data obtained during the screening experiment

Table S8: Experimental raw data obtained during the long-term grafting experiment.
