# Supplementary material for: Strike at the root: exploring the transferability of heat stress tolerance in tomatoes by reciprocal grafting
Source: Front Plant Sci. 2025 Jul 24;16:1549737. doi: 10.3389/fpls.2025.1549737 (PMC12328372; doi:10.3389/fpls.2025.1549737)

Supplemental Images

- This presentation contains images that should illustrate the investigated tomato genotypes during the screening experiment.

- Tomato genotypes as specified in the manuscript

- Treatment:
  - control
  - heat
  - Treatment conditions as specified in the manuscript

T01

control

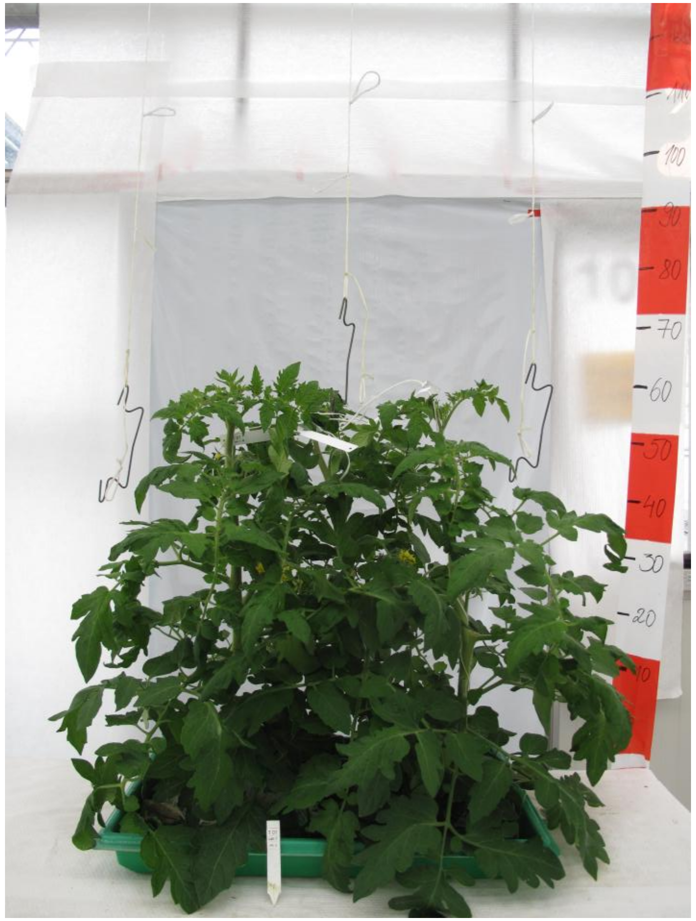

heat

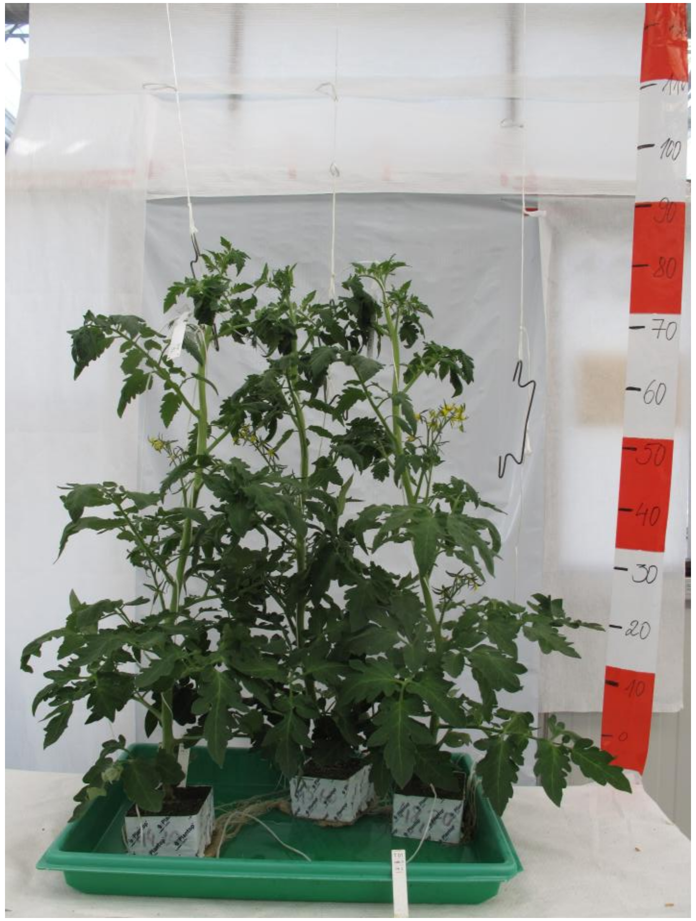

T02

control

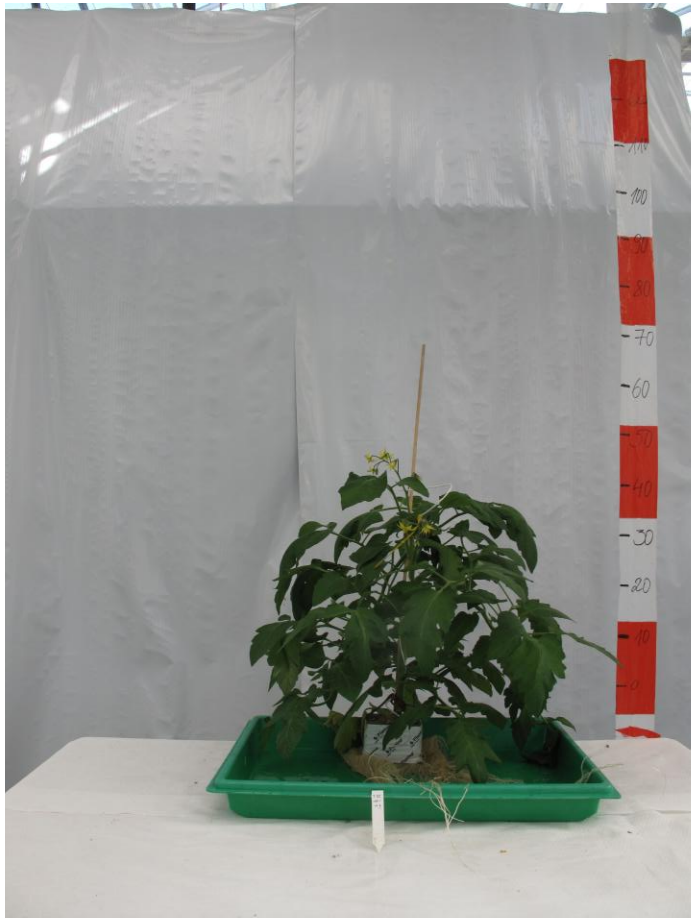

heat

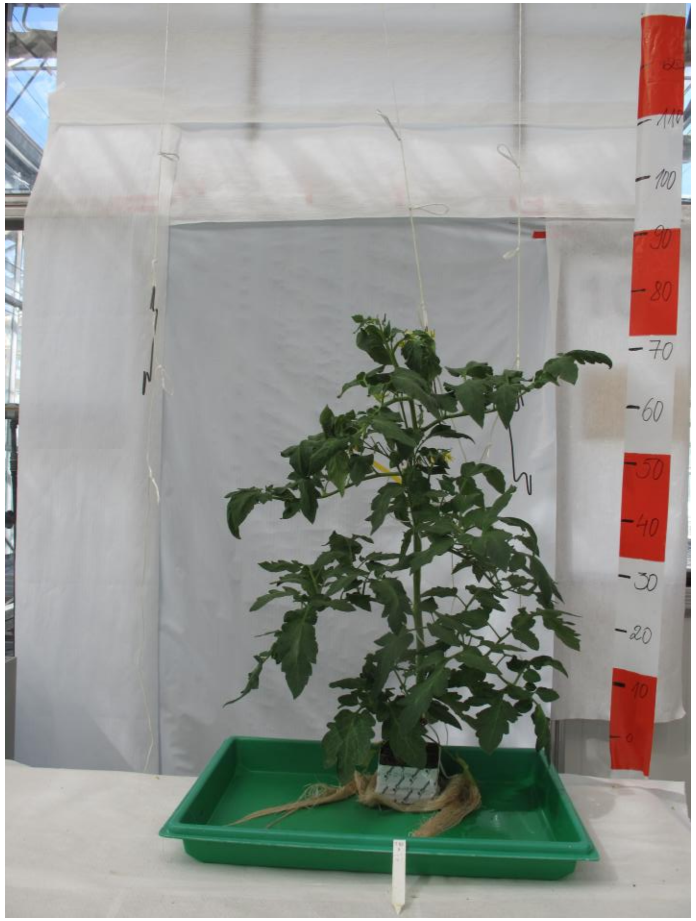

T03

control

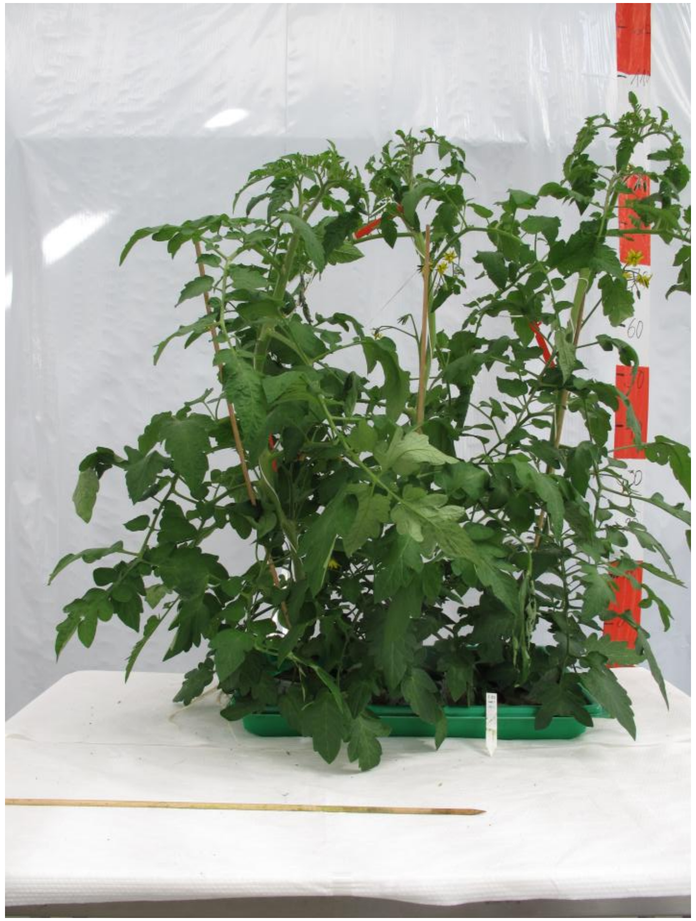

heat

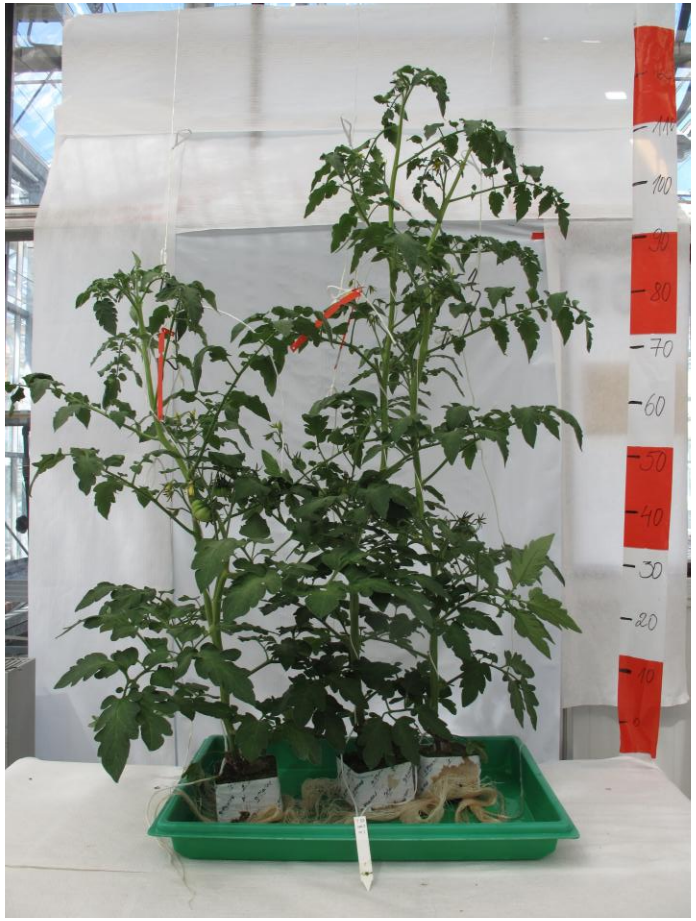

T04

control

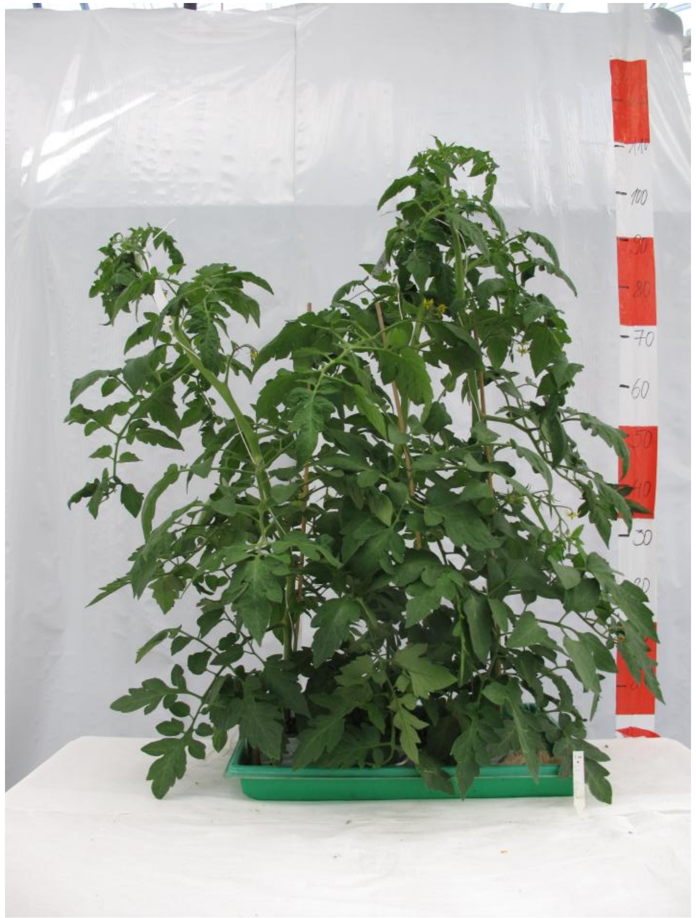

heat

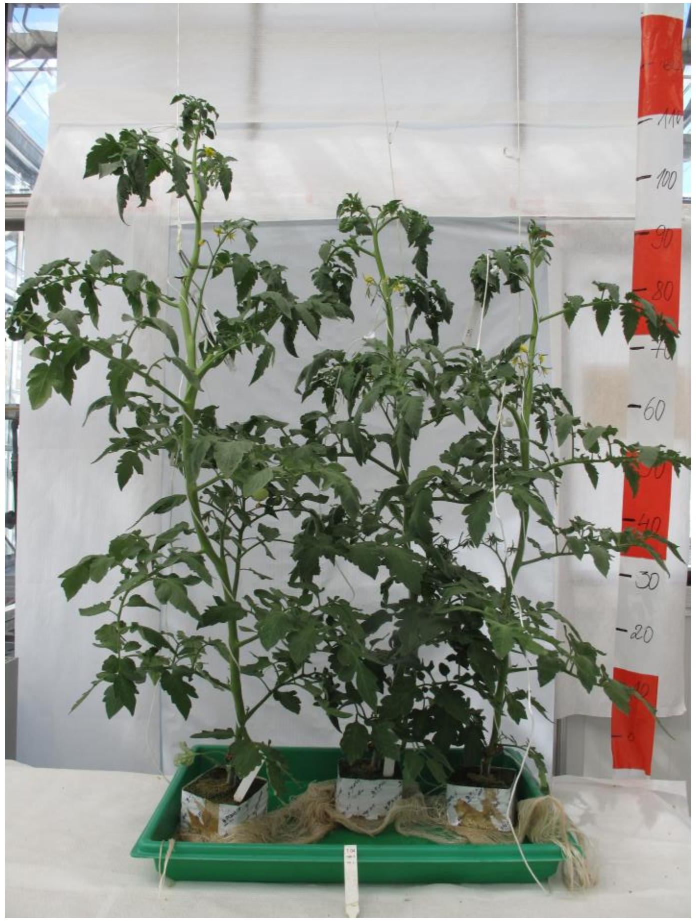

T05

control

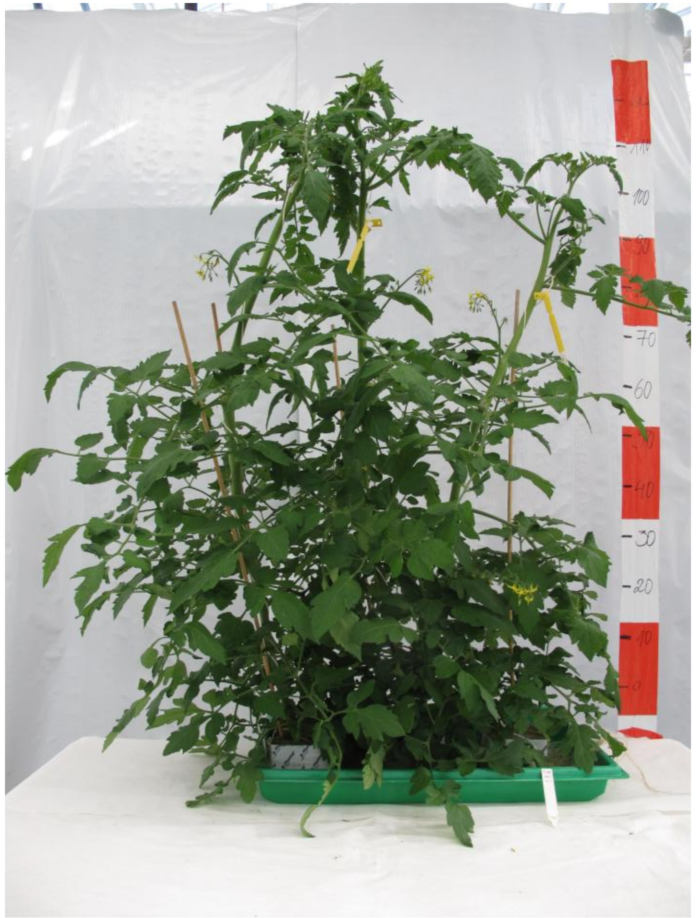

heat

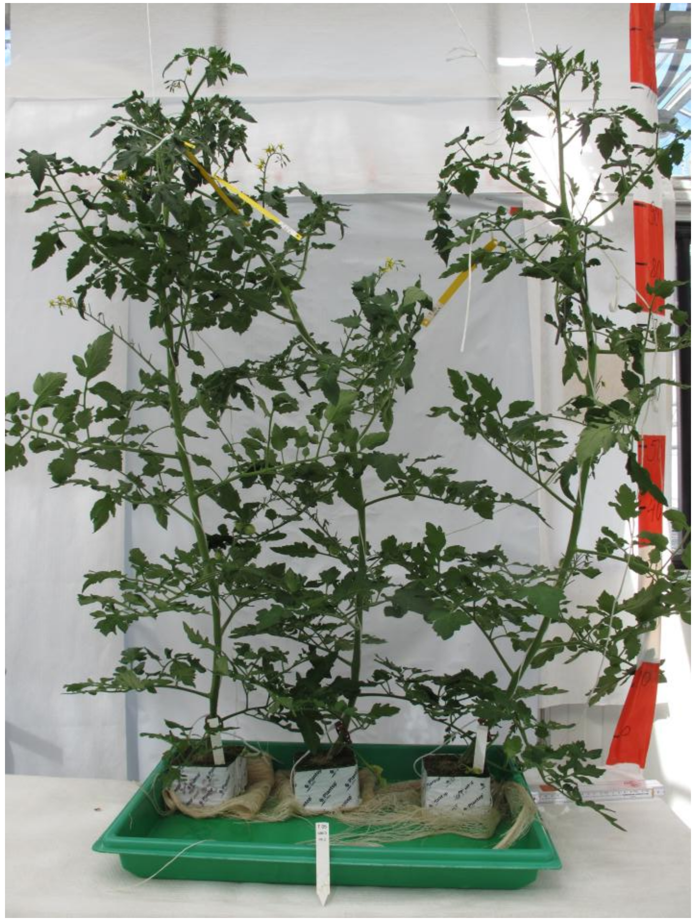

T06

control

heat

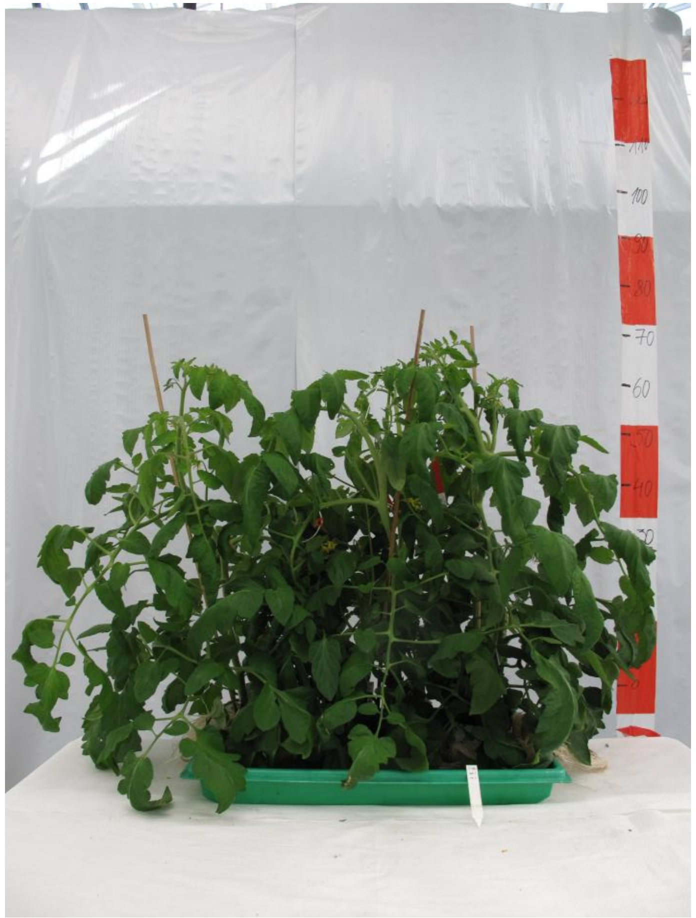

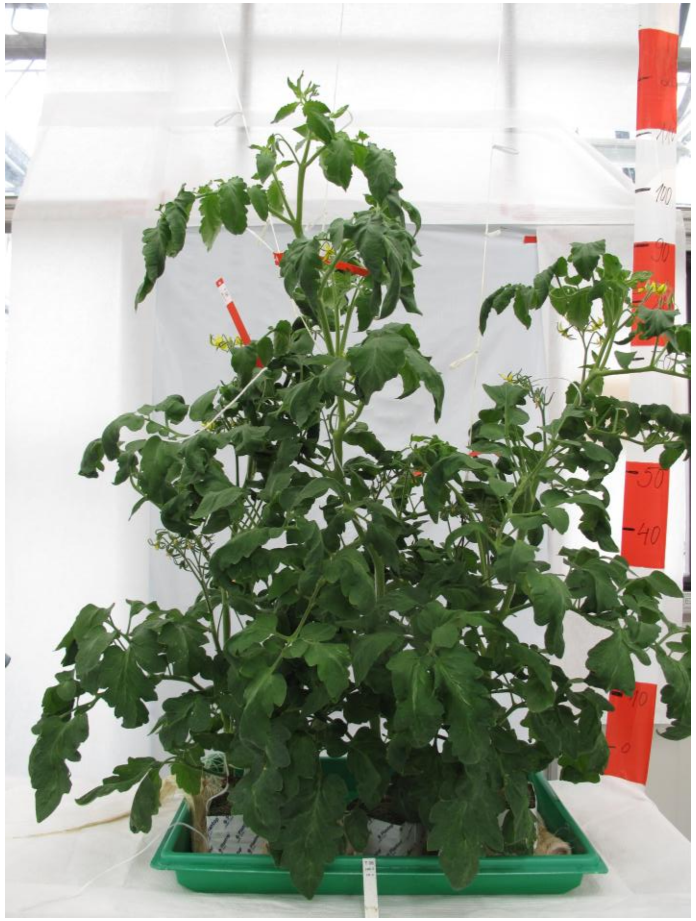

T07

control

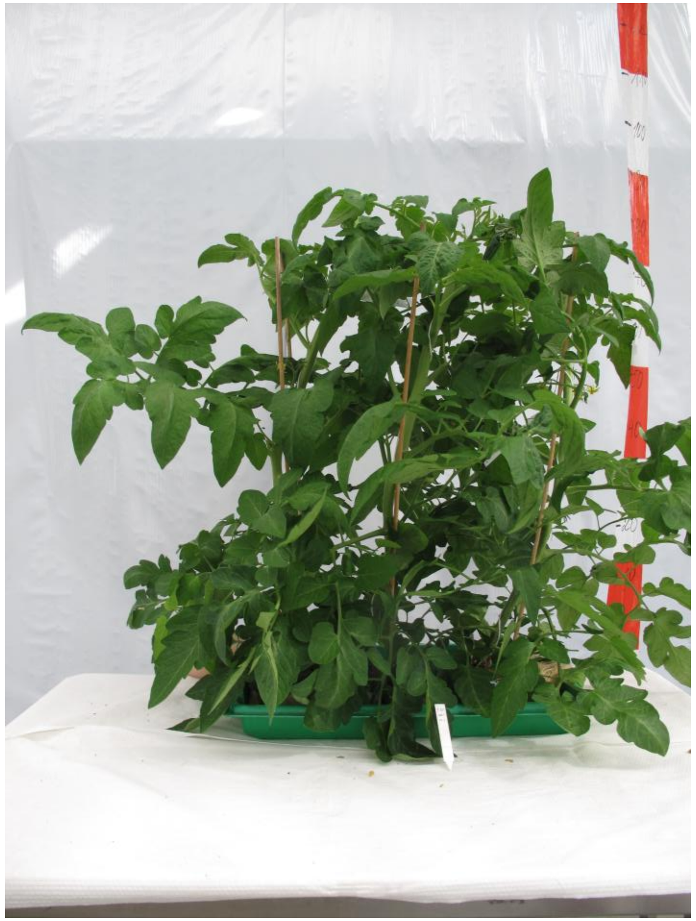

heat

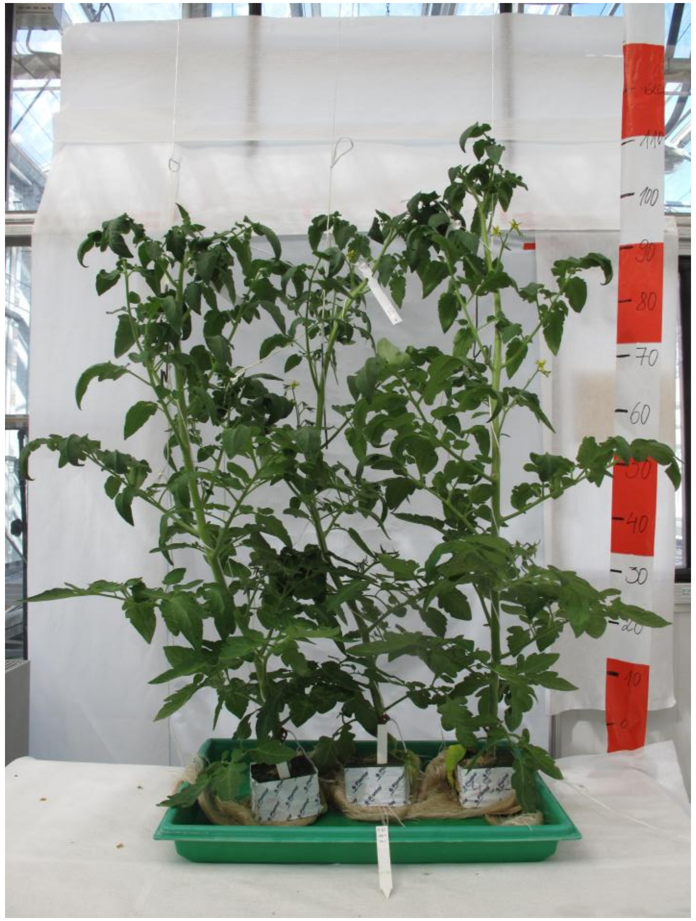

T08

control

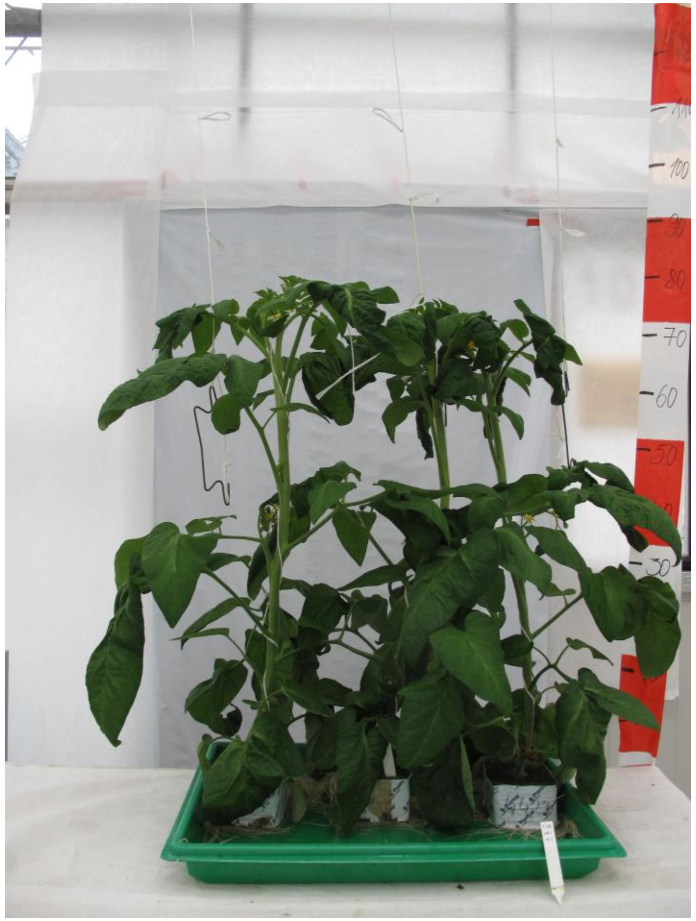

heat

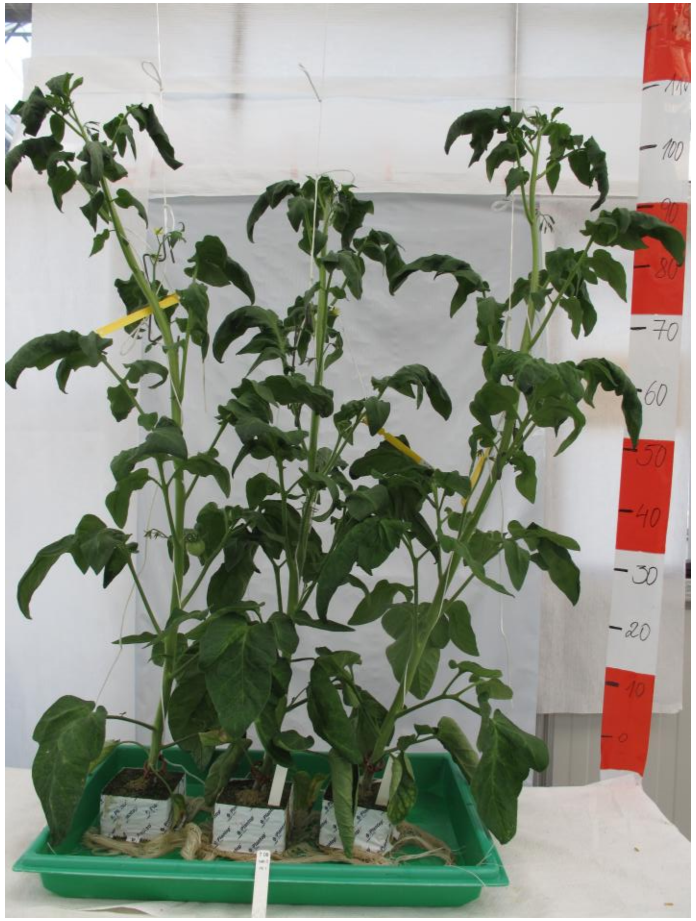

T09

control

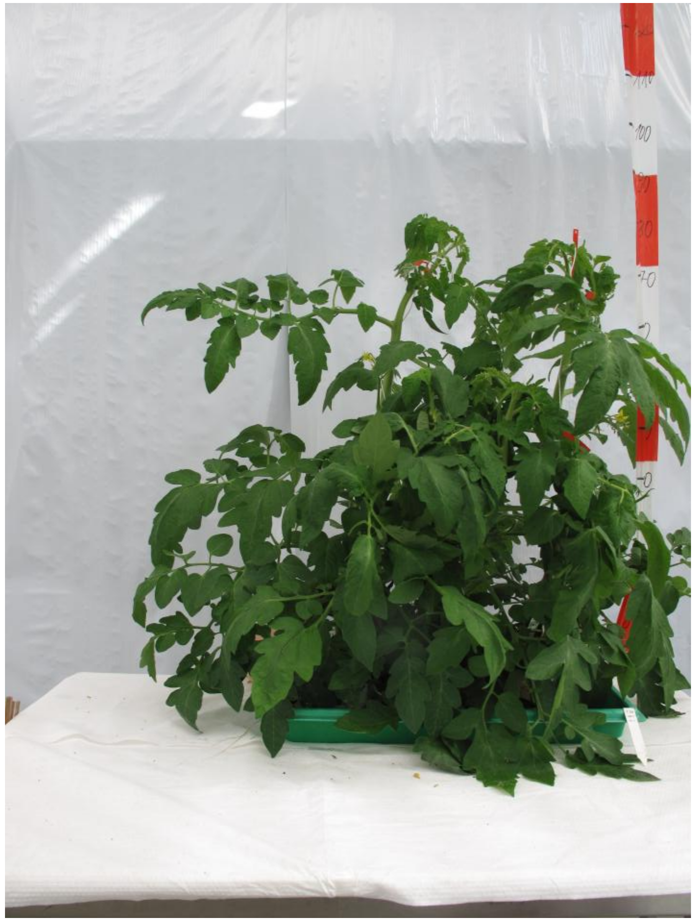

heat

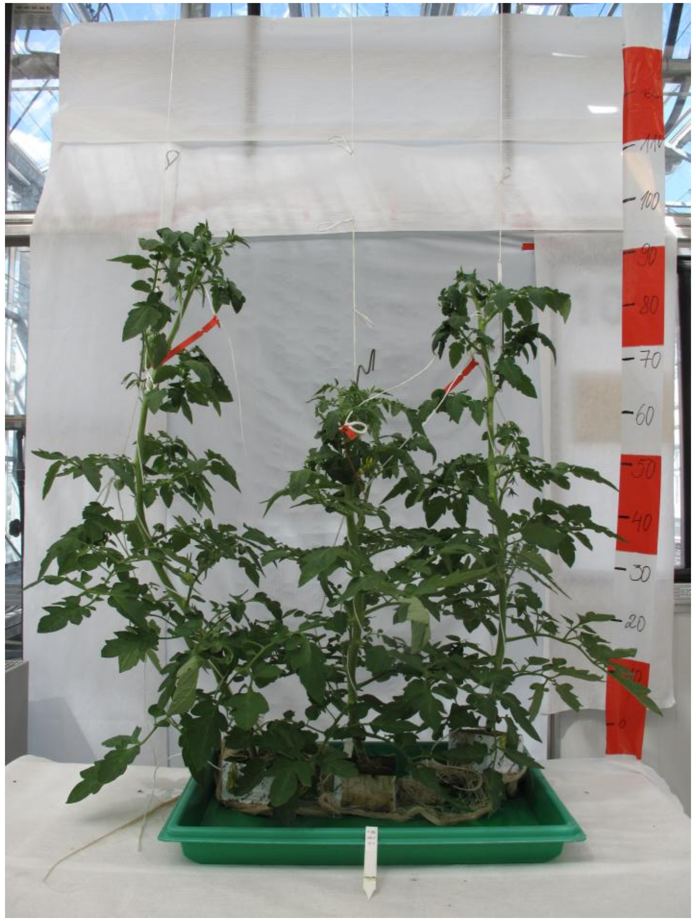

T10

control

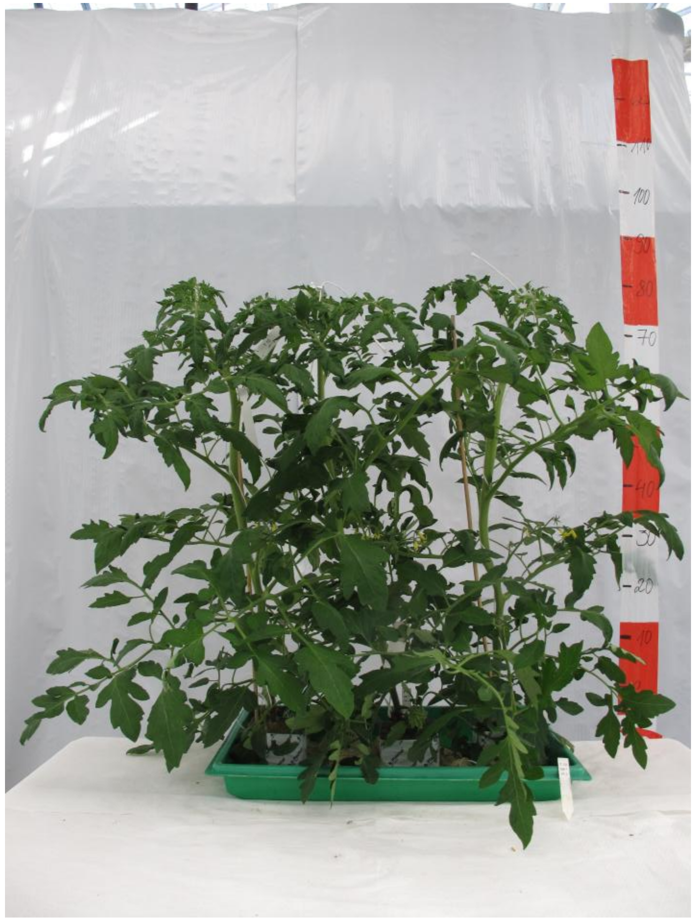

heat

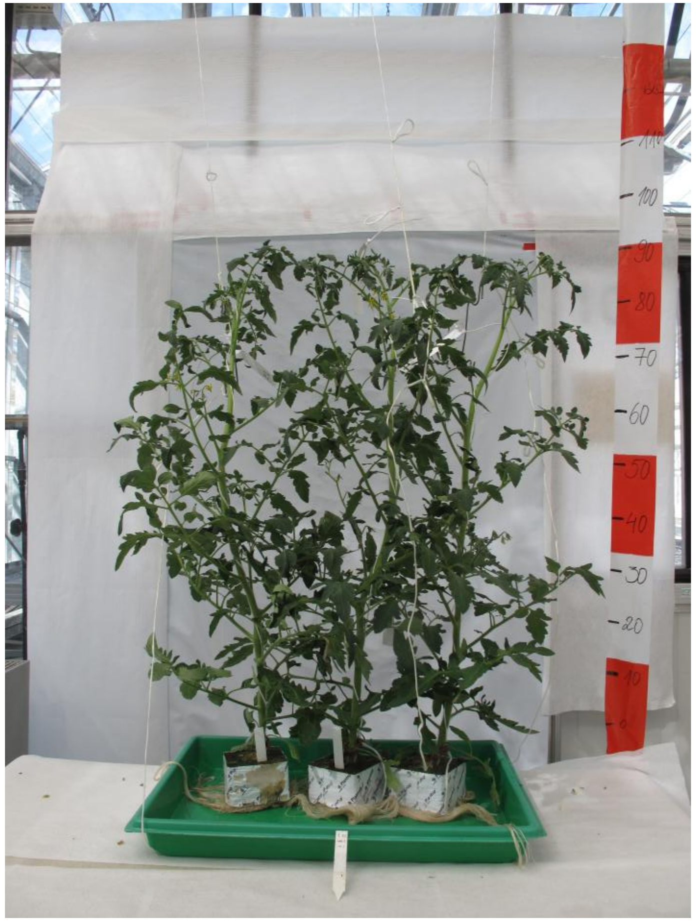

T11

control

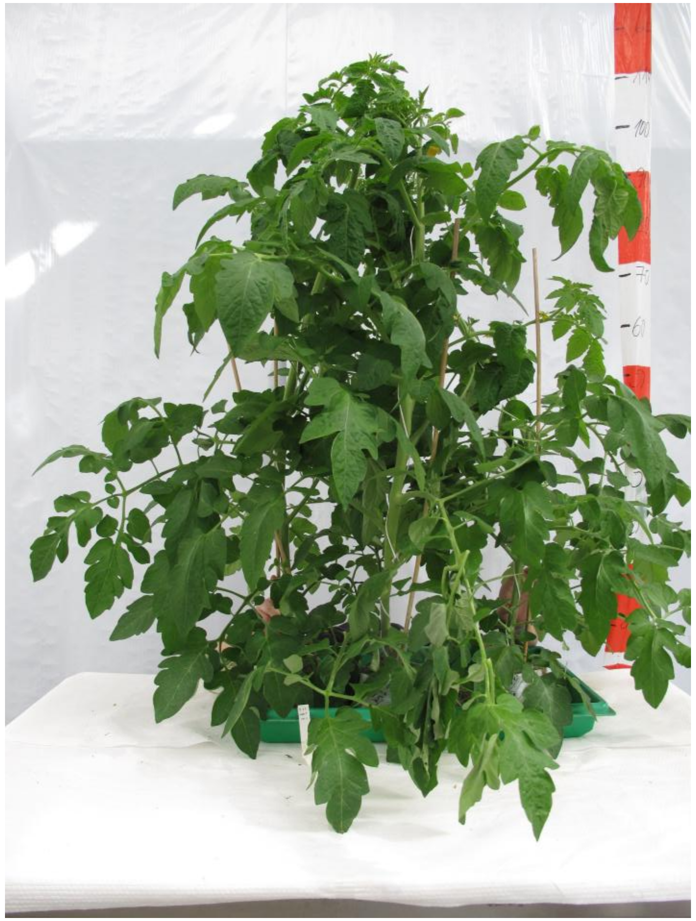

heat

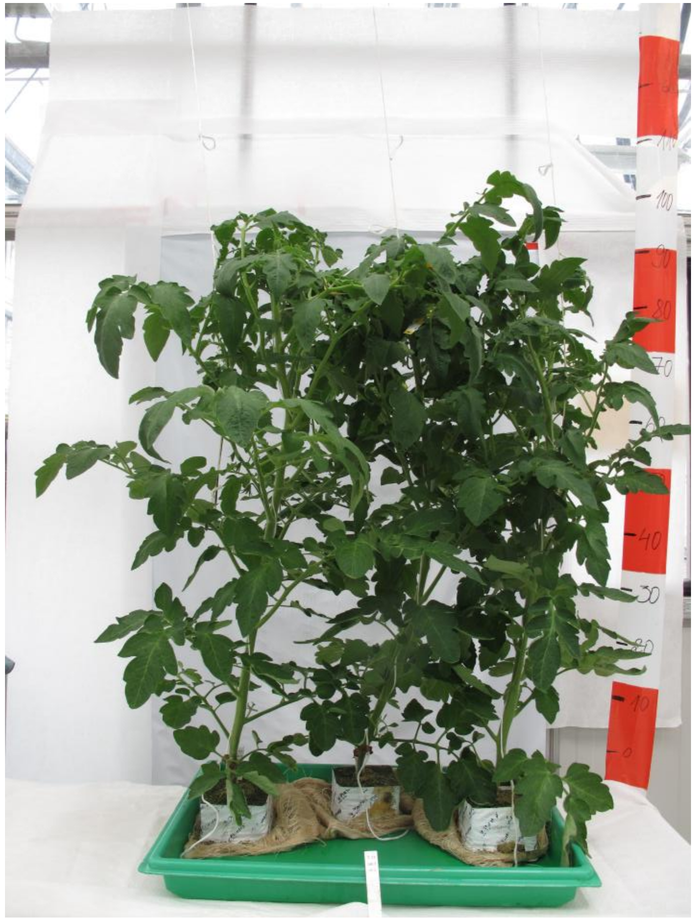

T12

control

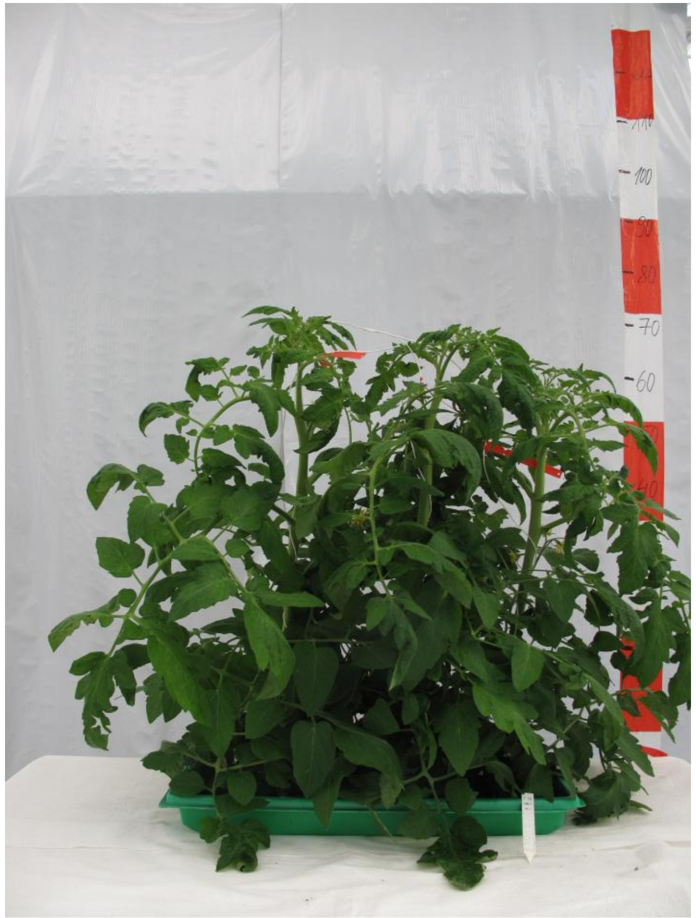

heat

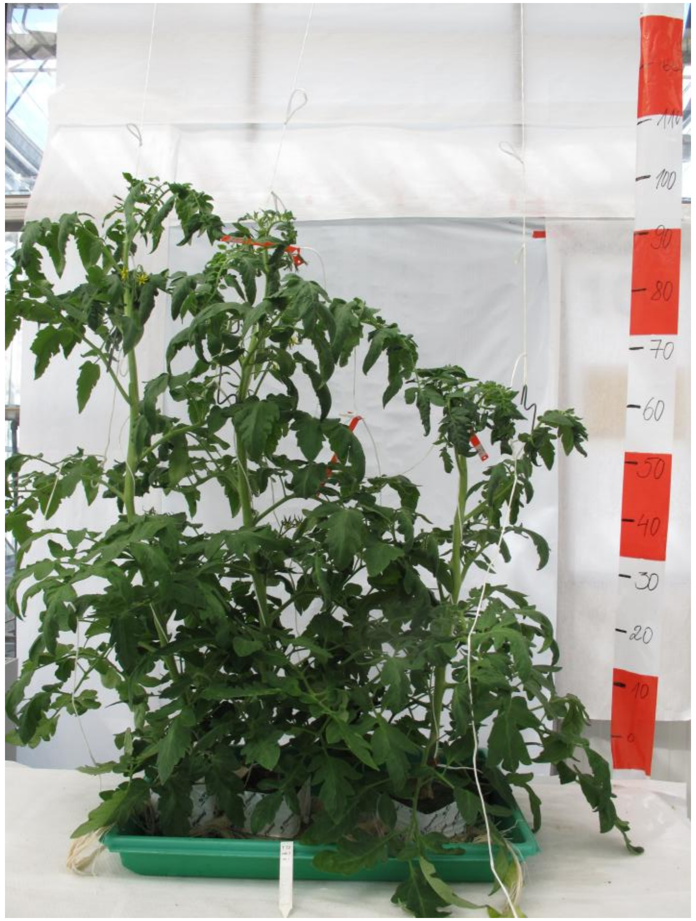

T13

control

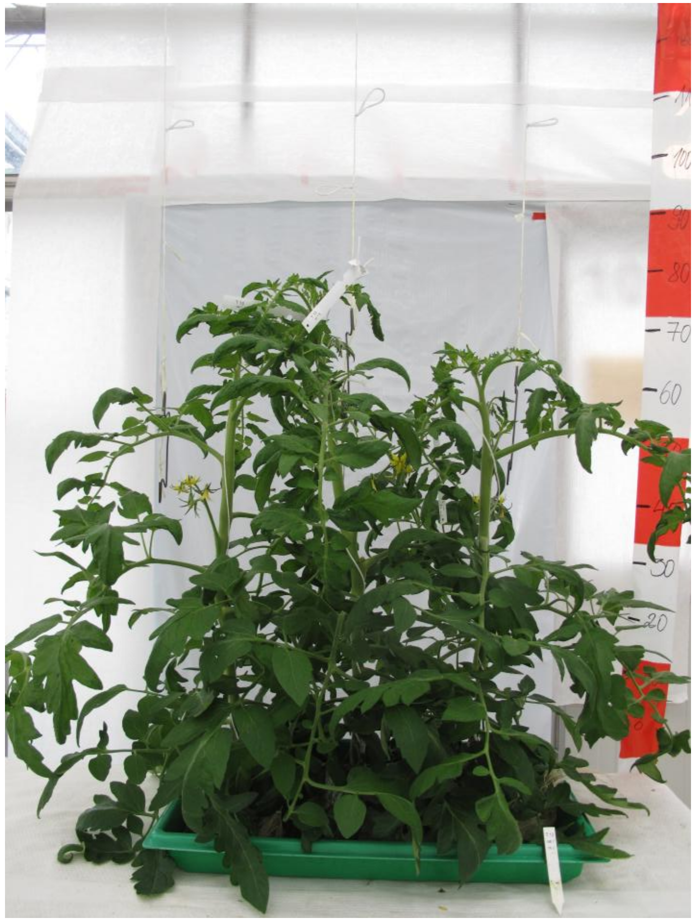

heat

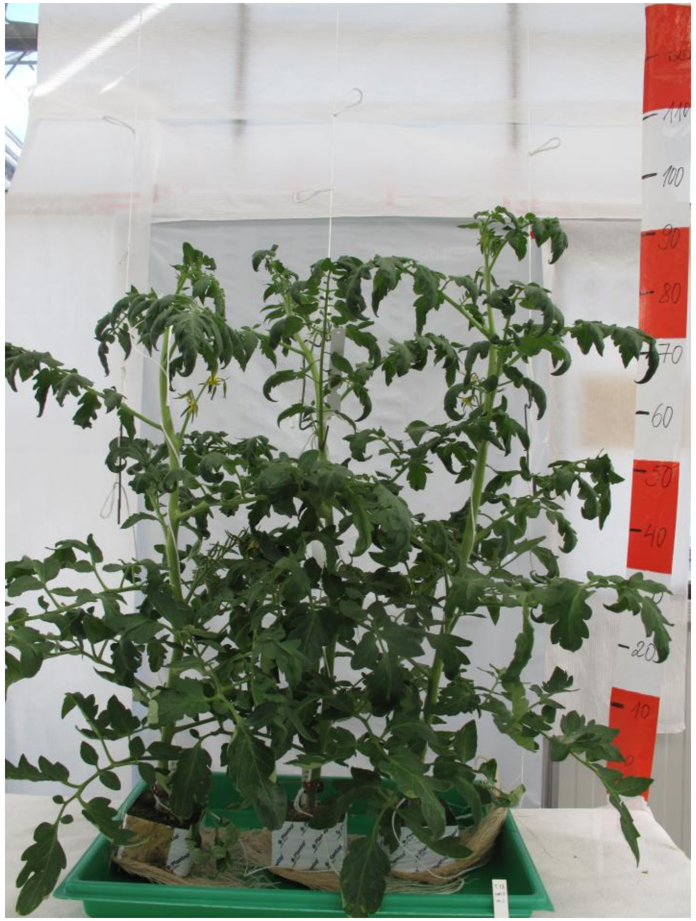

T14

control

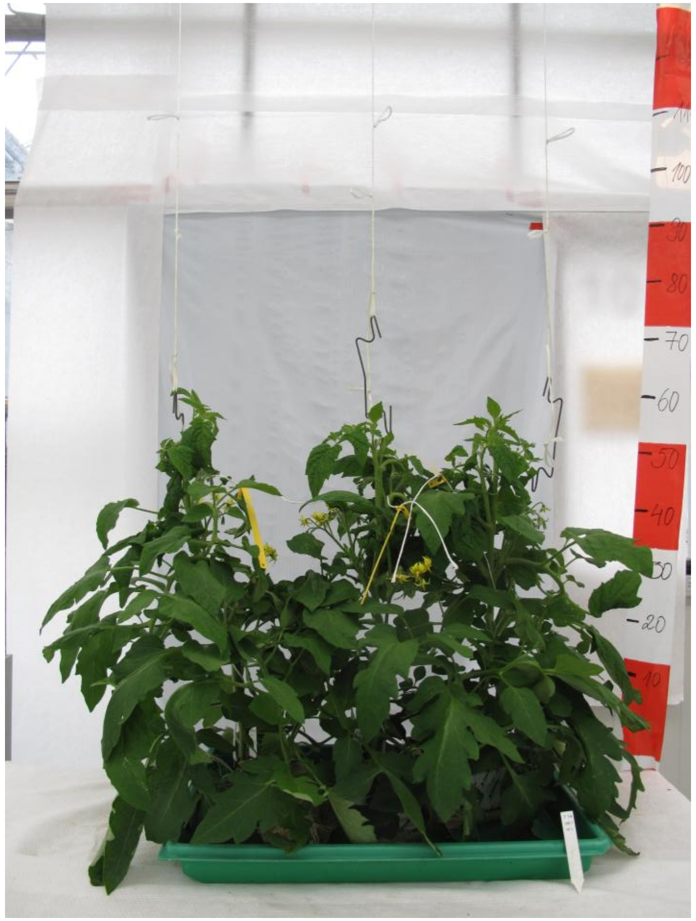

heat

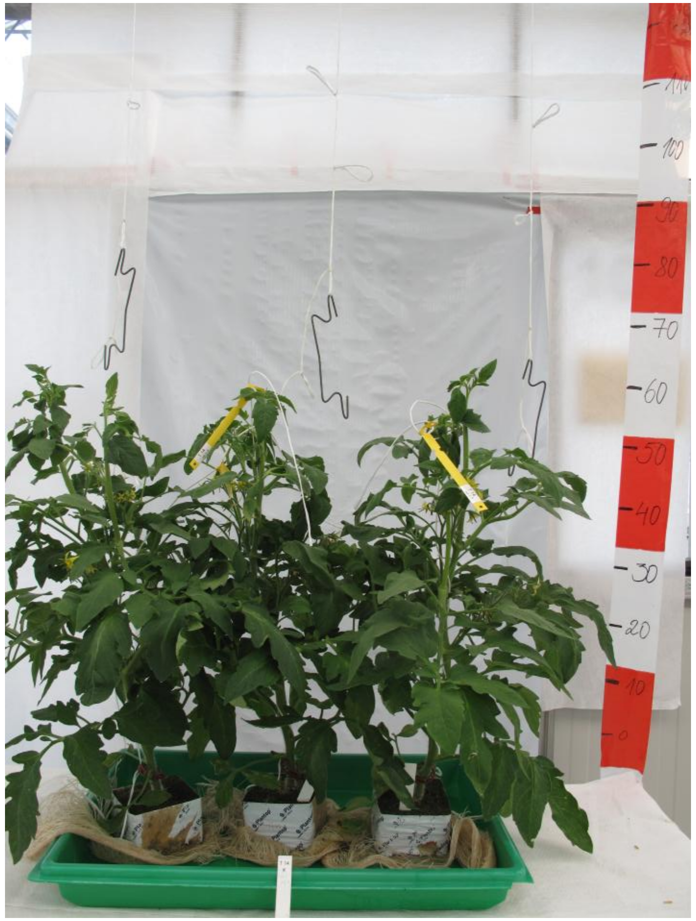

T15

control

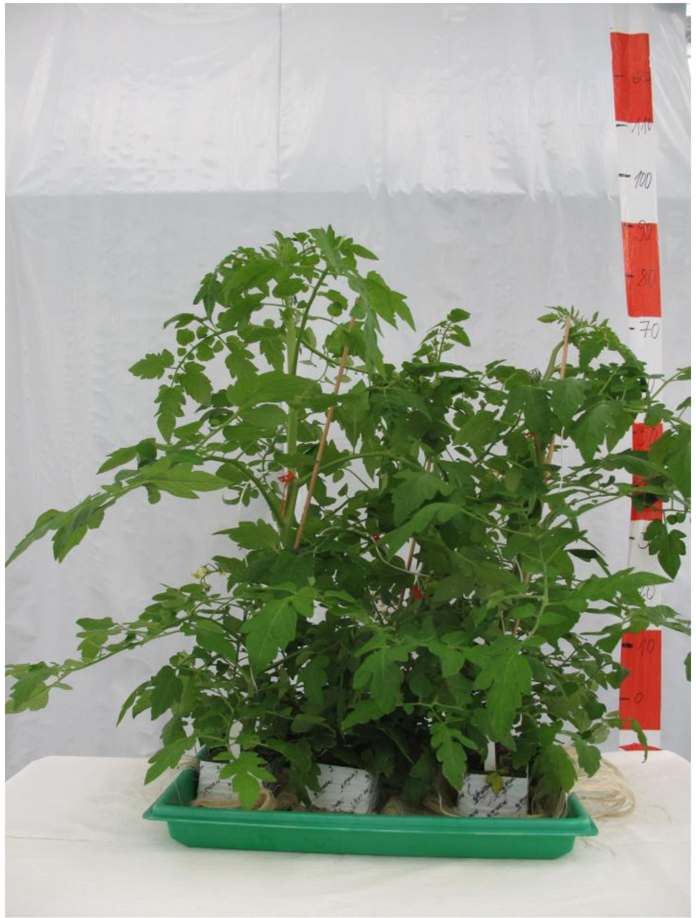

heat

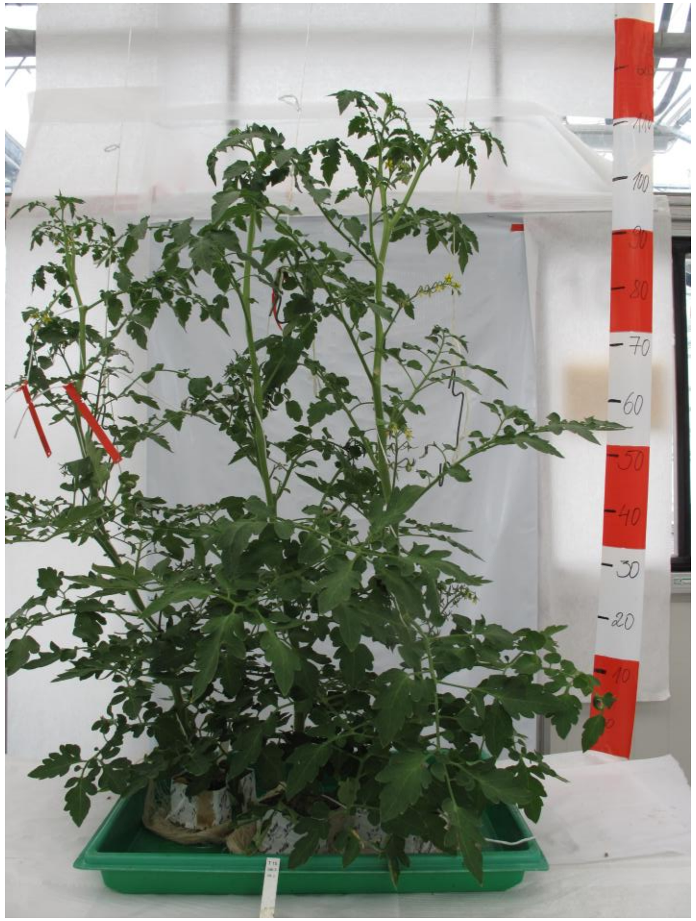

T16

control

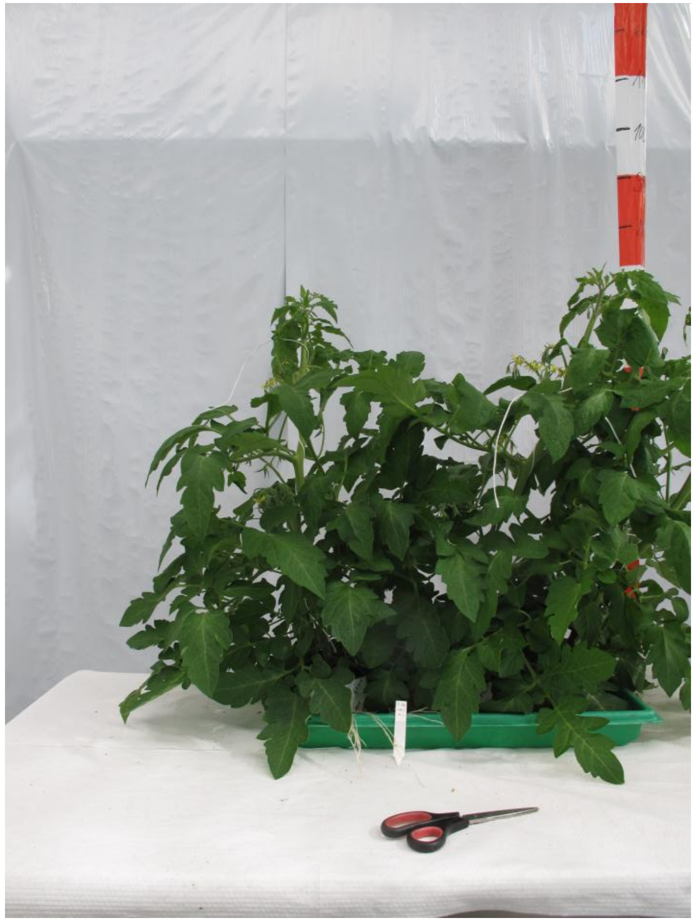

heat

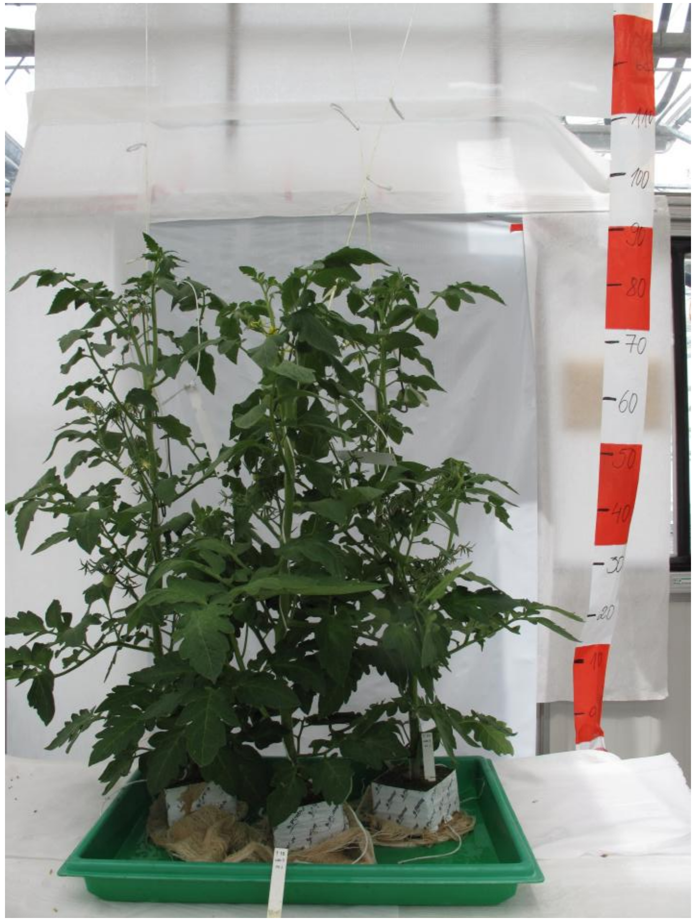

T17

control

heat

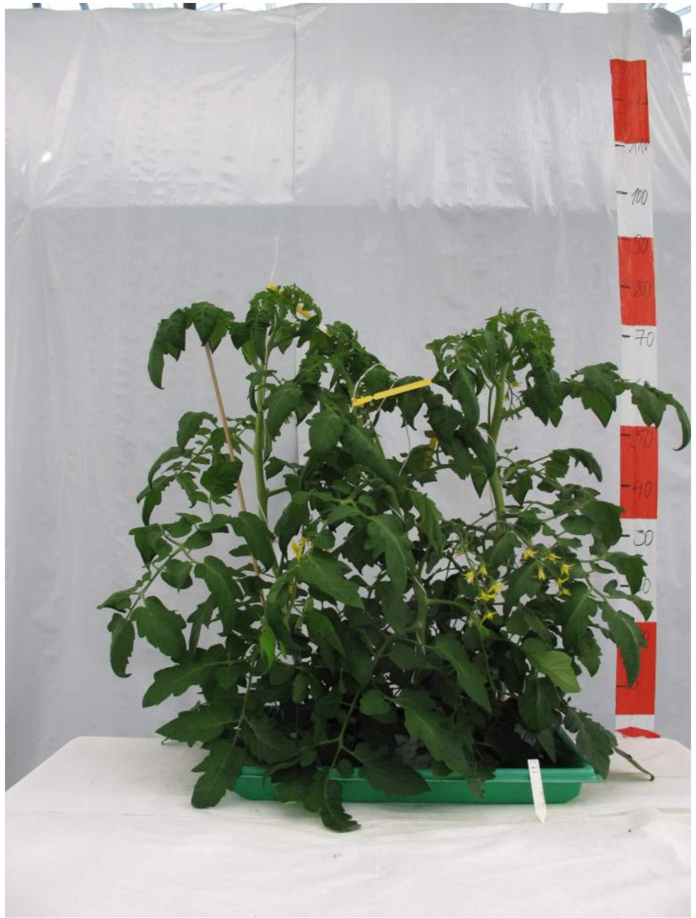

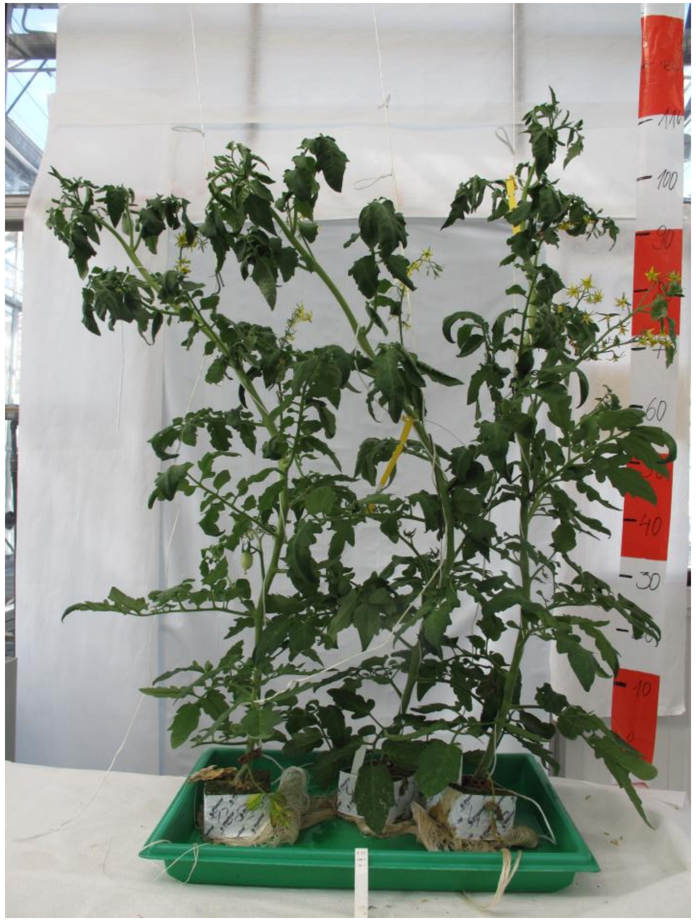

T18

control

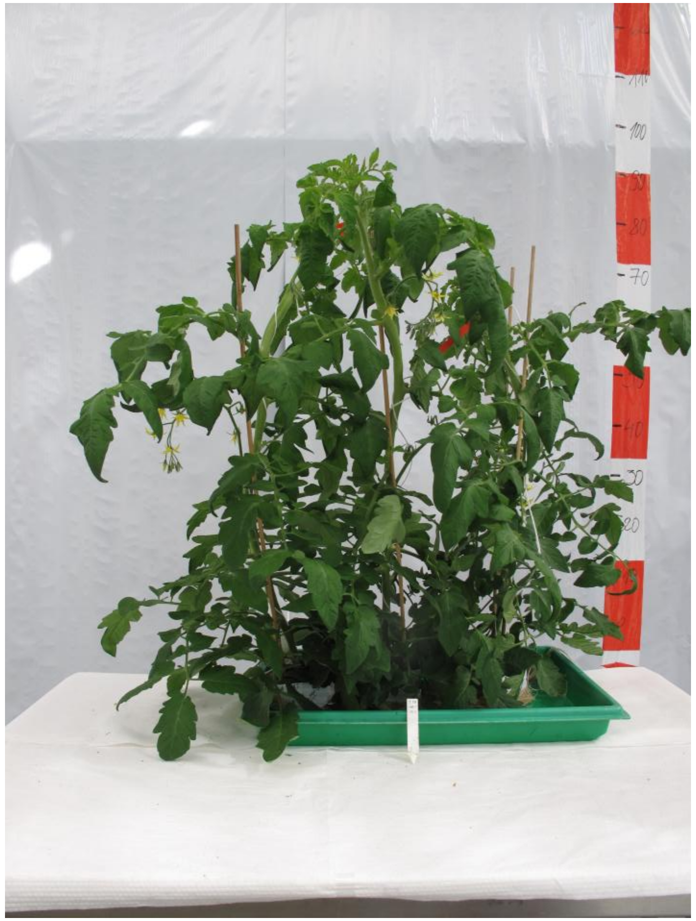

heat

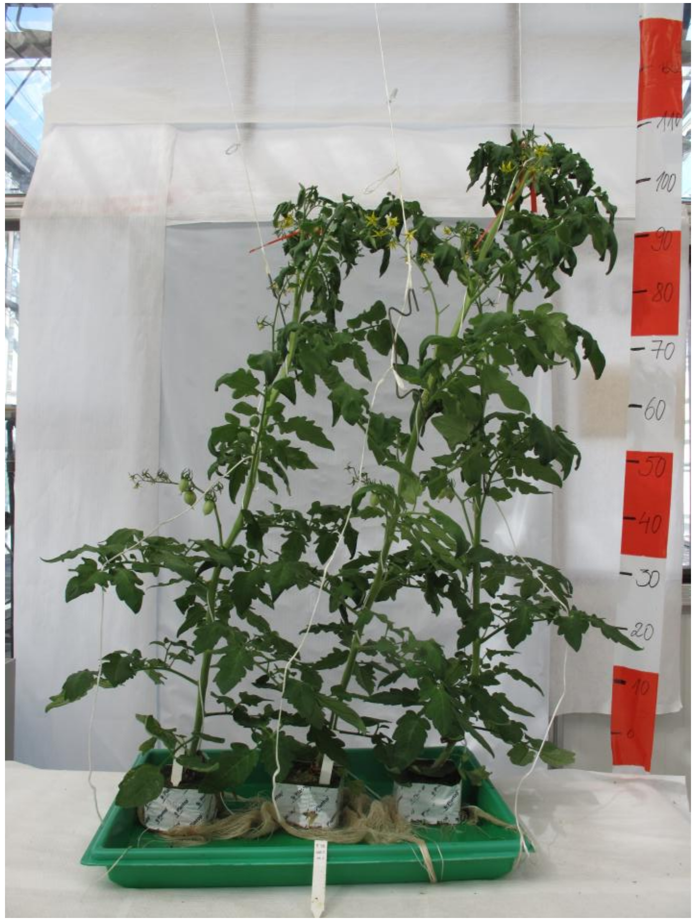

T19

control

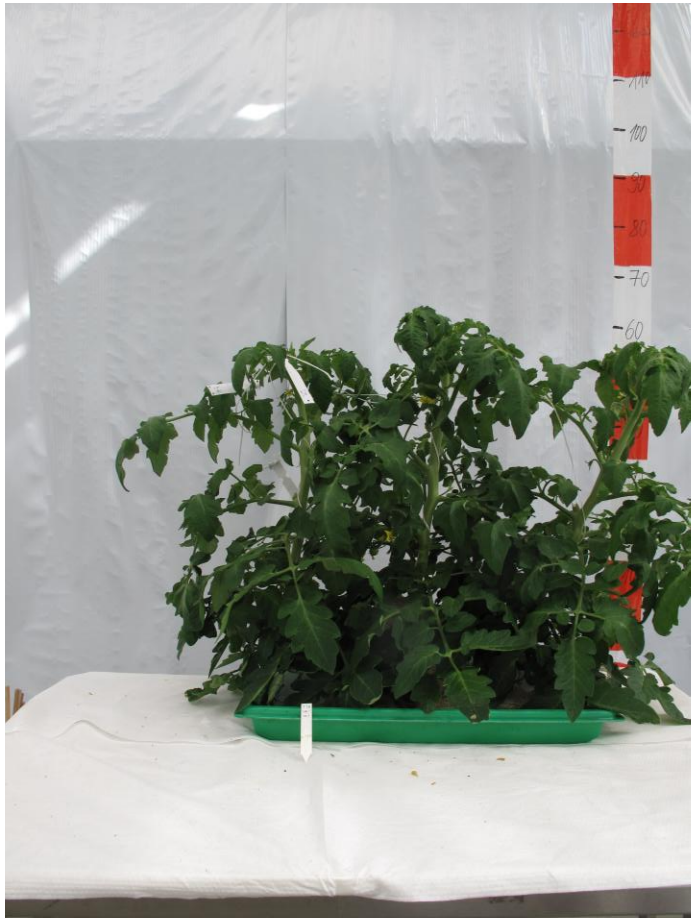

heat

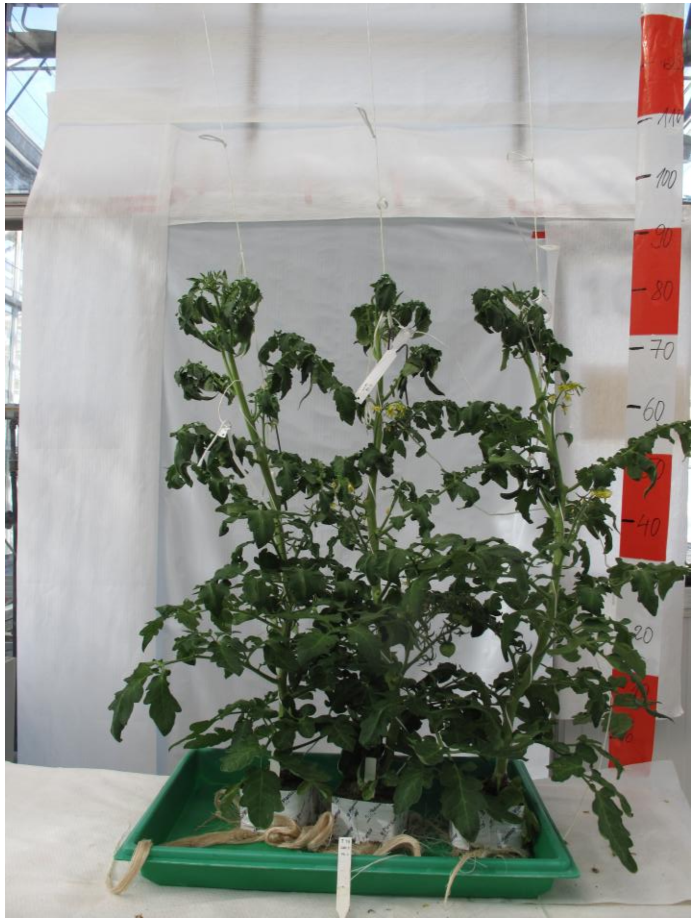

T20

control

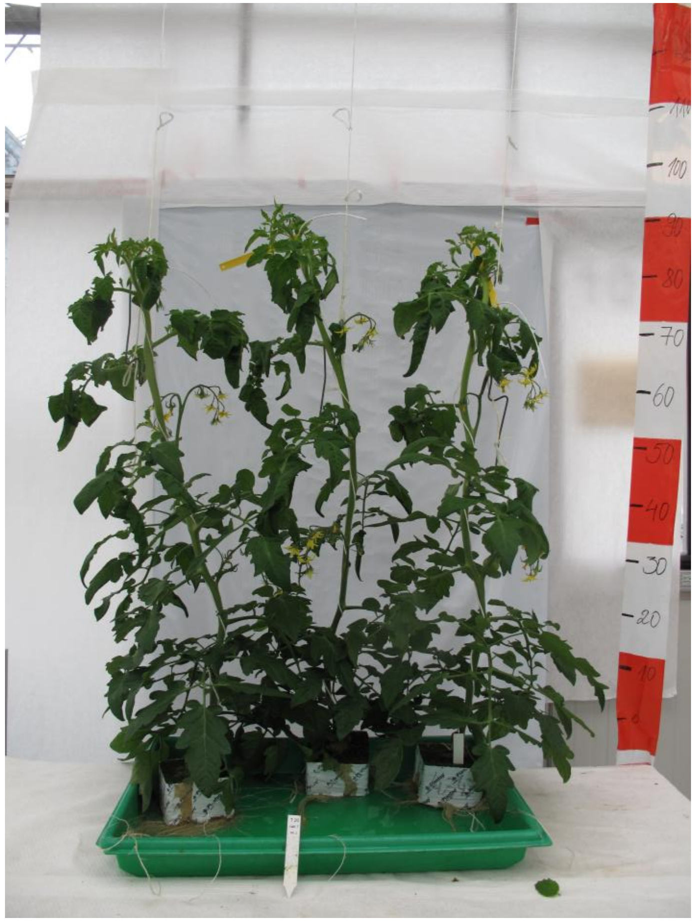

heat

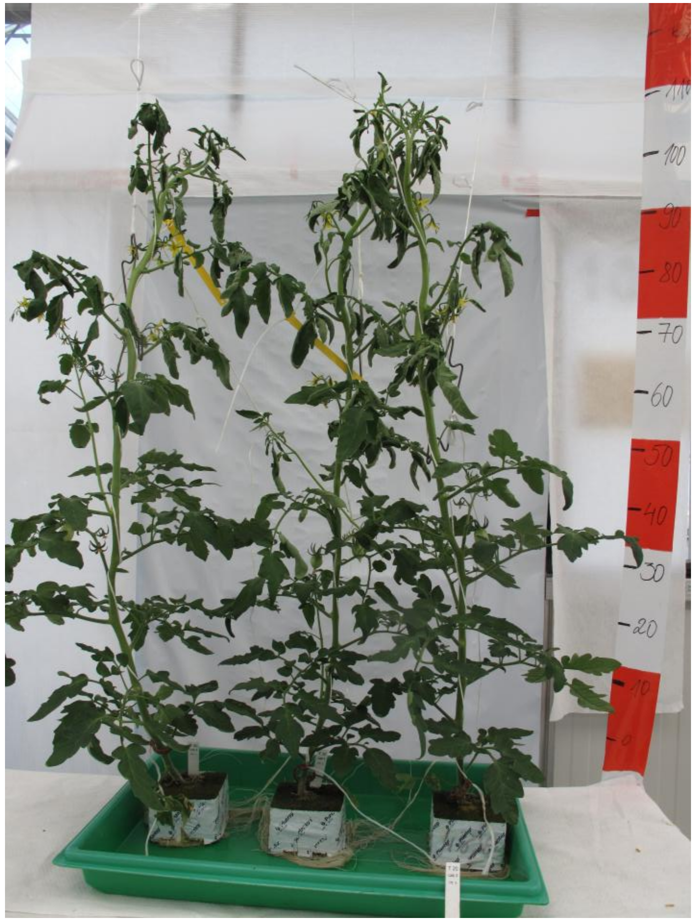

T21

control

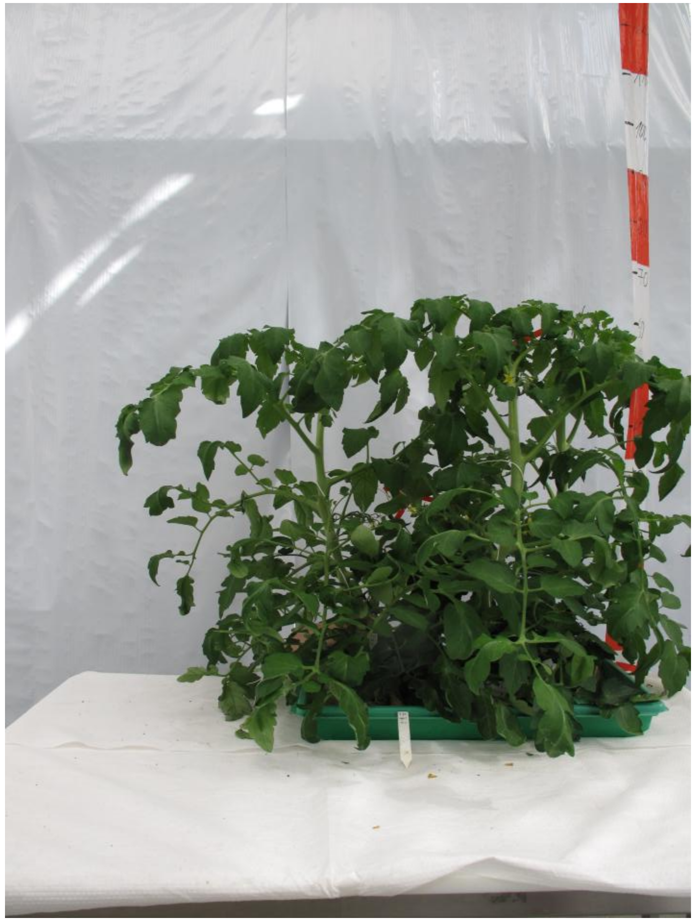

heat

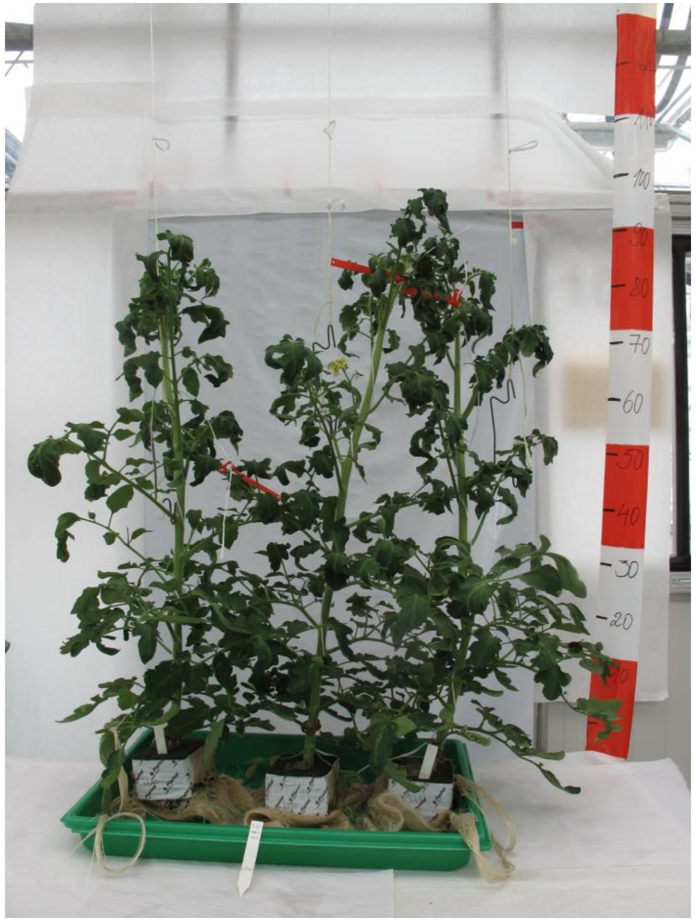

T22

control

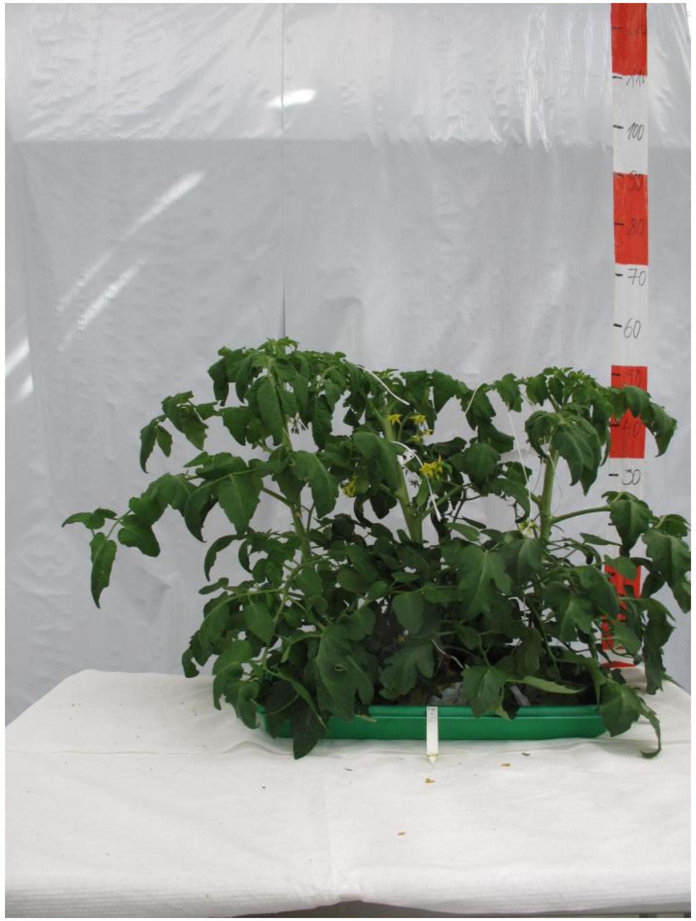

heat

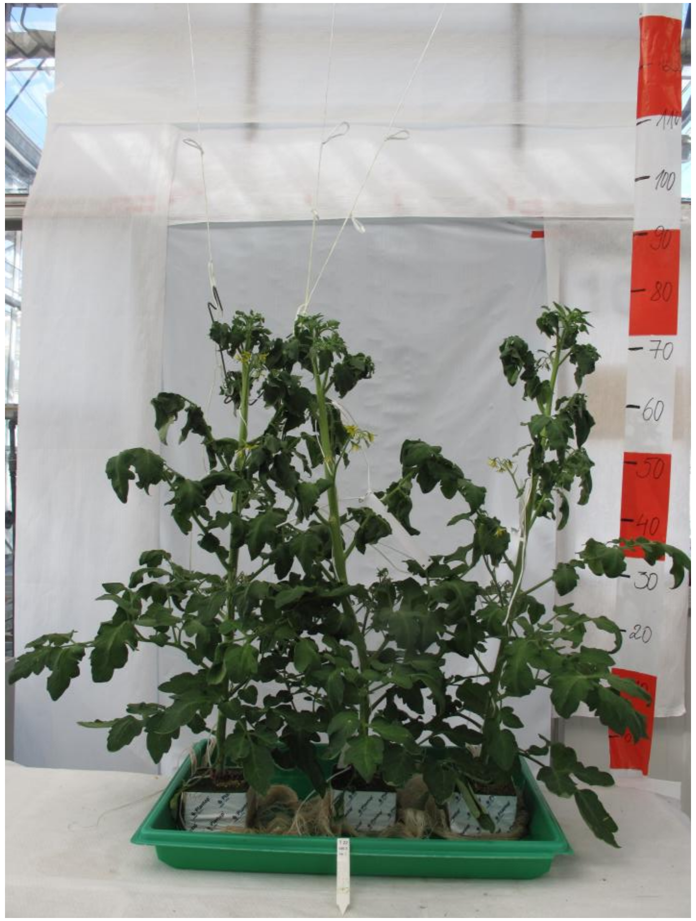

T23

control

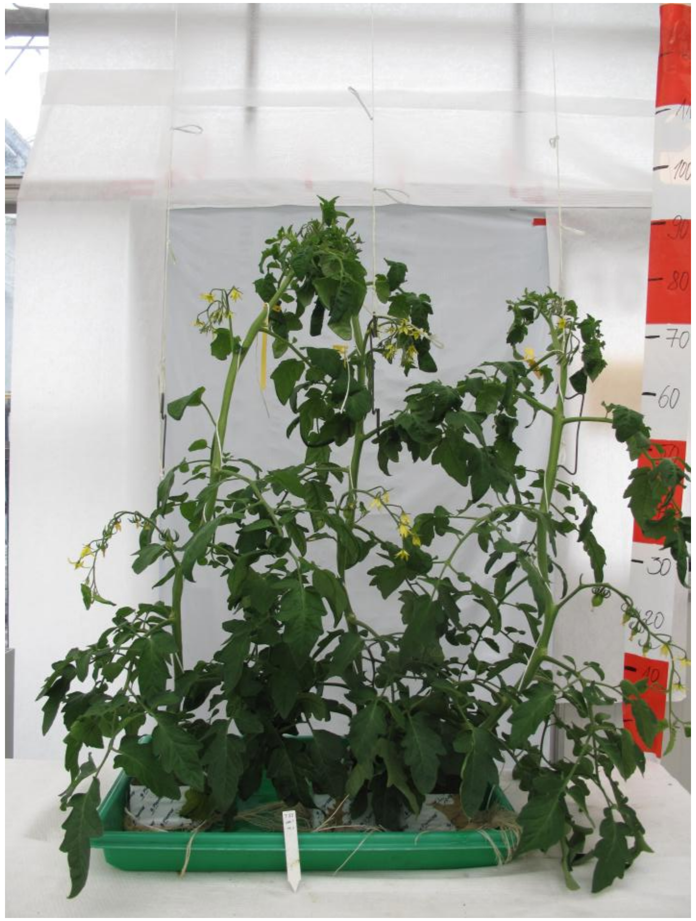

heat

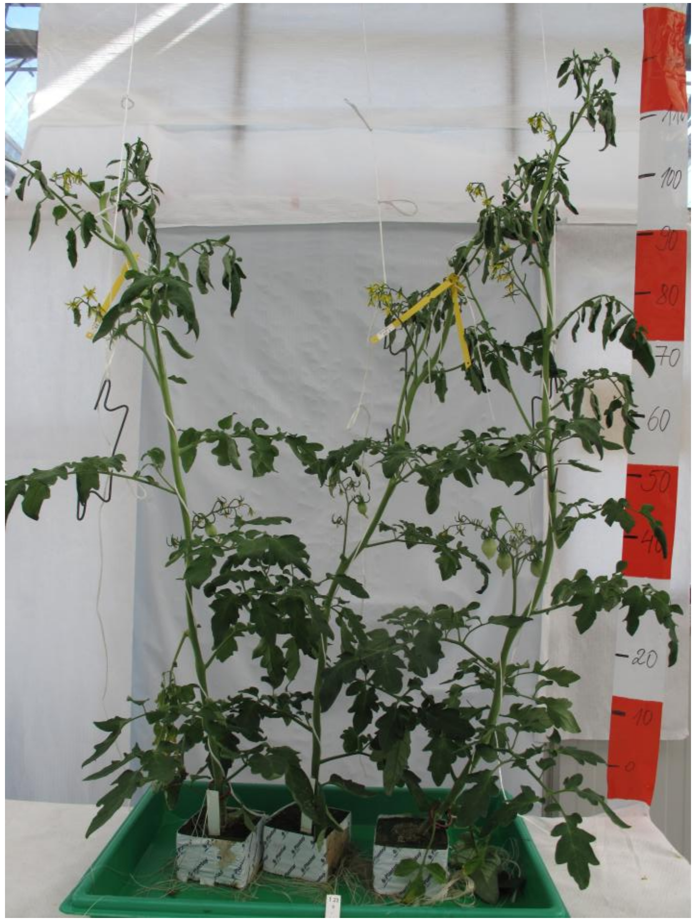

T24

control

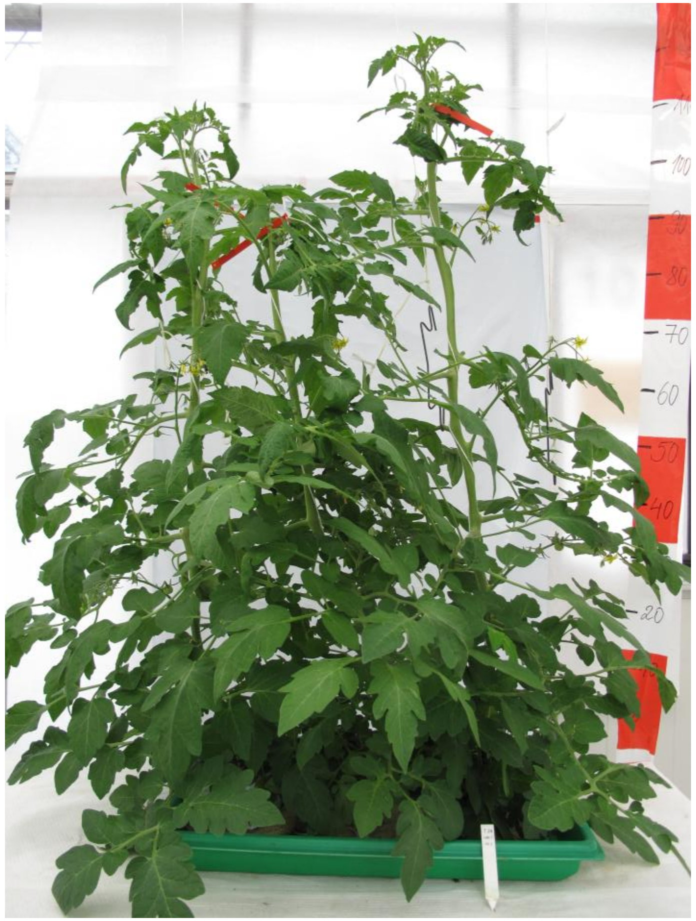

heat

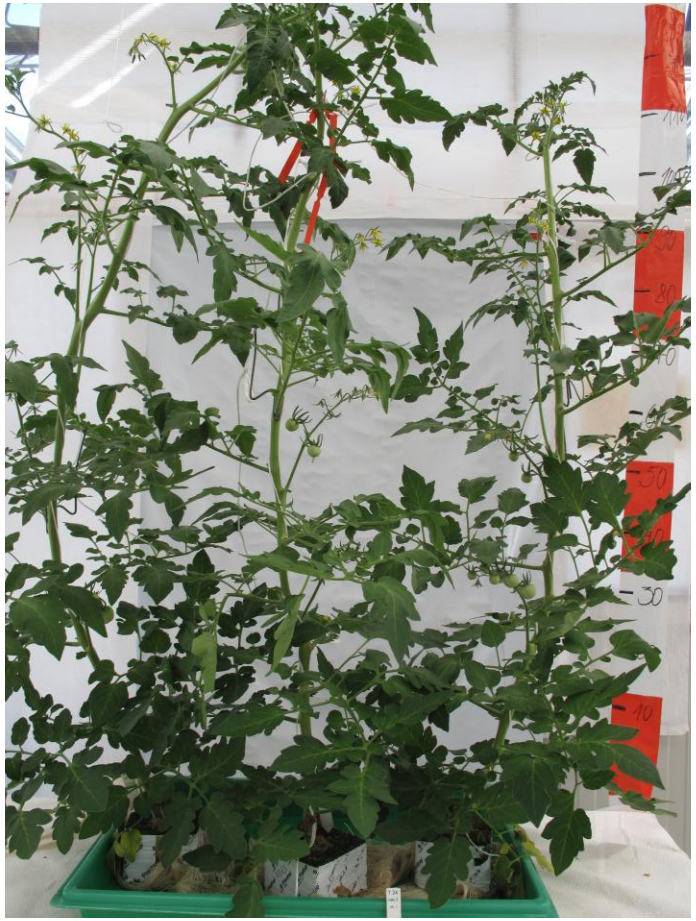

T25

control

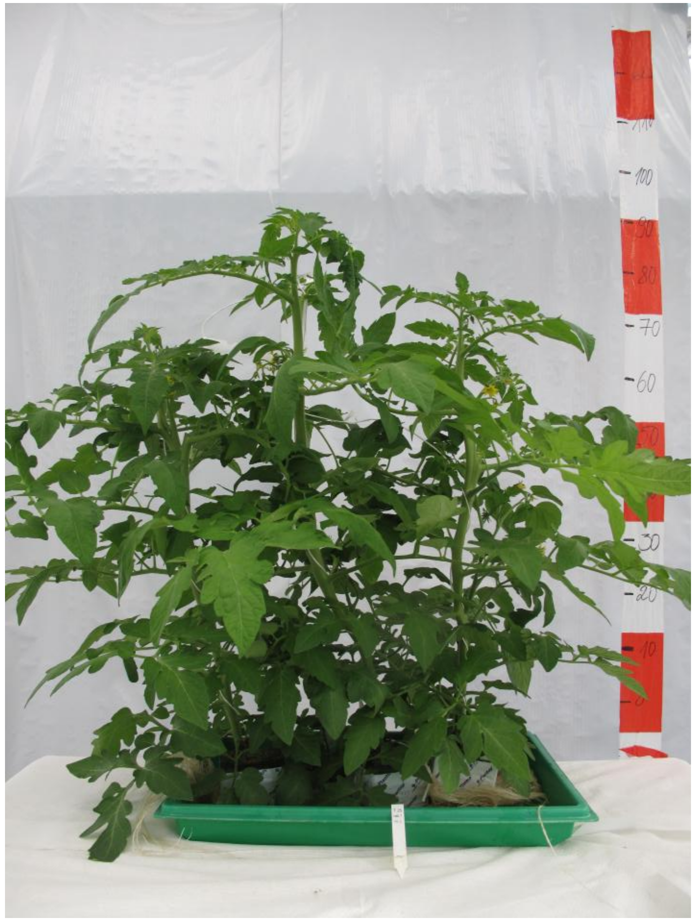

heat

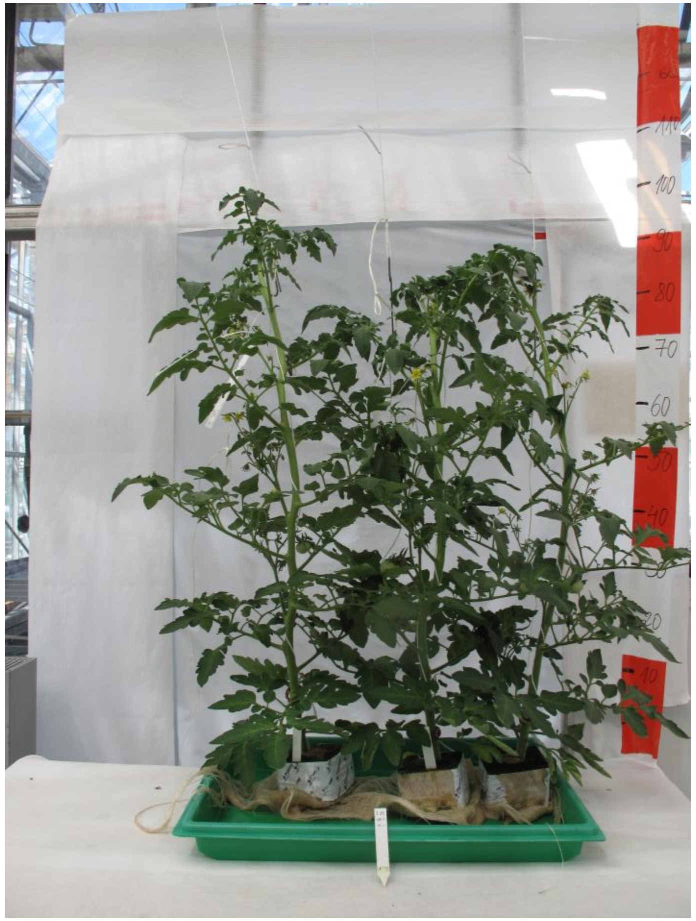

T26

control

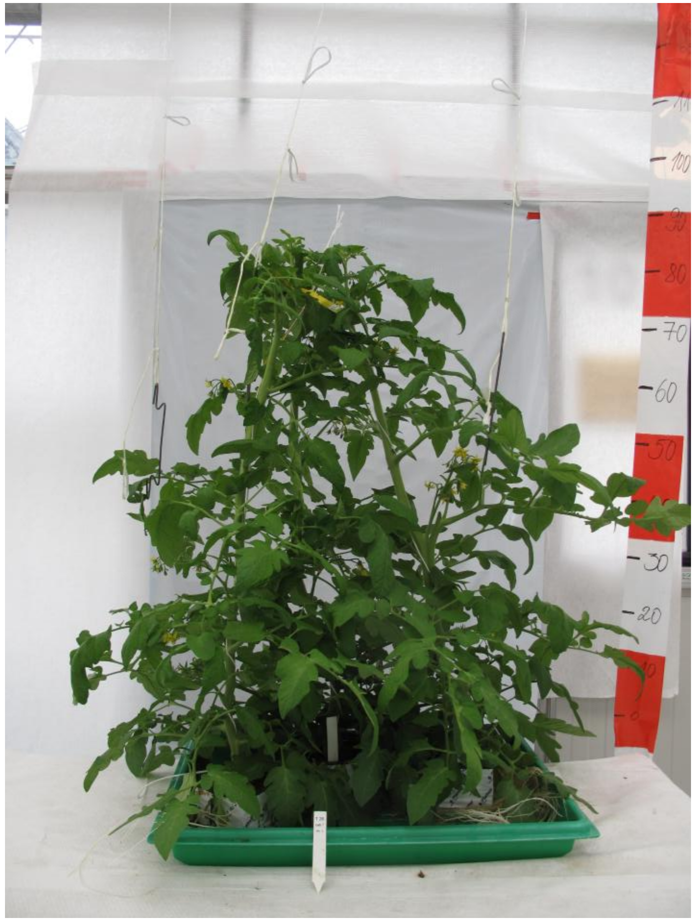

heat

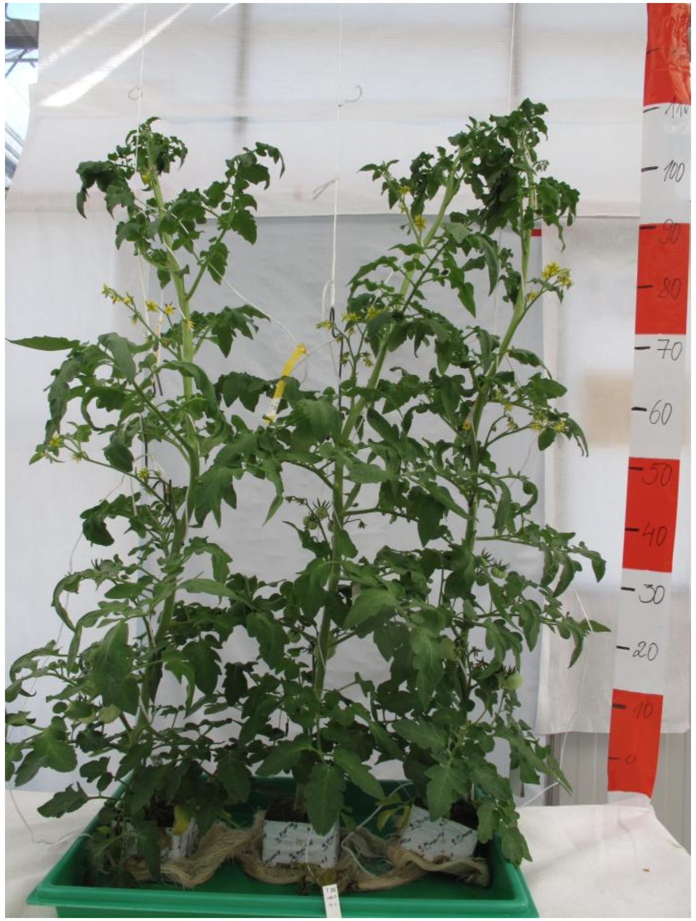

T27

control

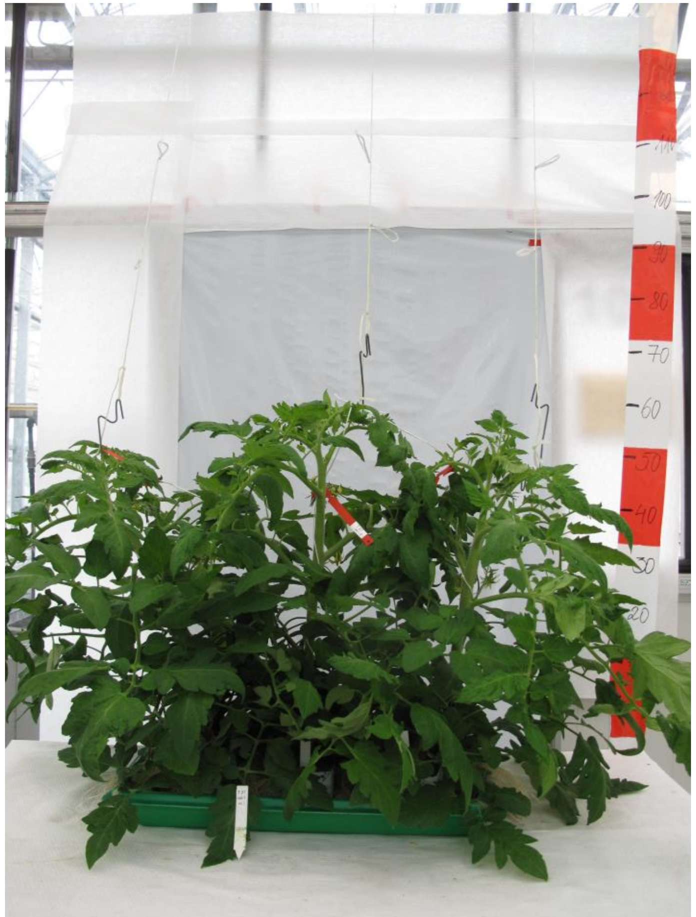

heat

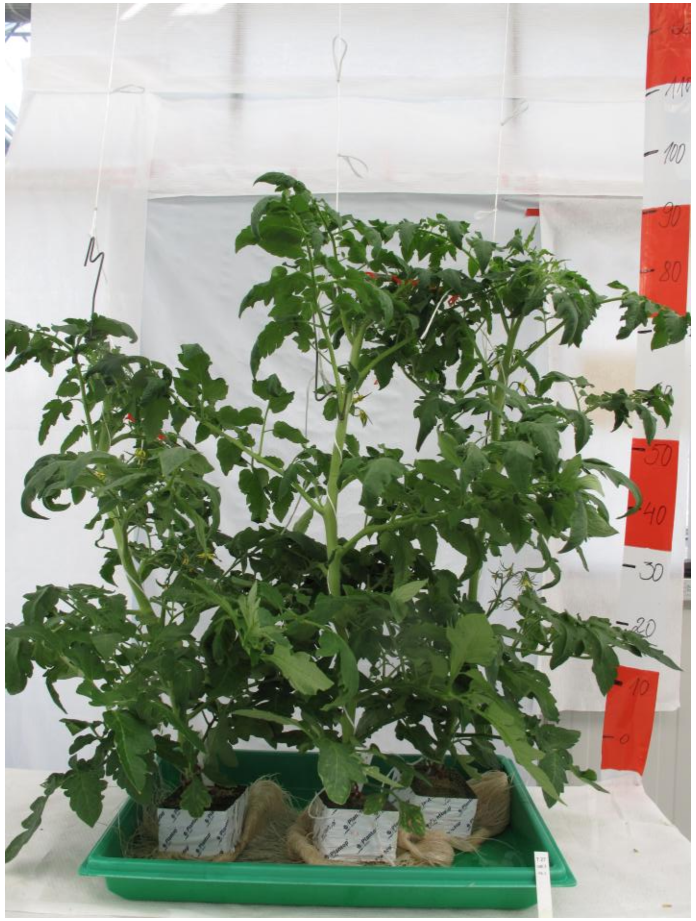

T28

control

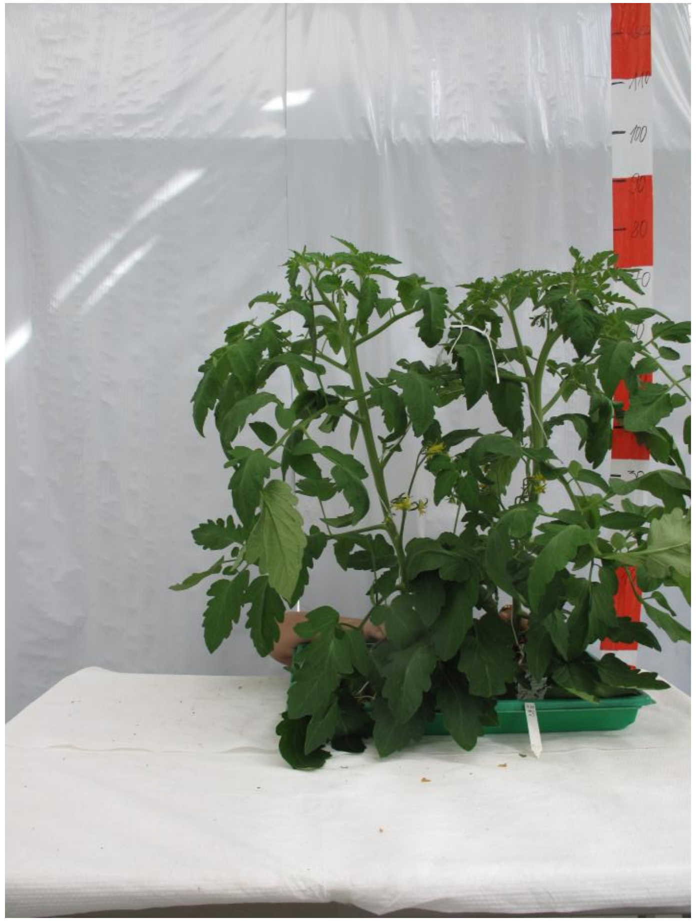

heat

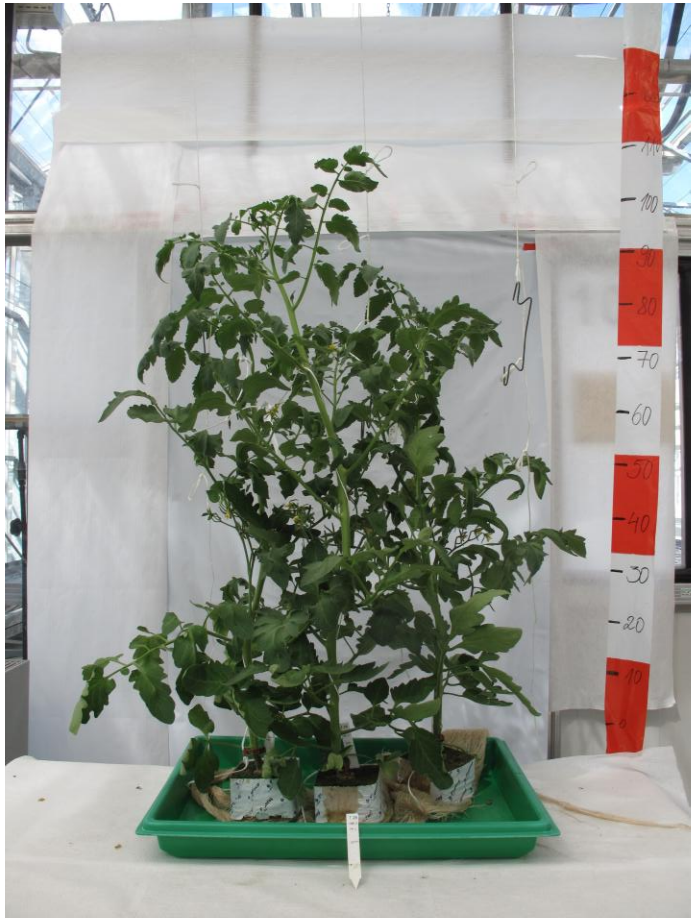

T29

control

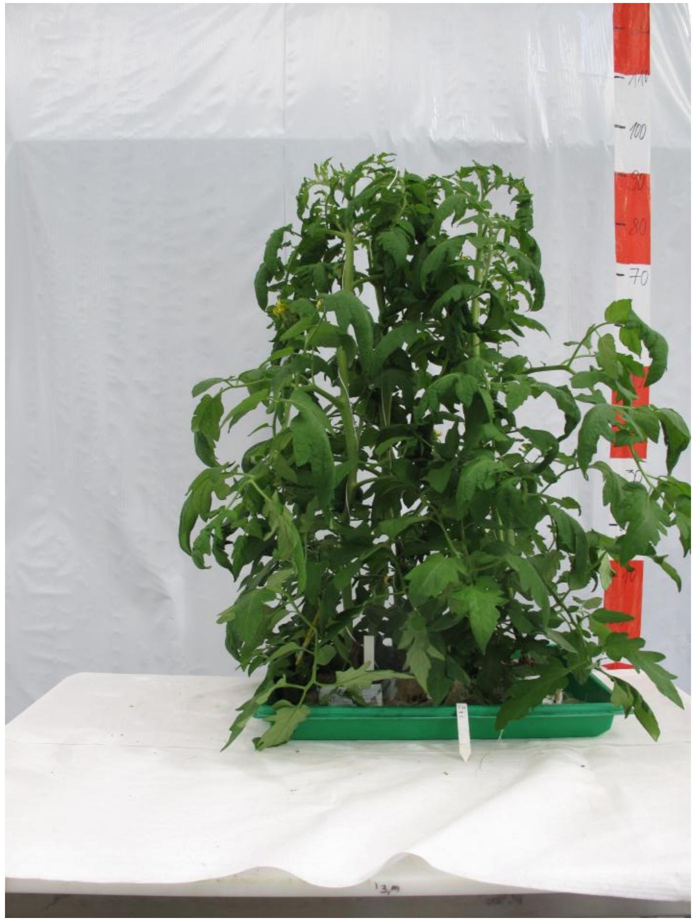

heat

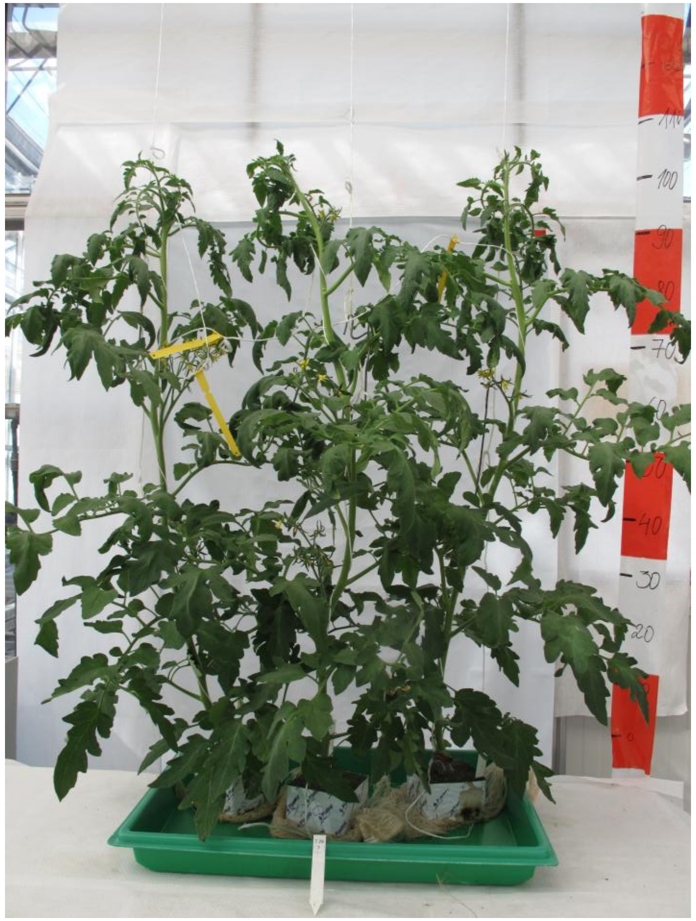

T30

control

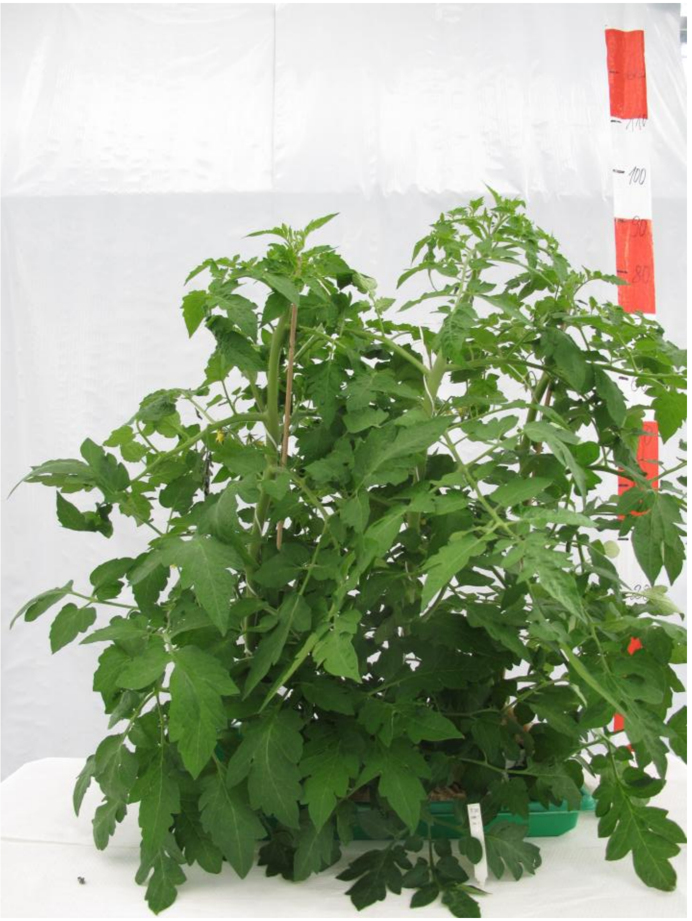

heat

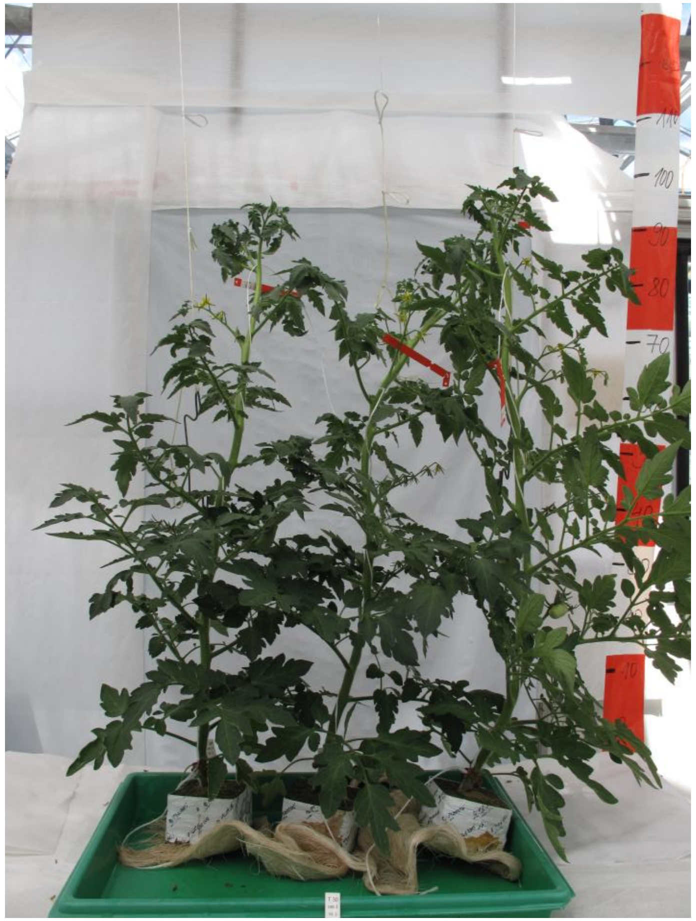

T31

control

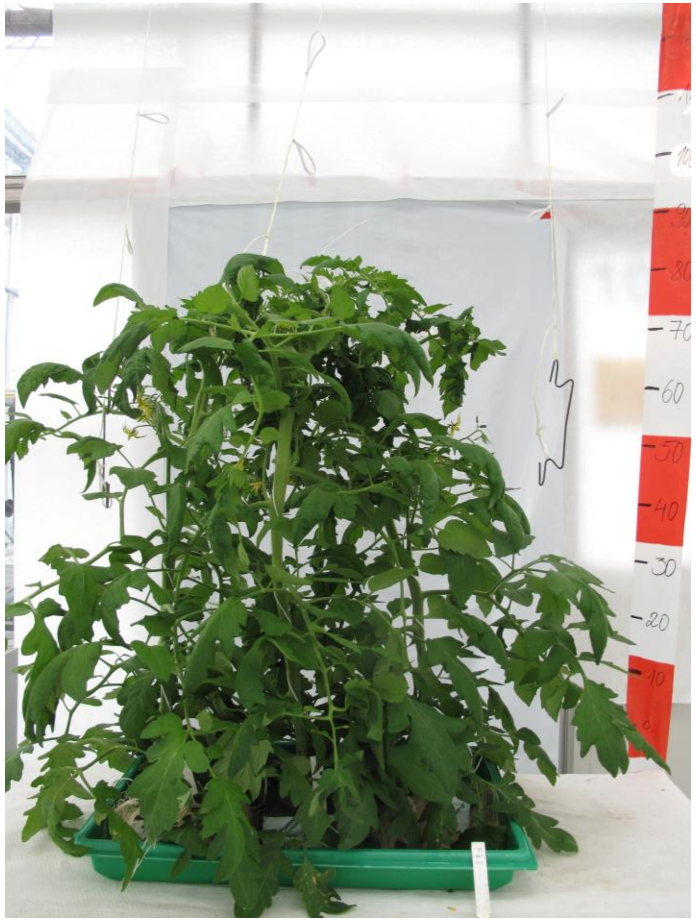

heat

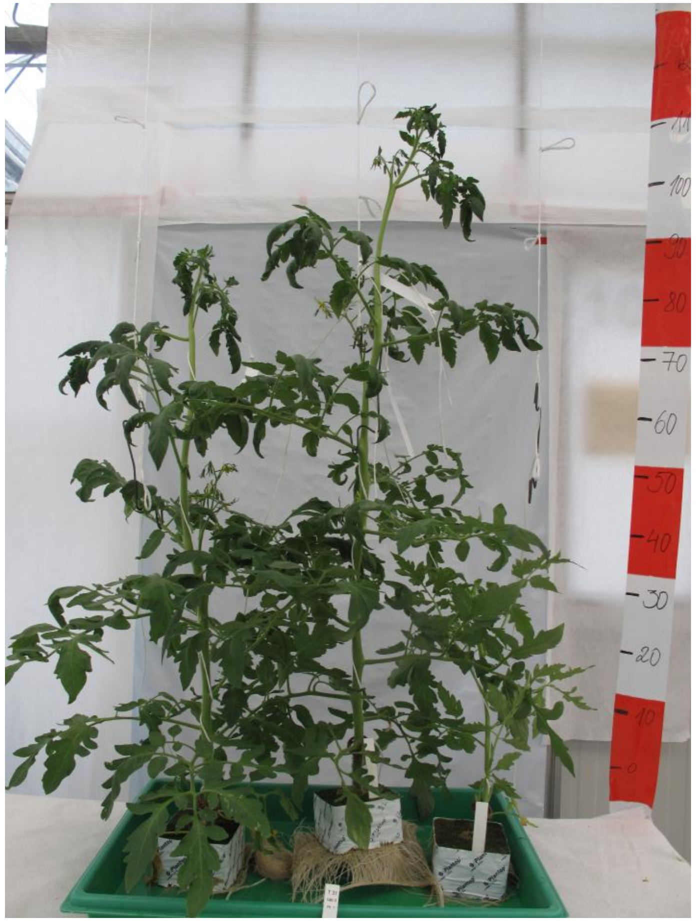

T32

control

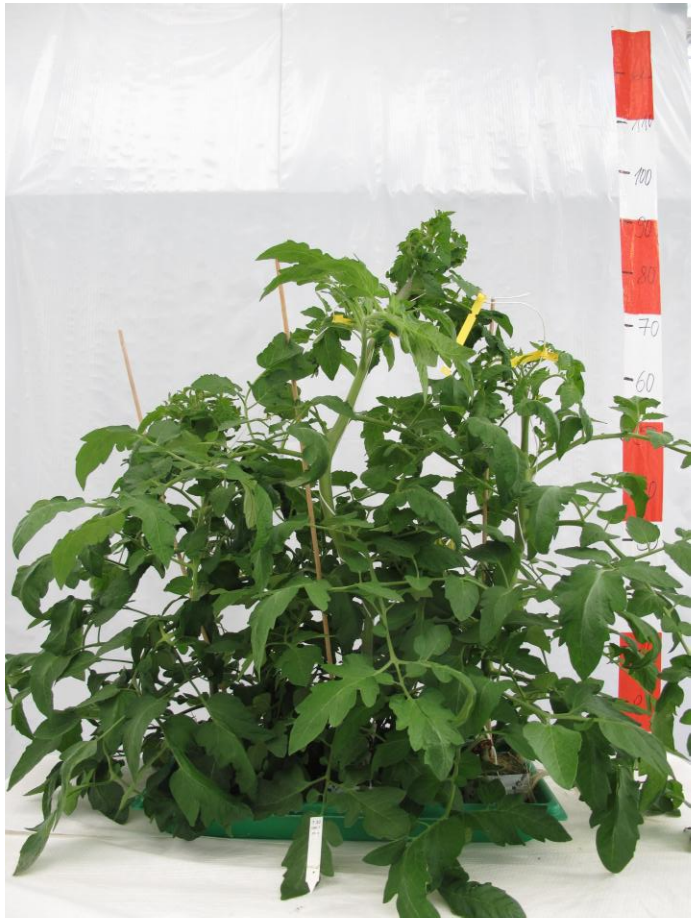

heat

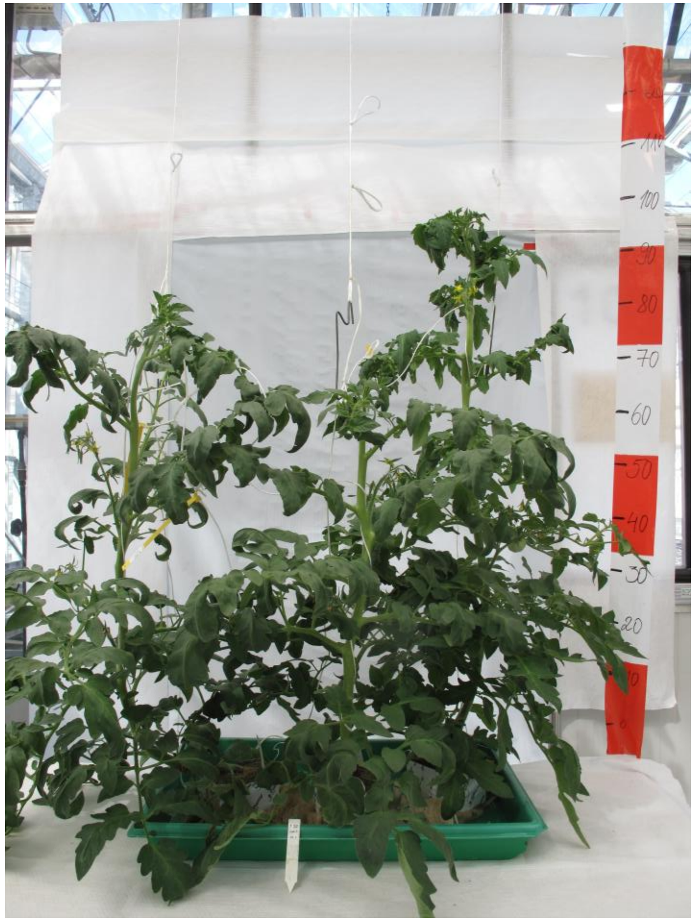

T33

control

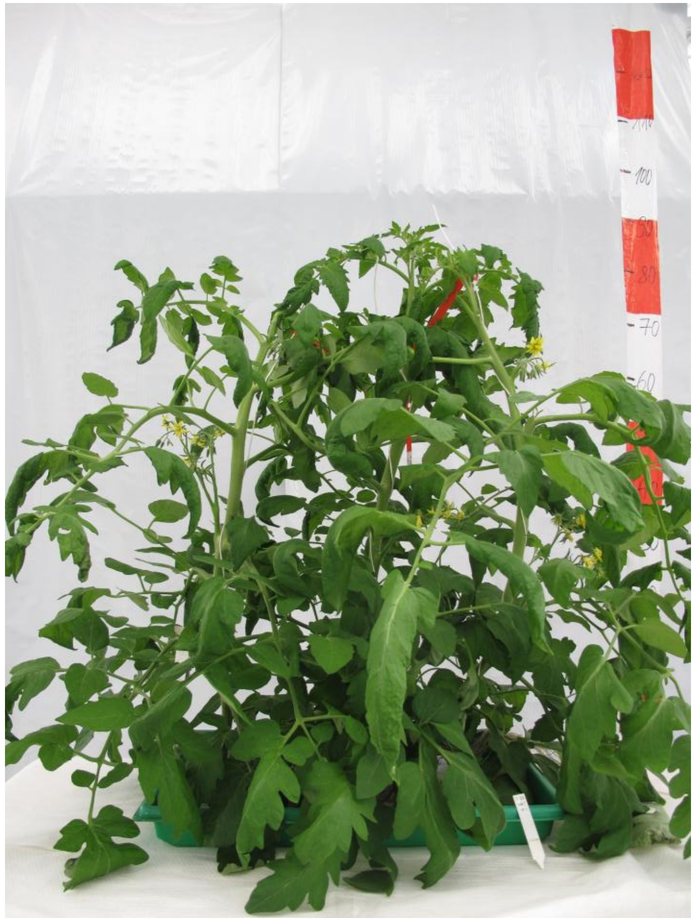

heat

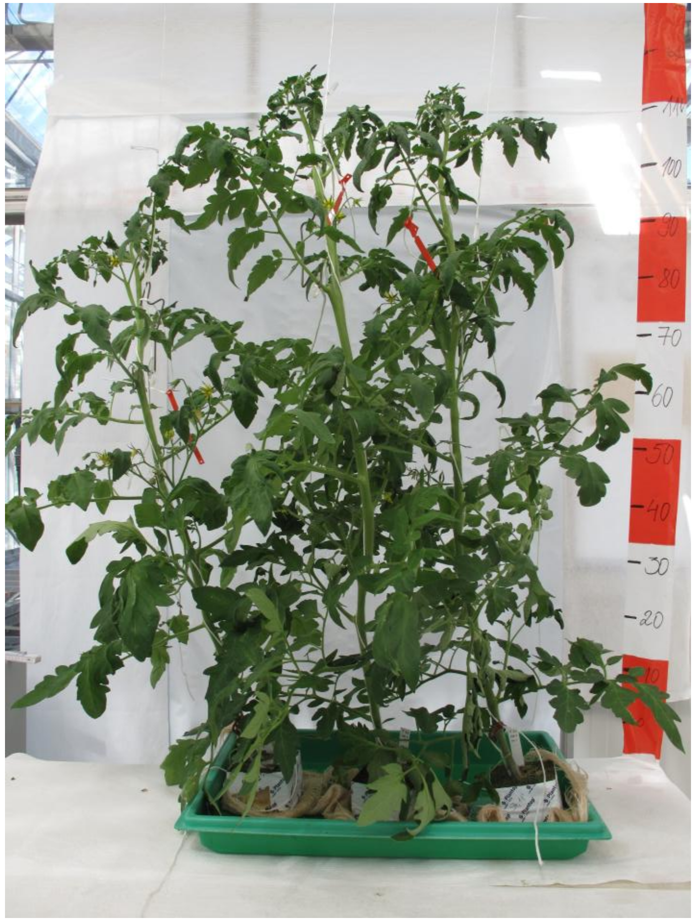

T34

control

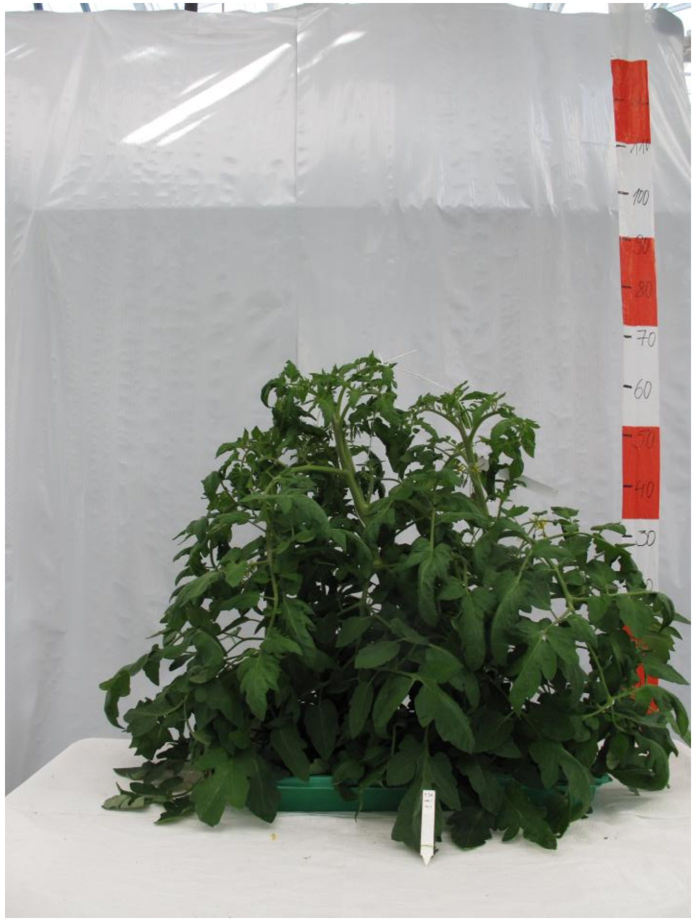

heat

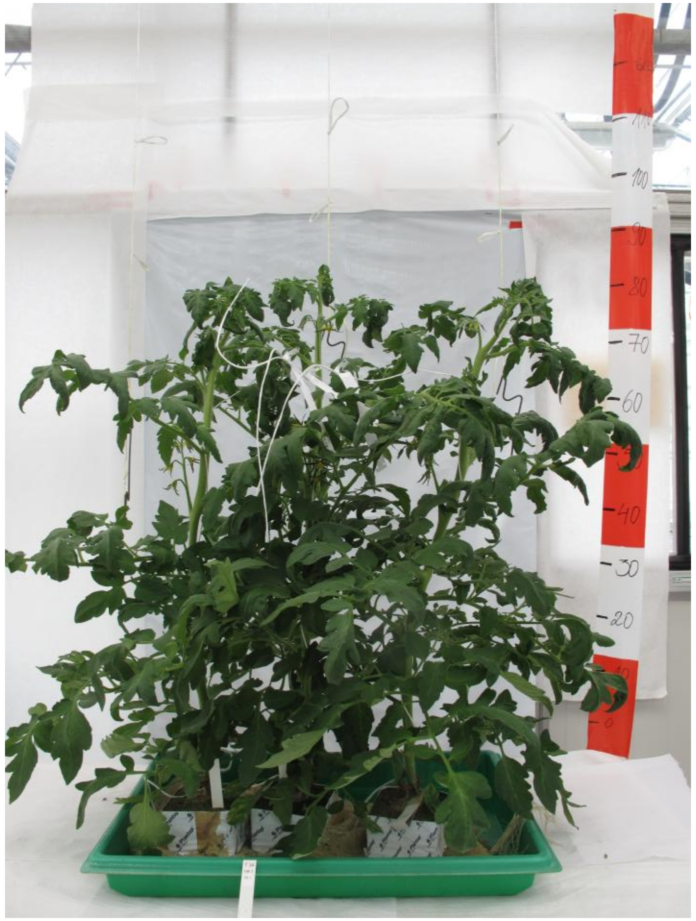

T35

control

heat

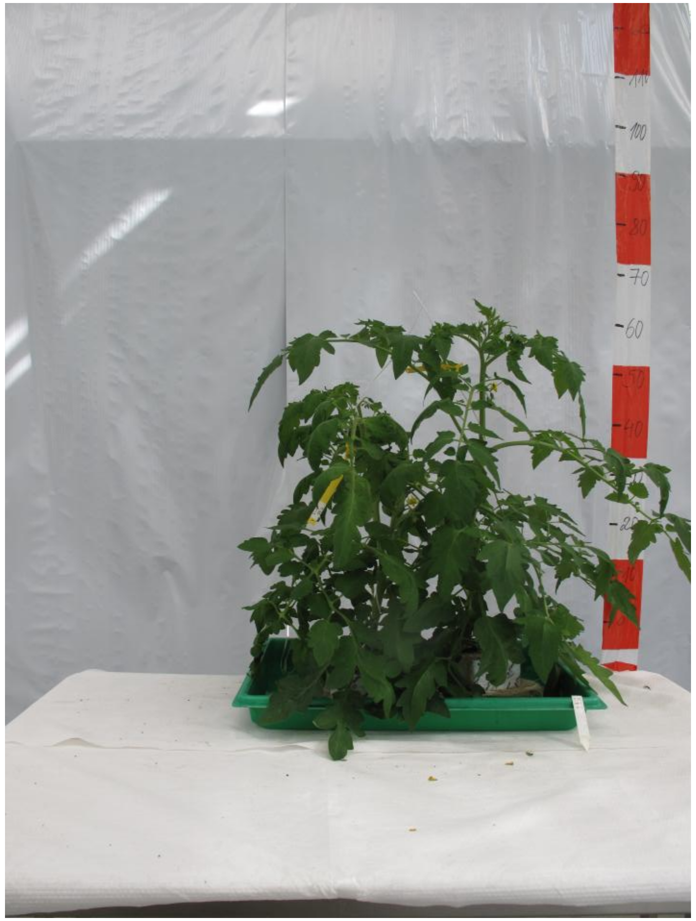

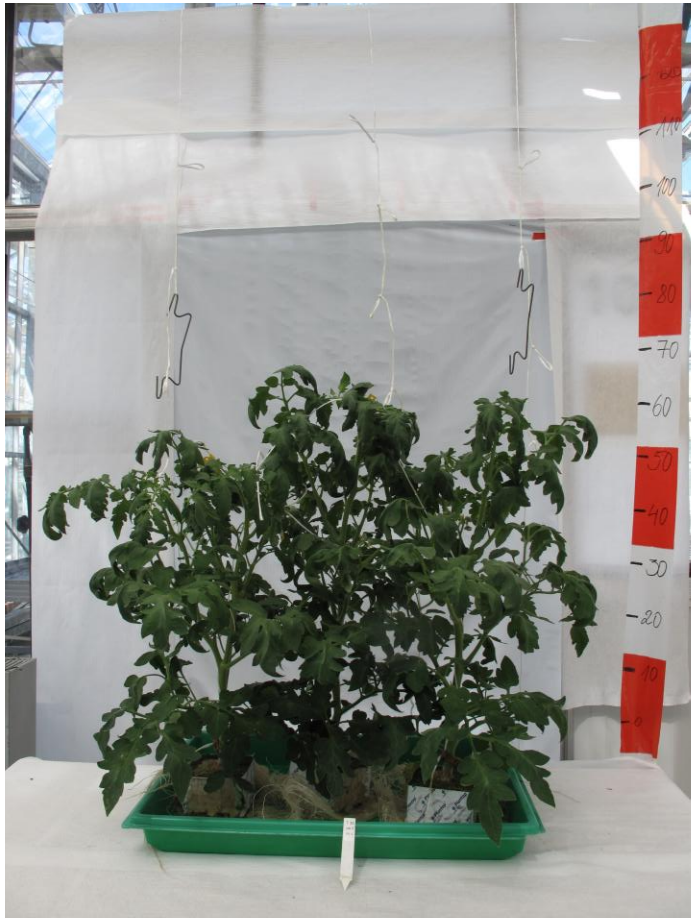

T36

control

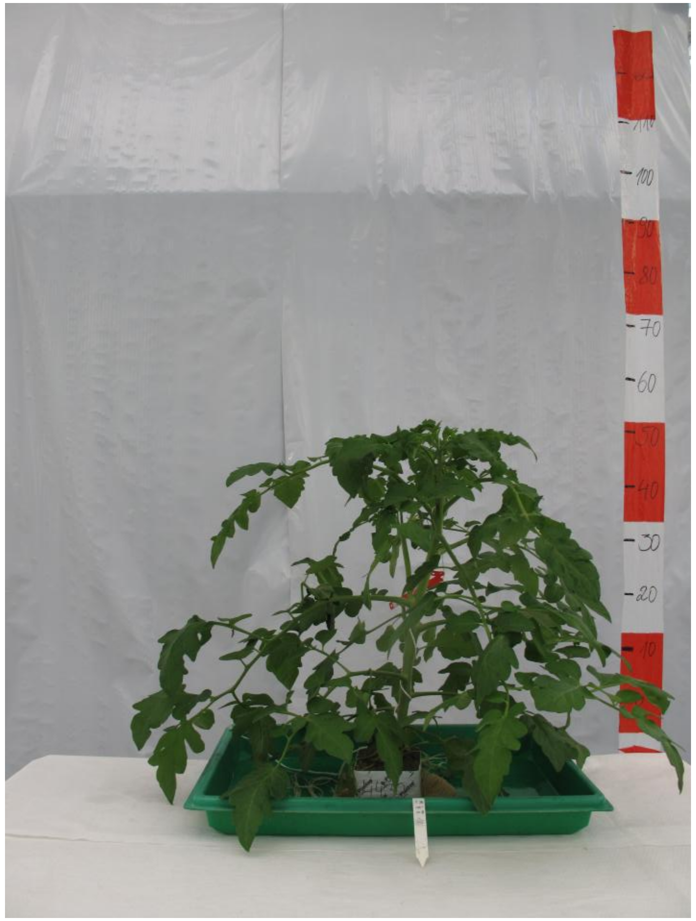

heat

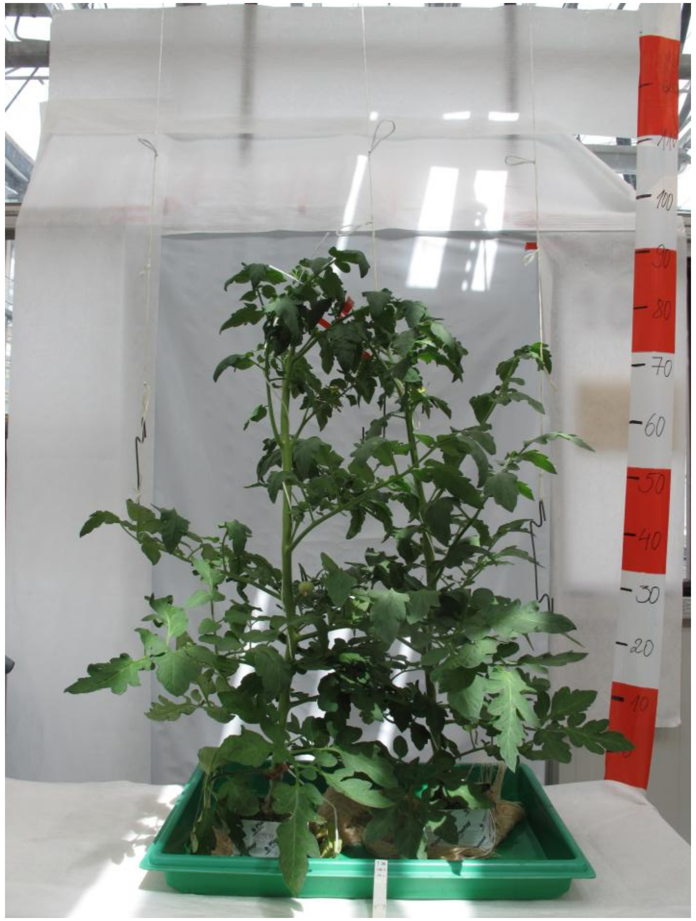

T37

control

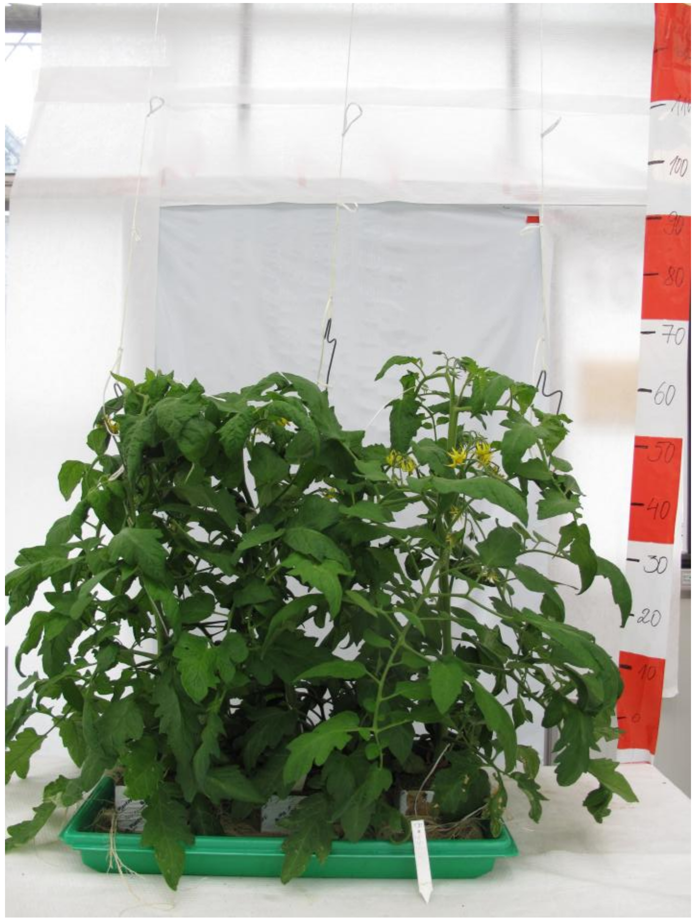

heat

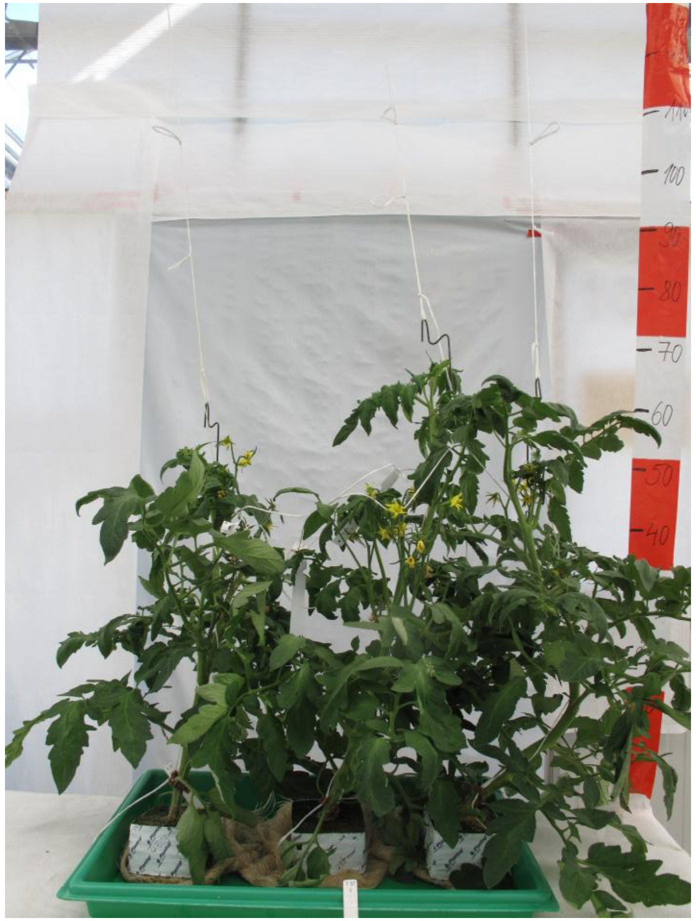

T38

control

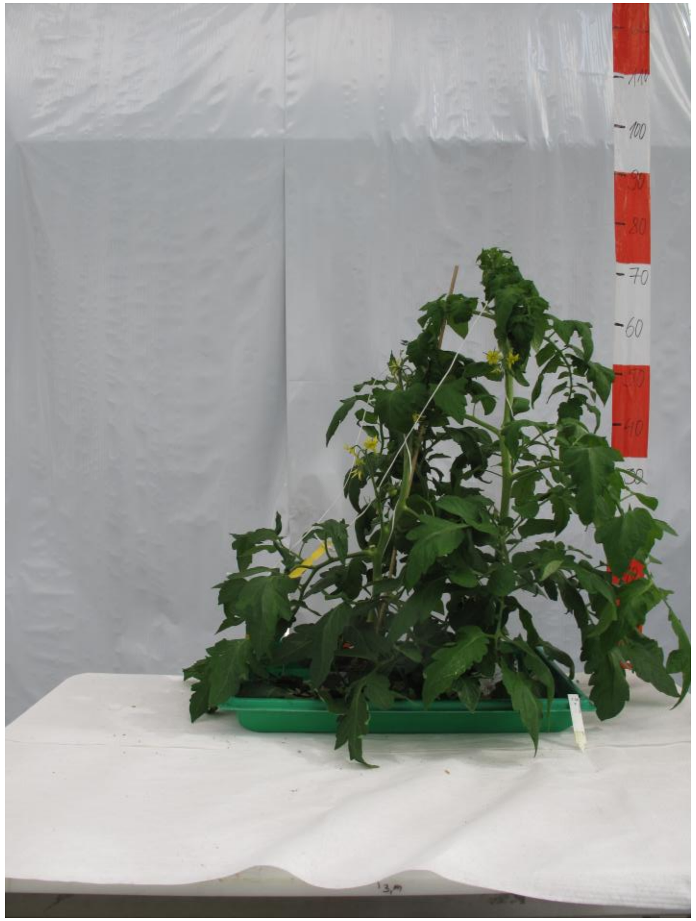

heat

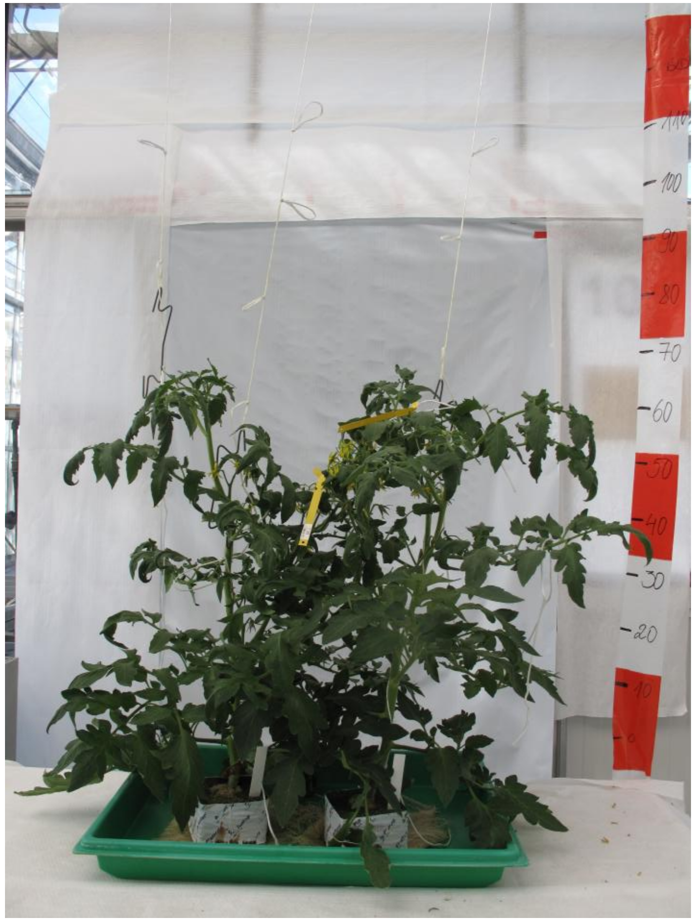

T39

control

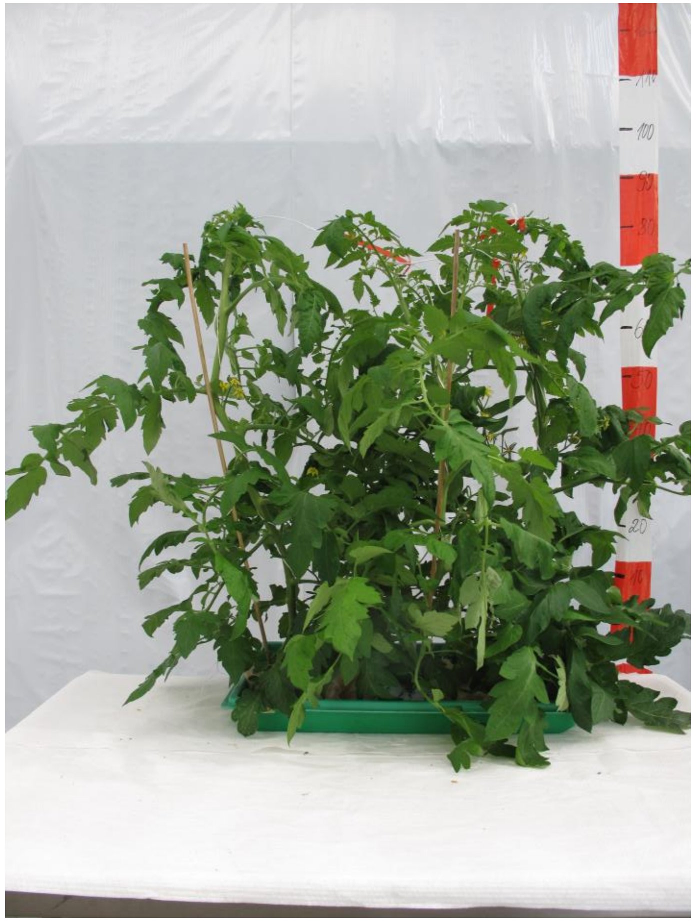

heat

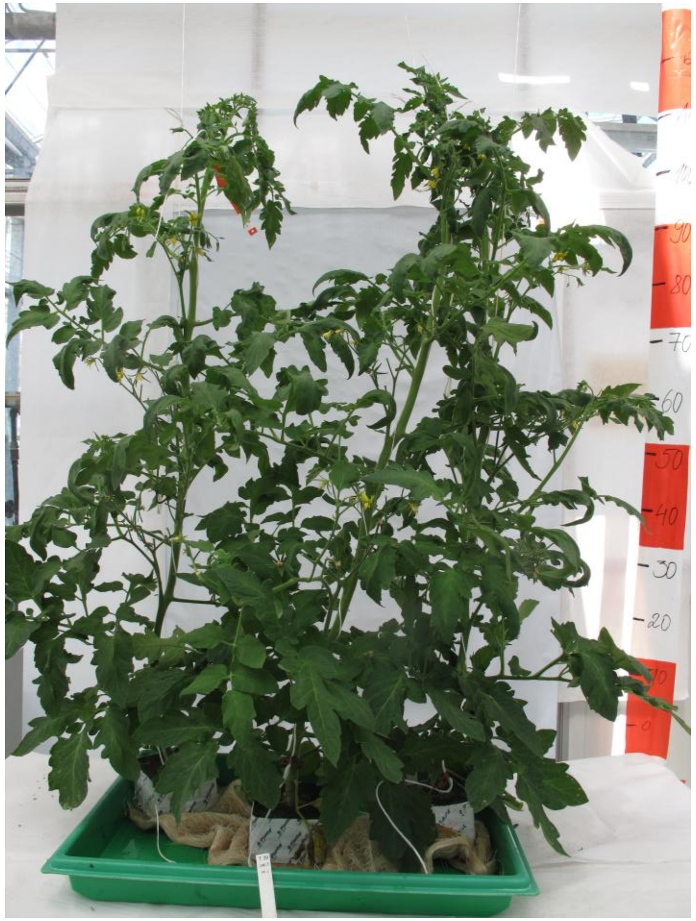

T40

control

- NA

heat

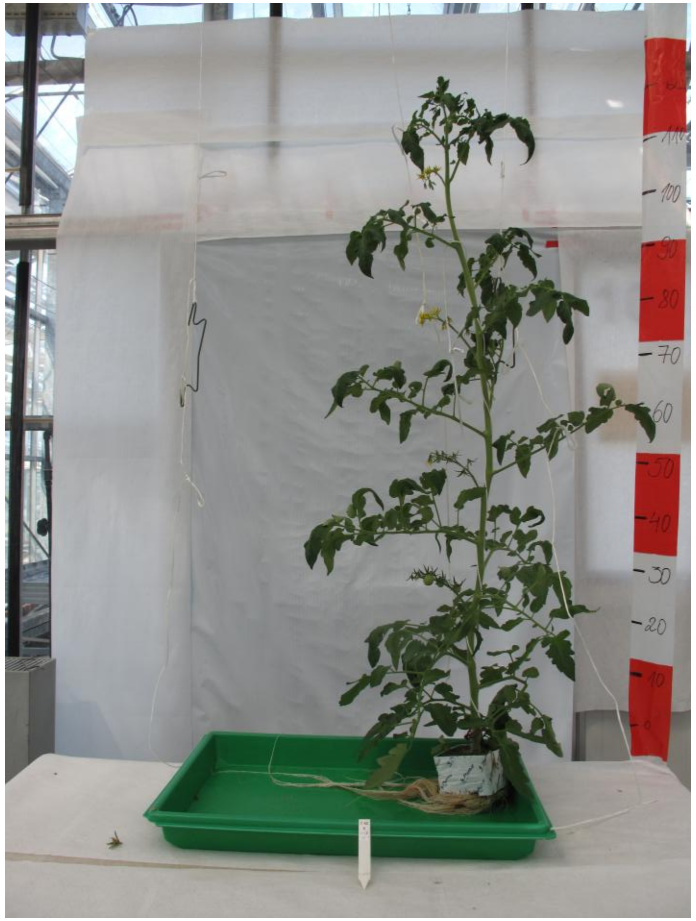

T41

control

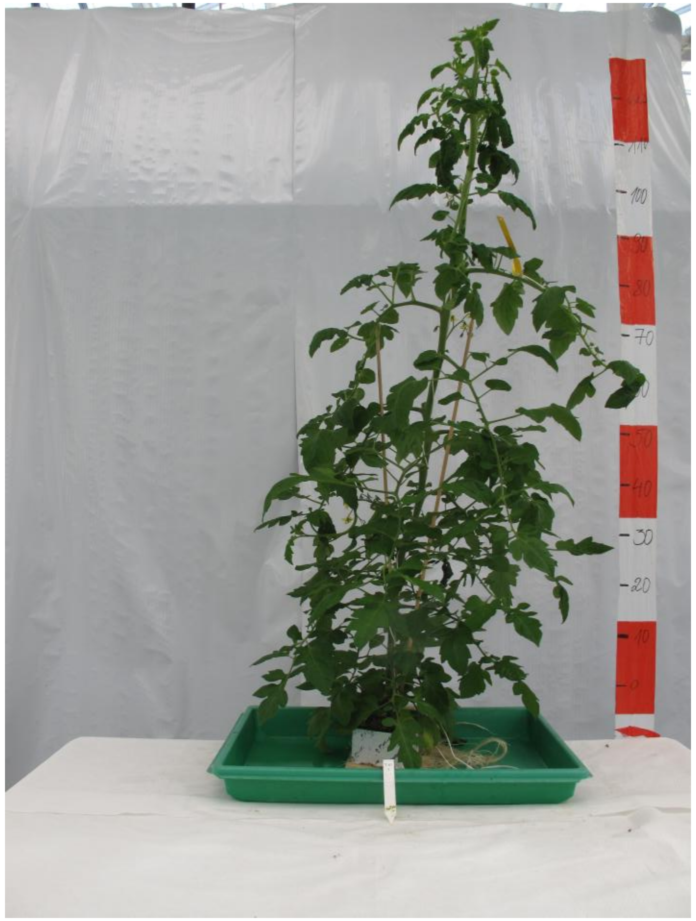

heat

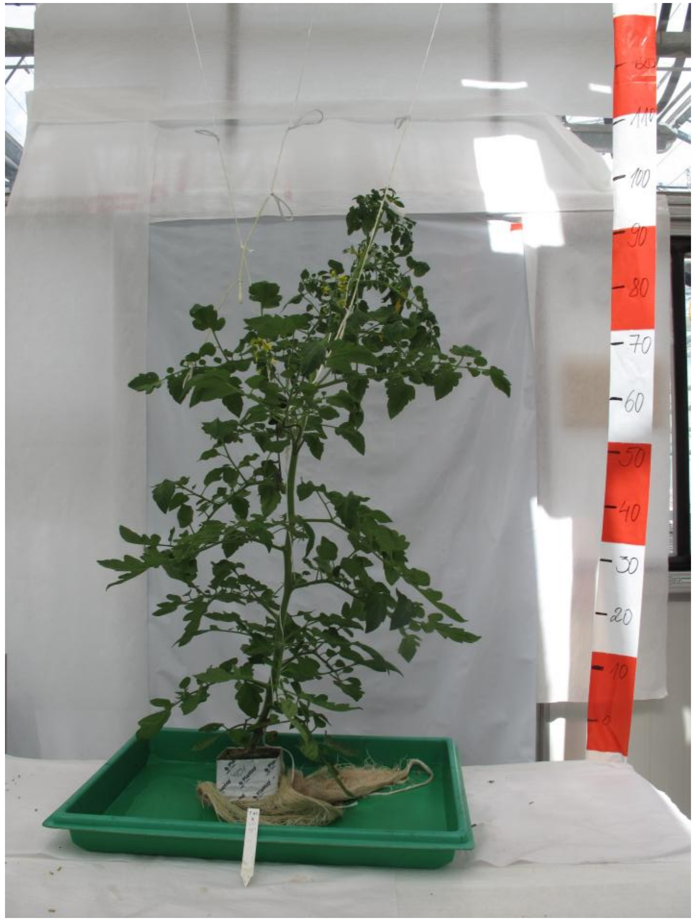

T42

control

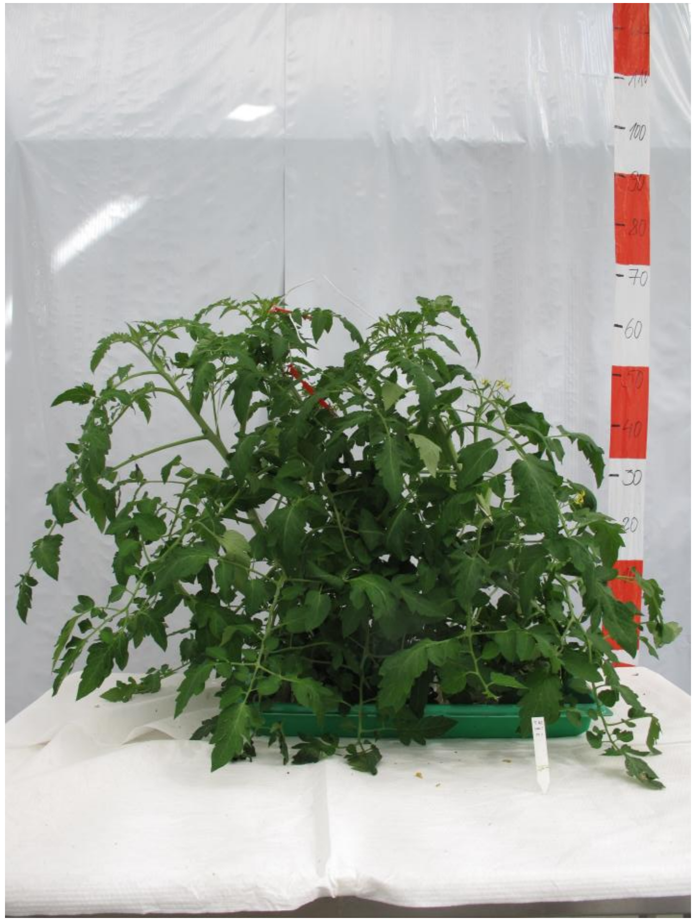

heat

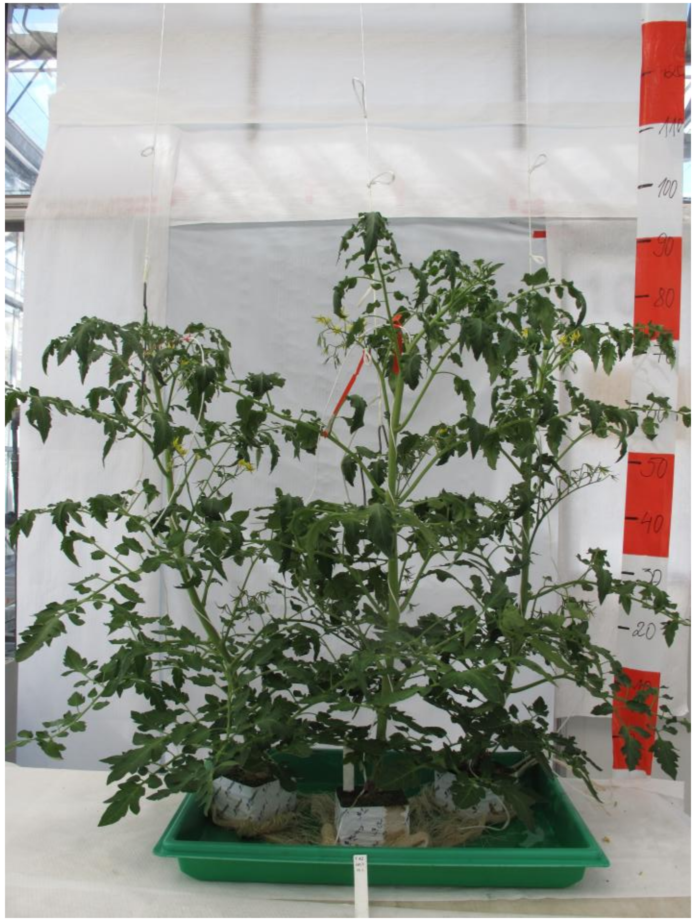

T43

control

heat

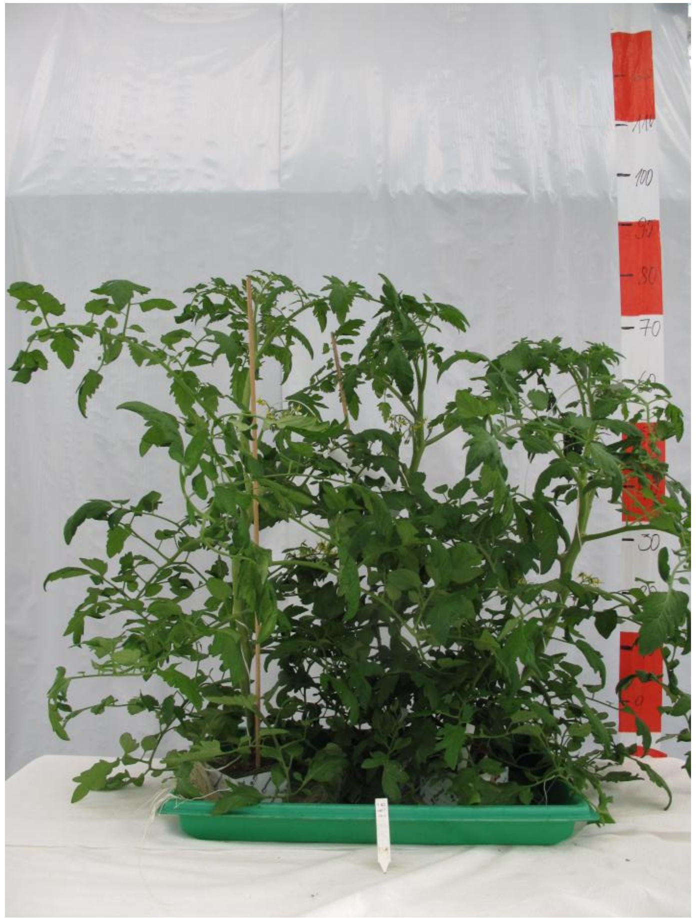

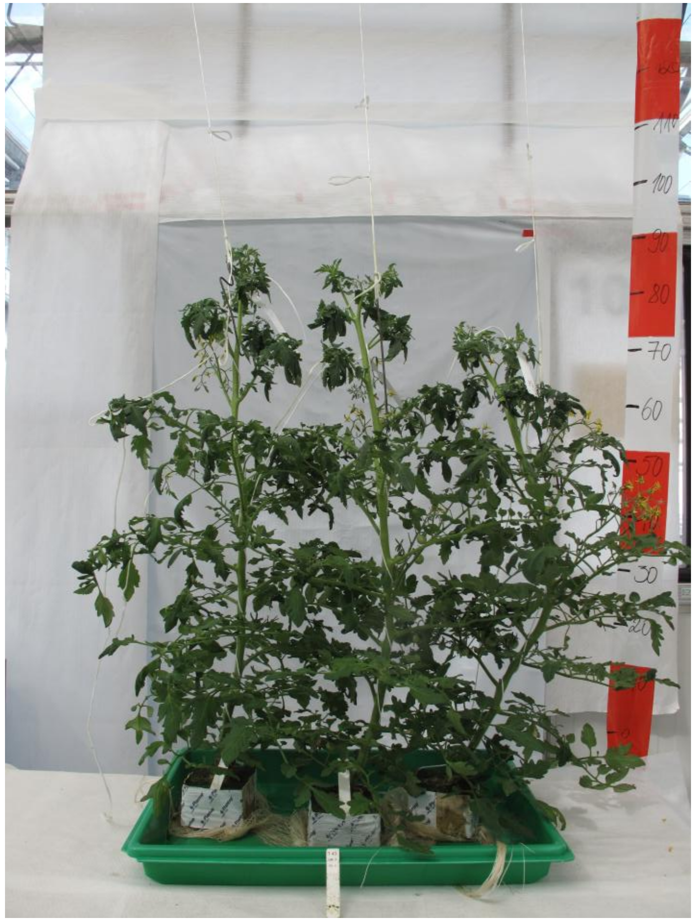

T44

control

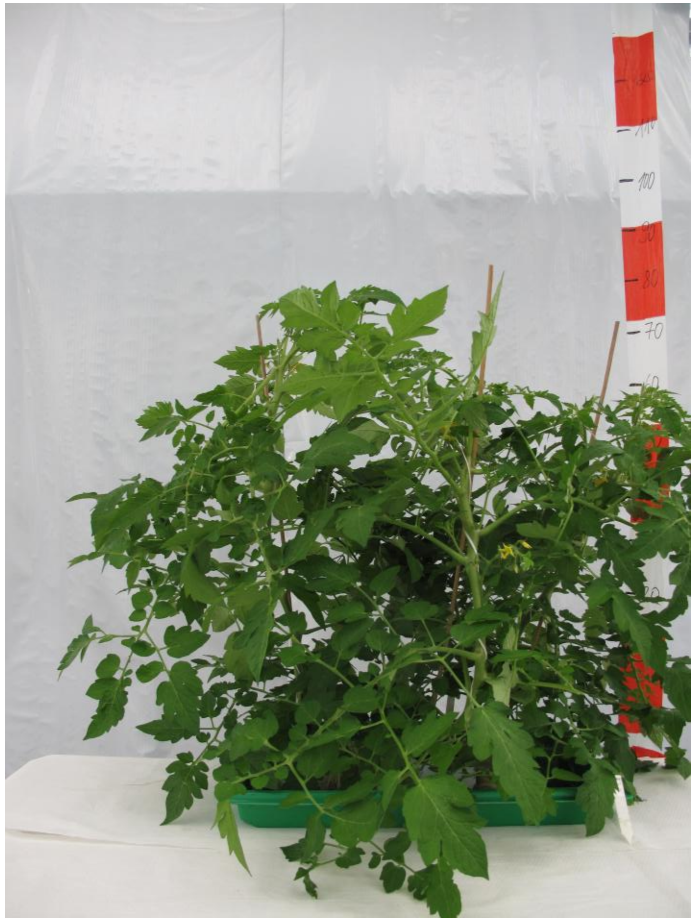

heat

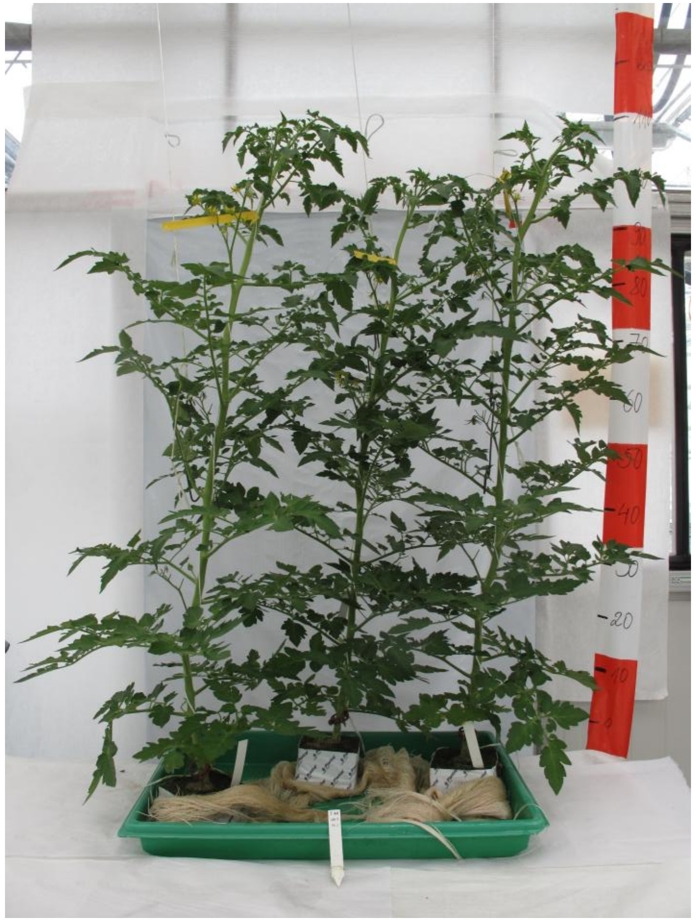

T45

control

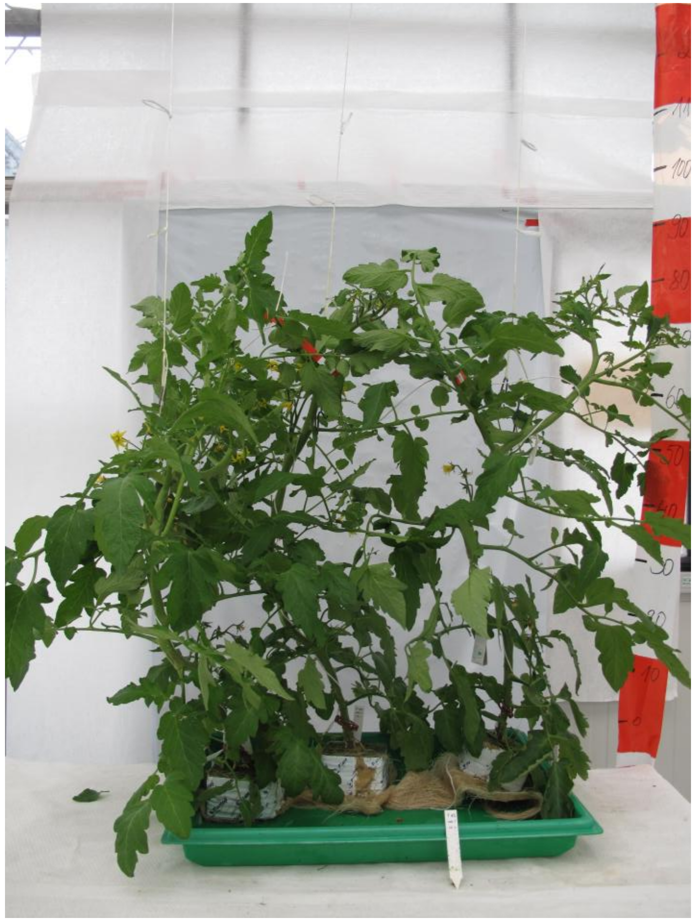

heat

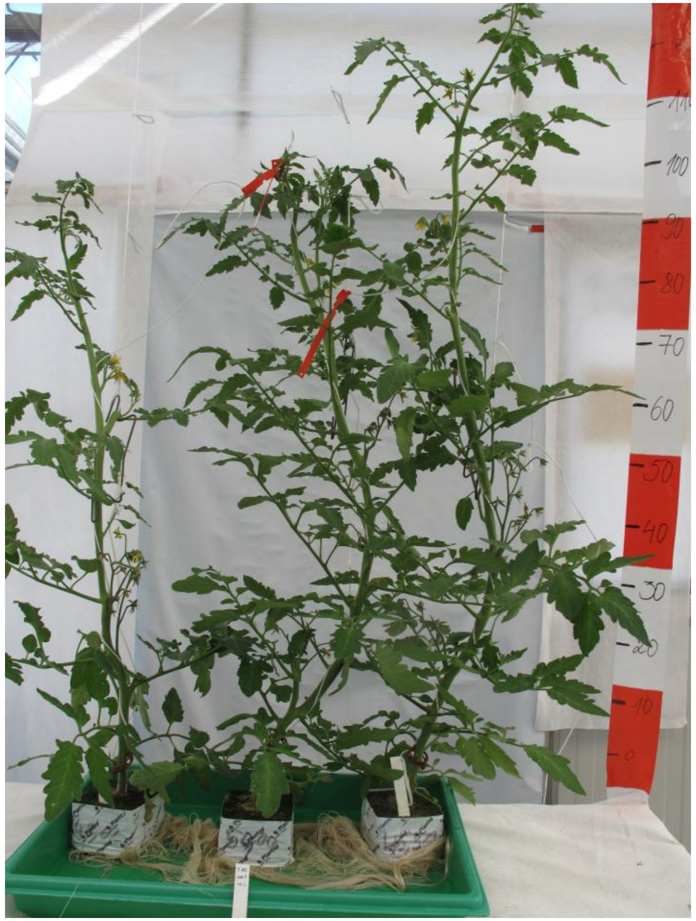

T46

control

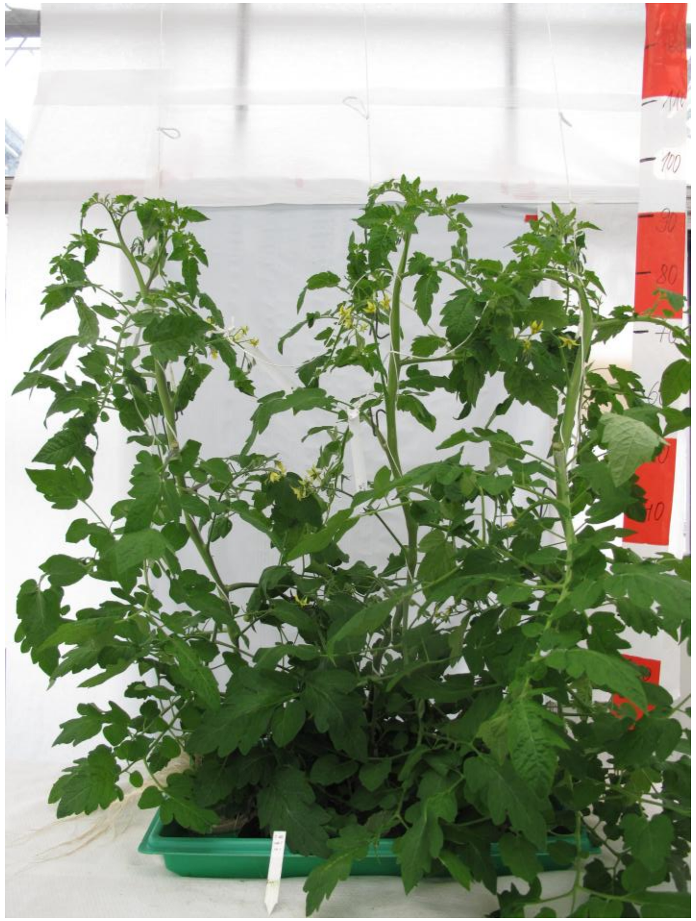

heat

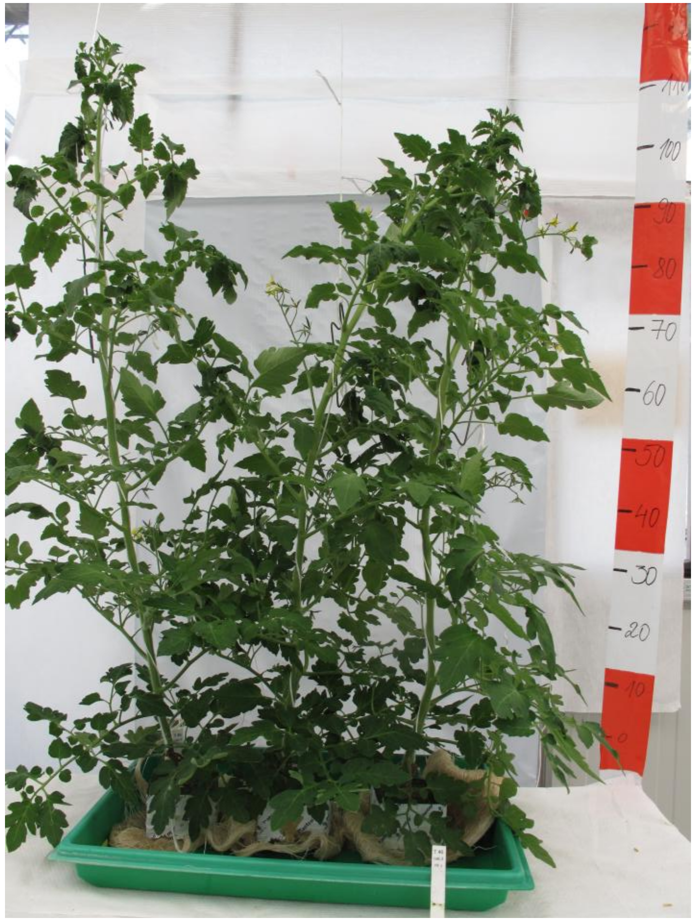

T47

control

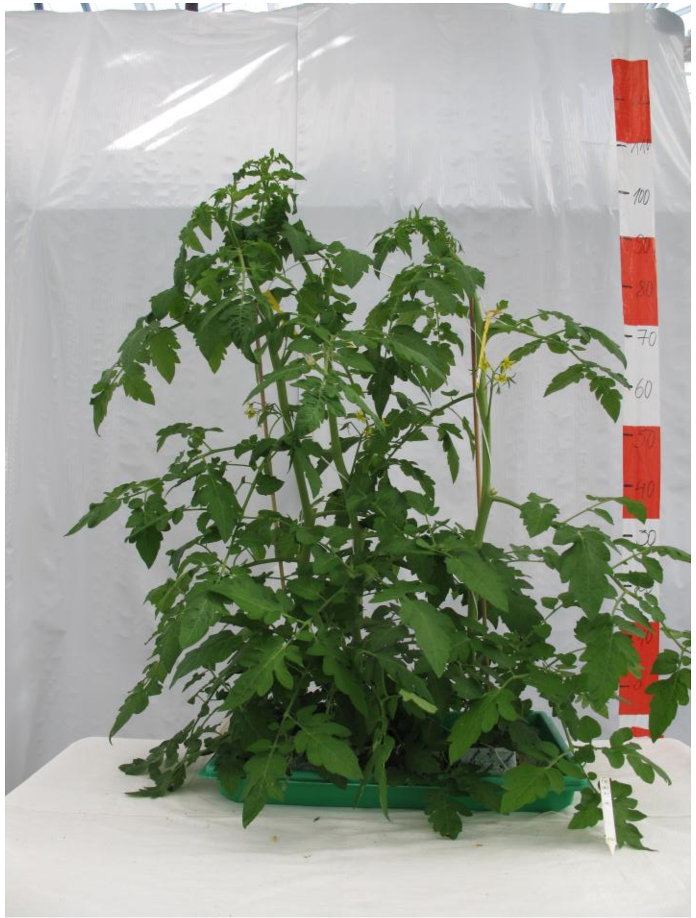

heat

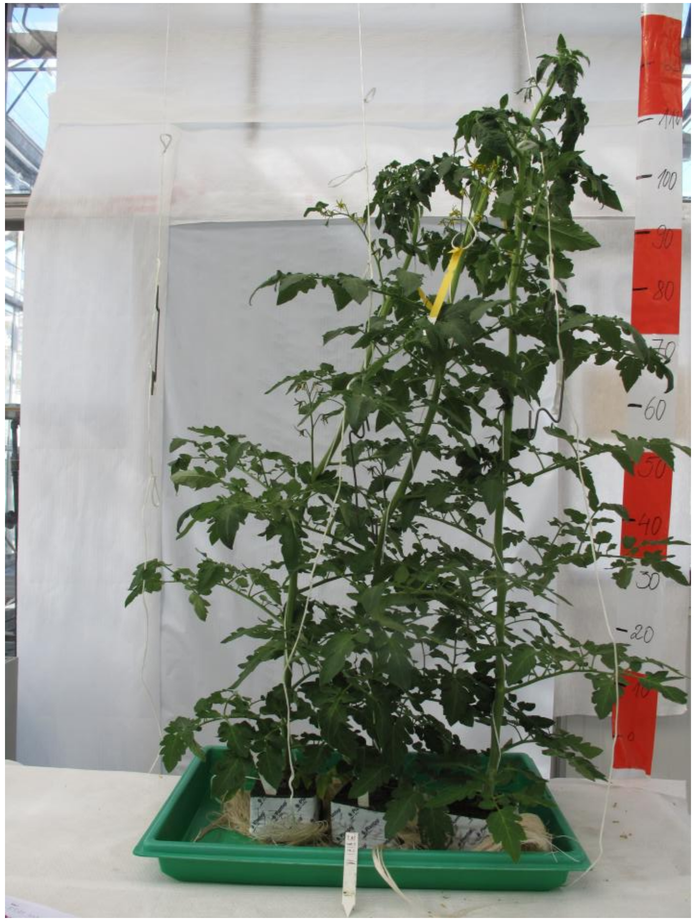

T48

control

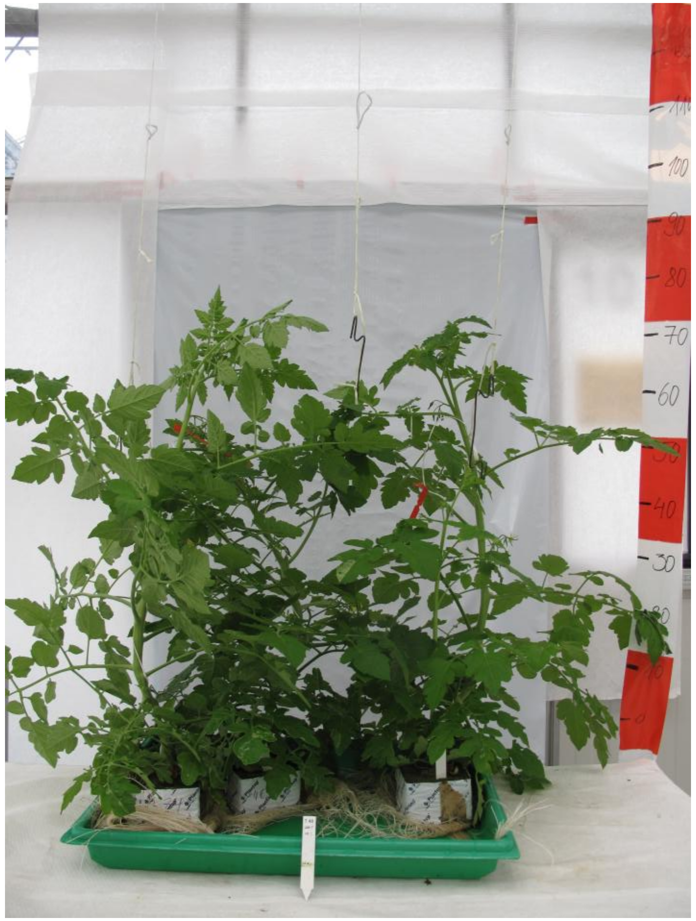

heat

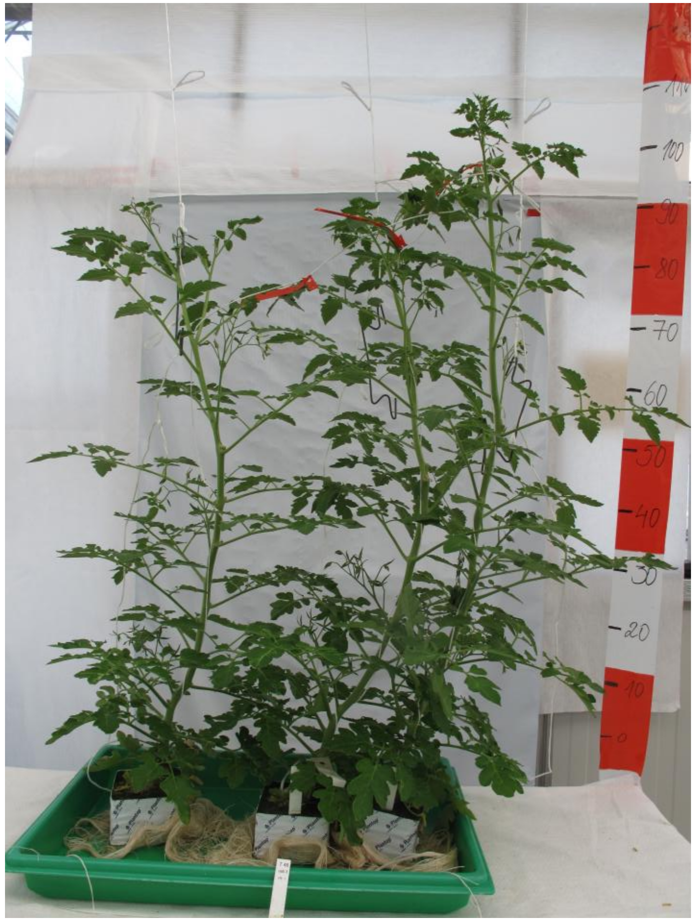

T49

control

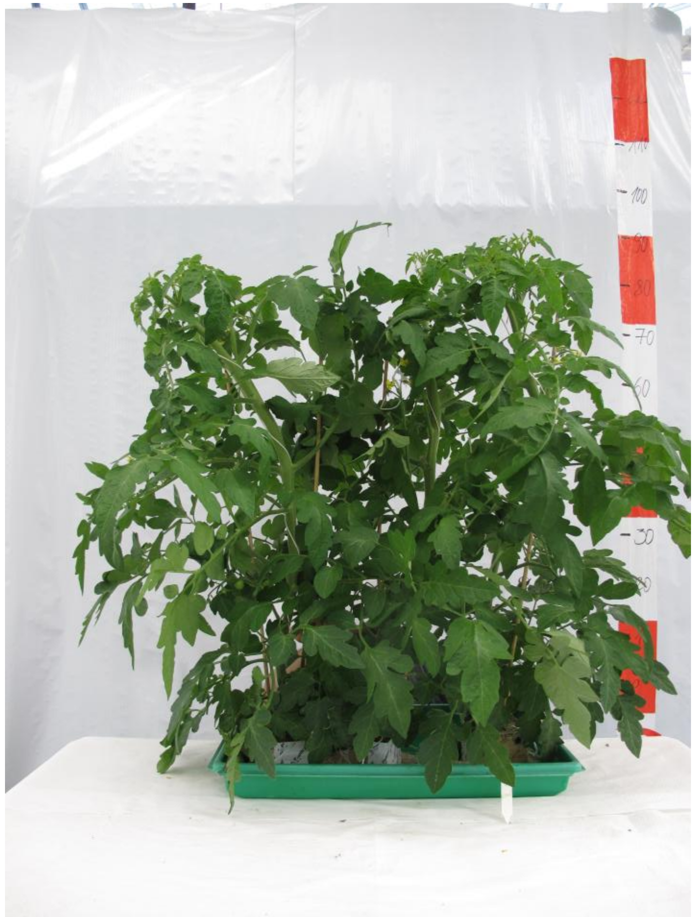

heat

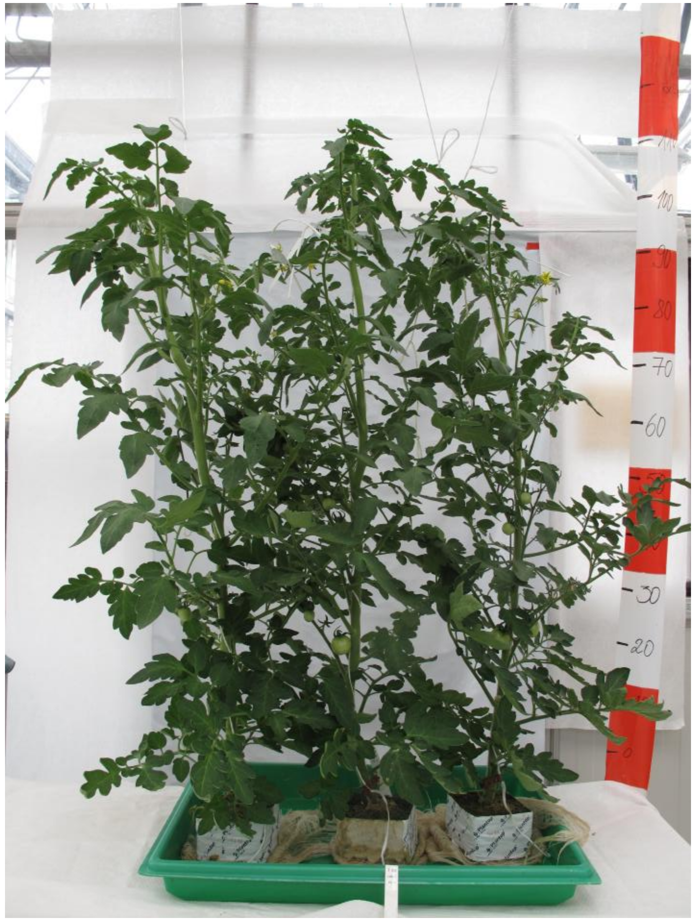

T50

control

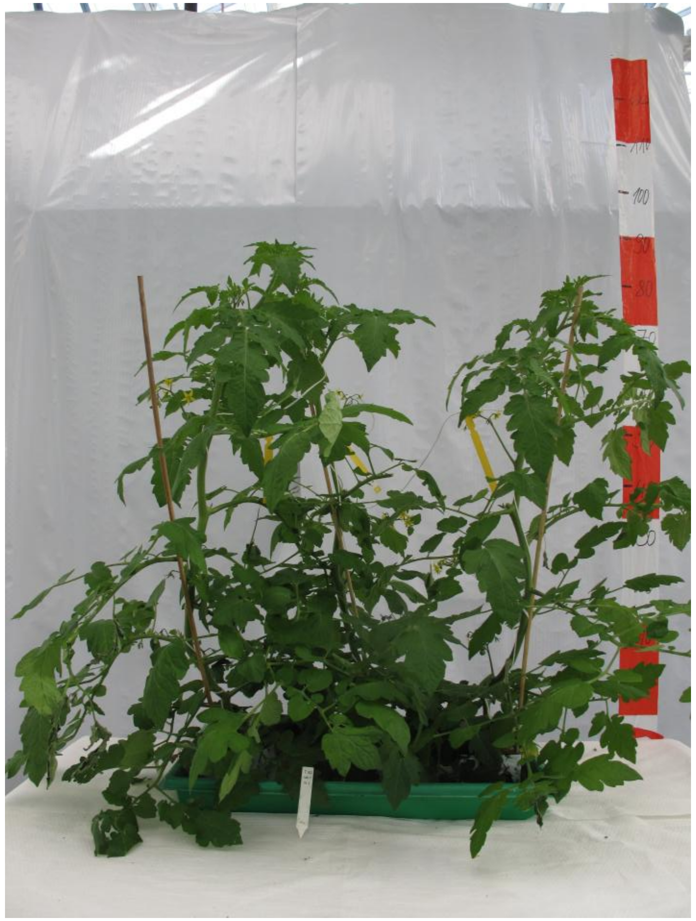

heat

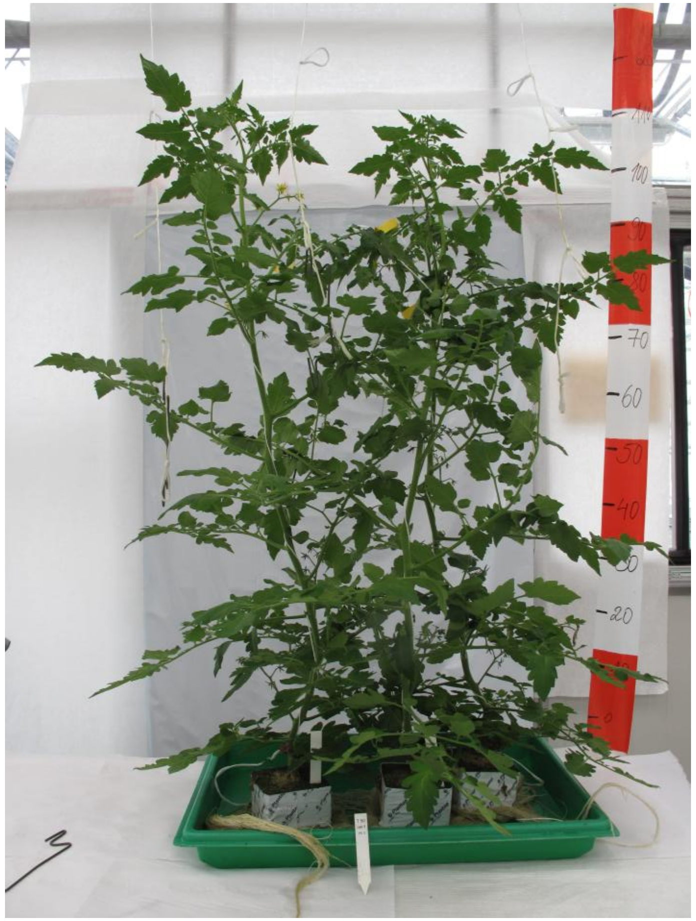

T51

control

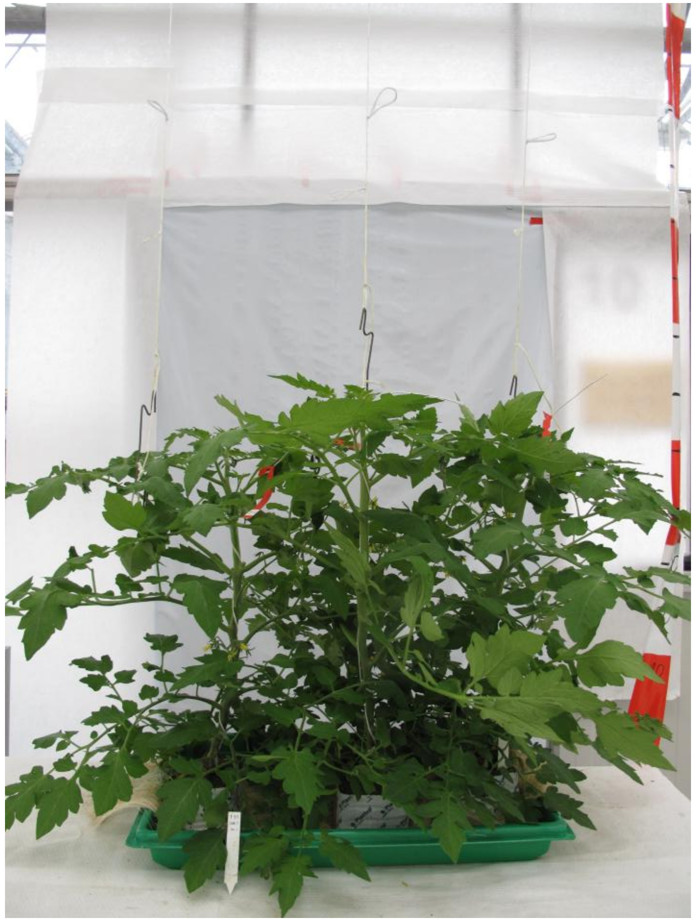

heat

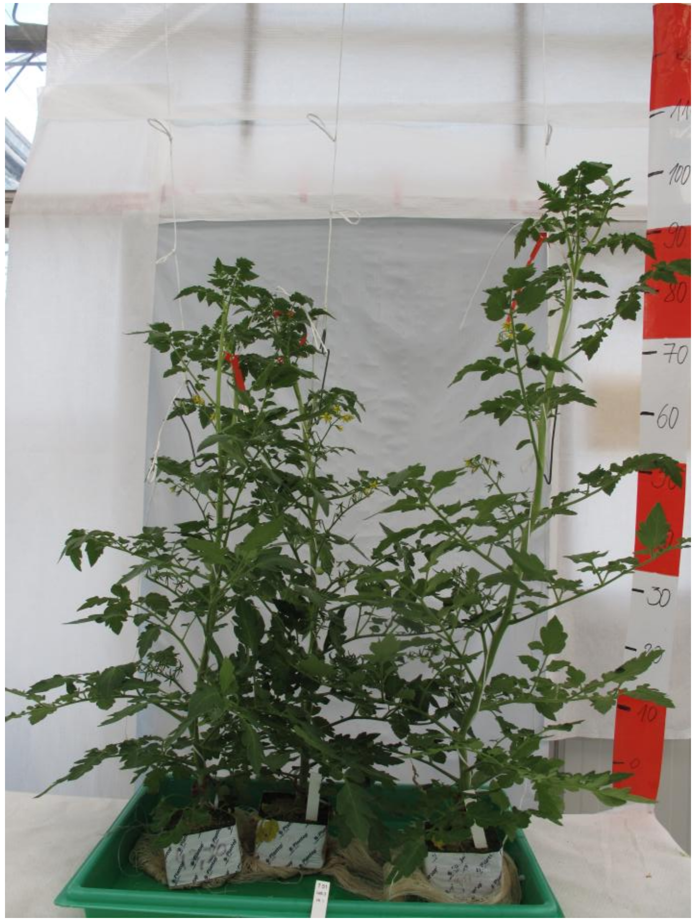

T52

control

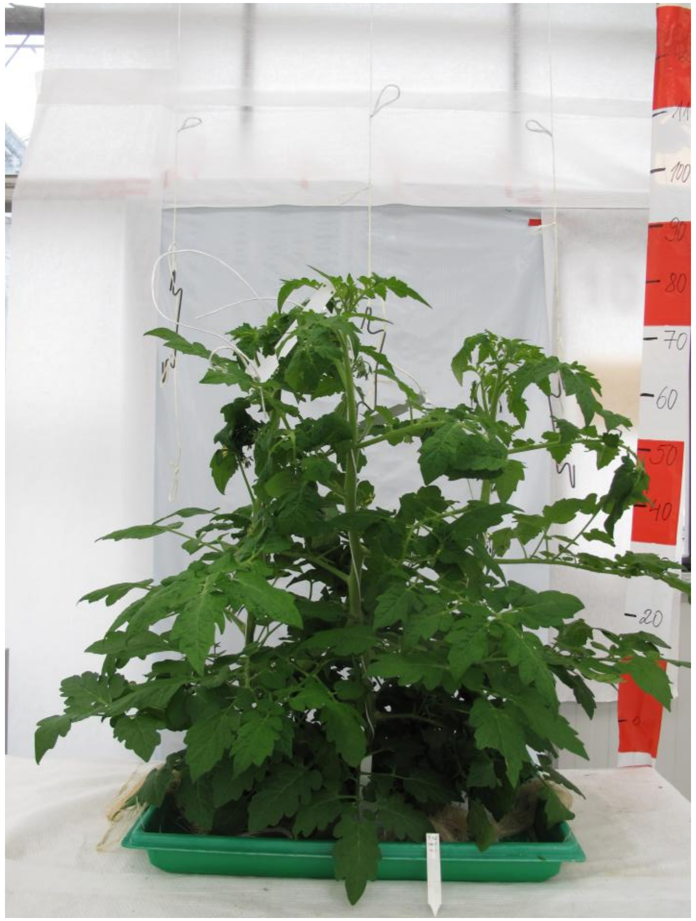

heat

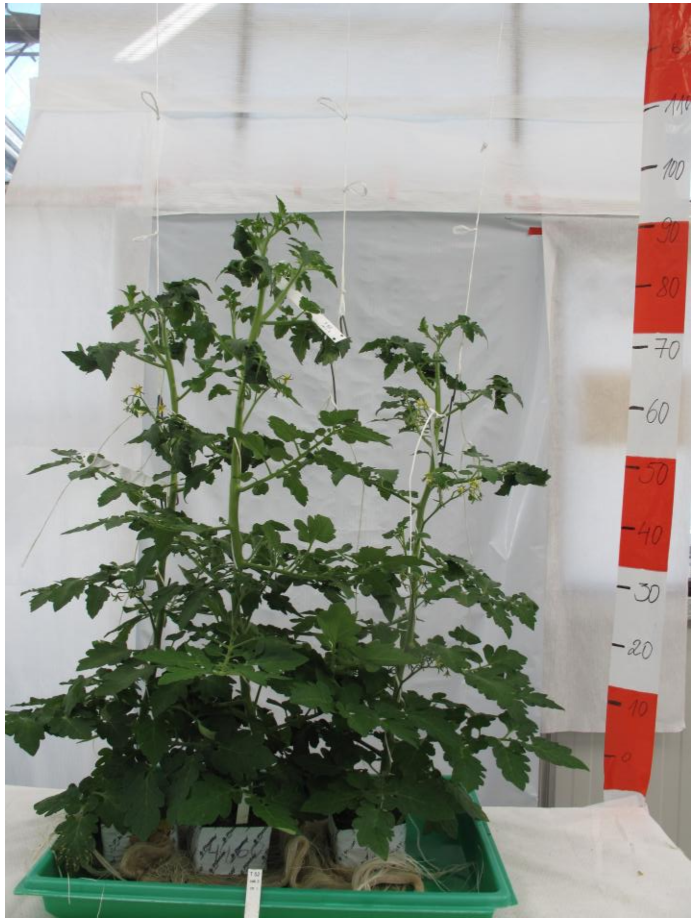

T53

control

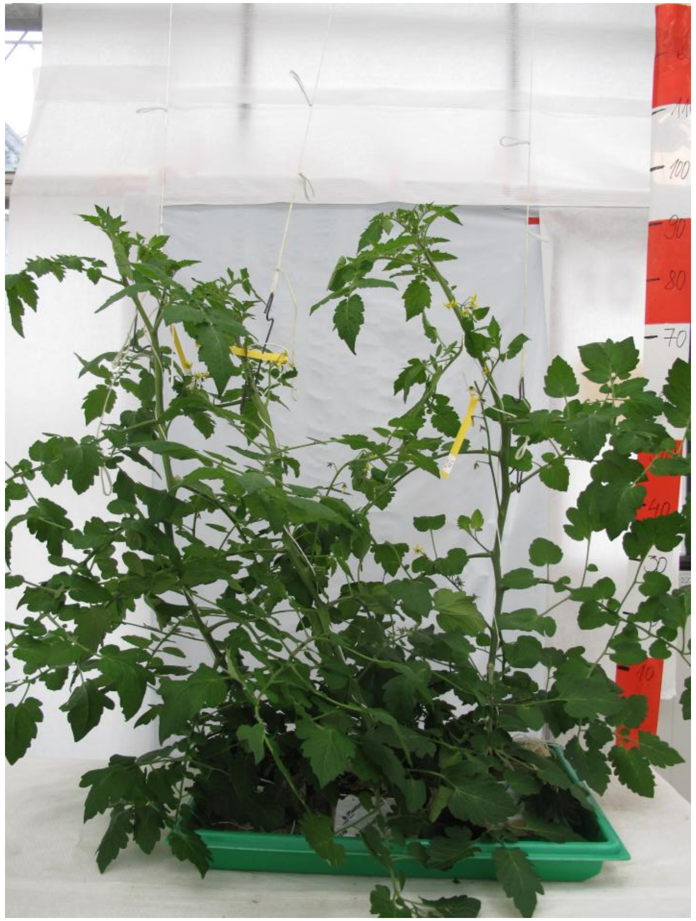

heat

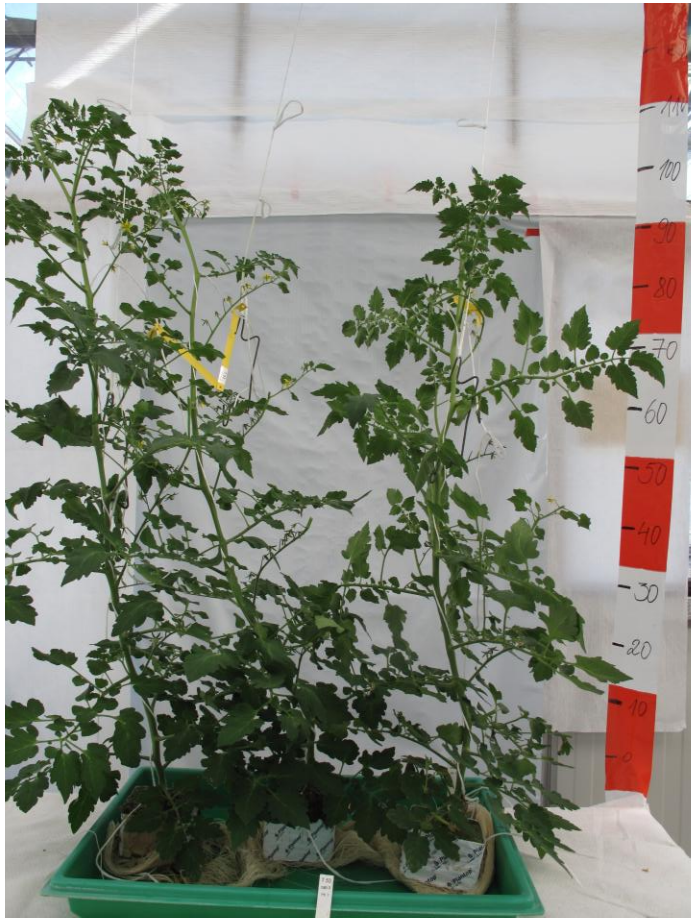

T54

control

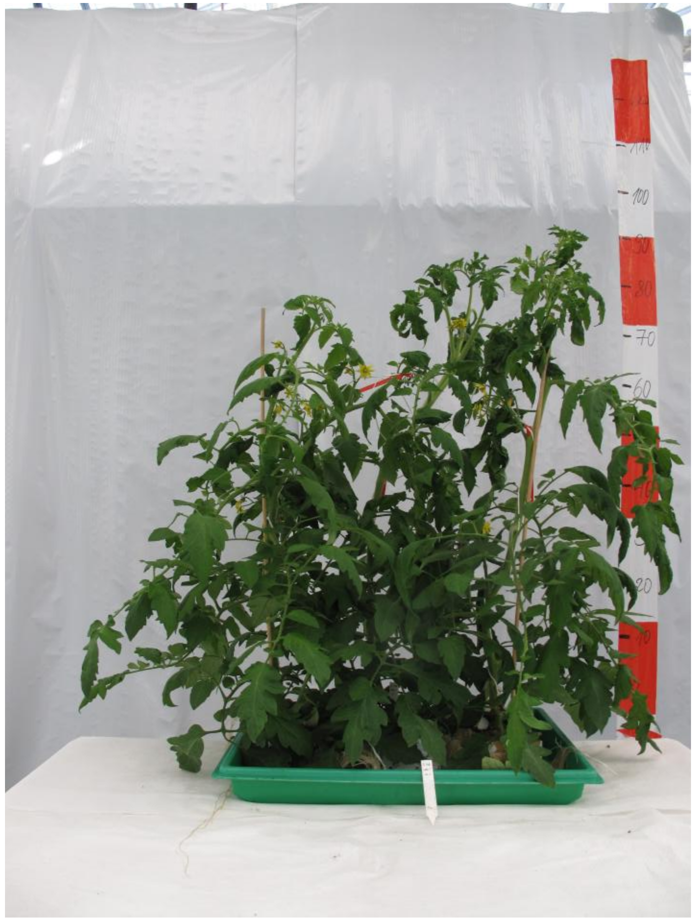

heat

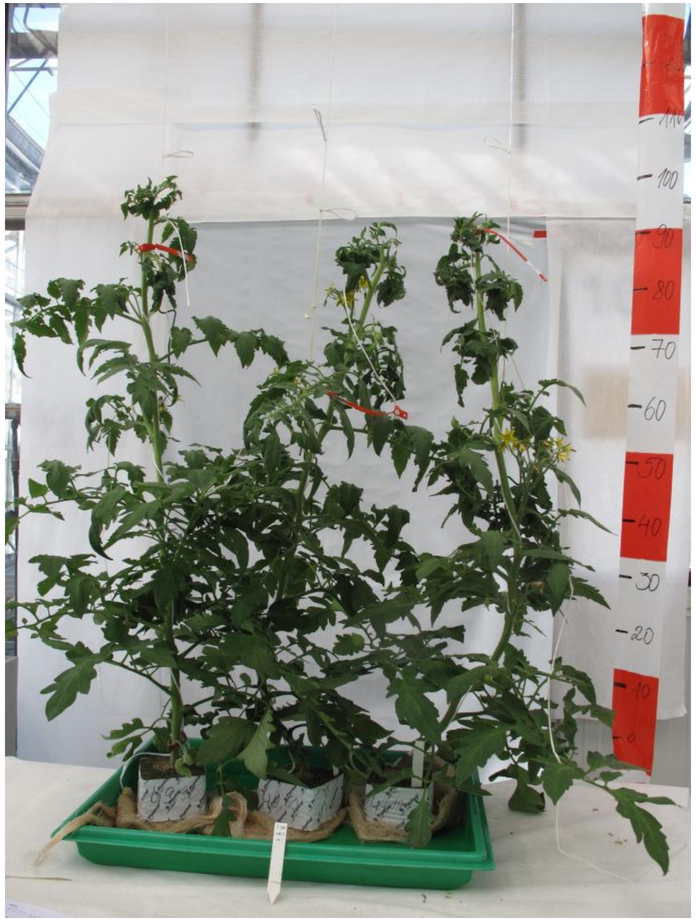

T55

control

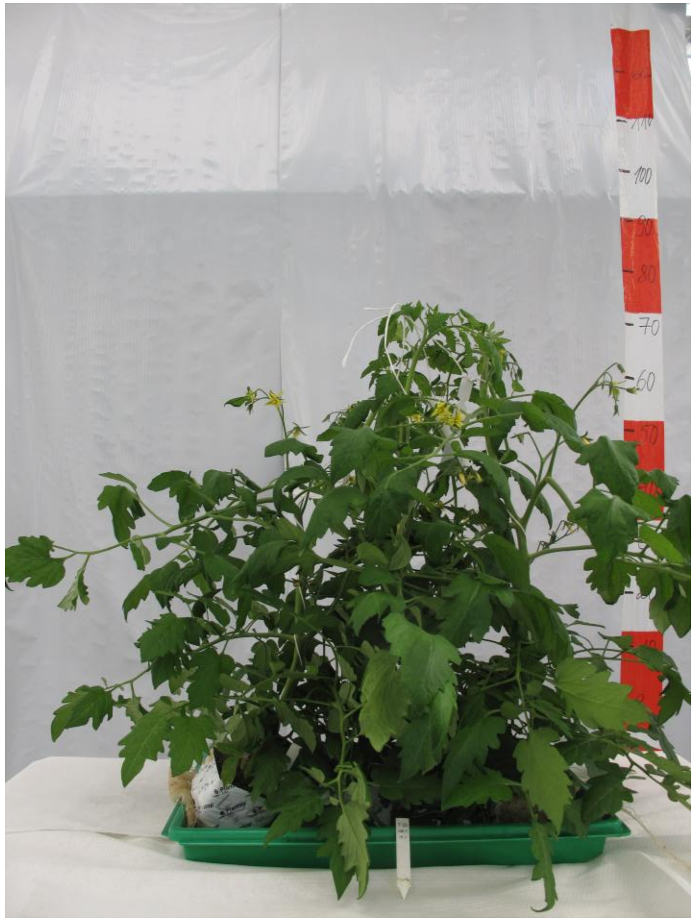

heat

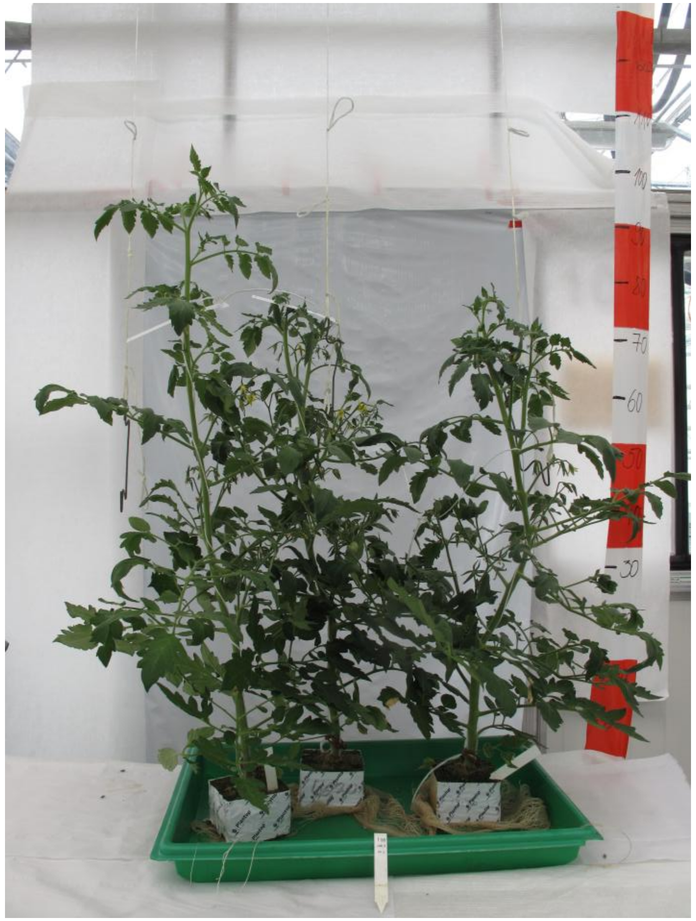

T56

control

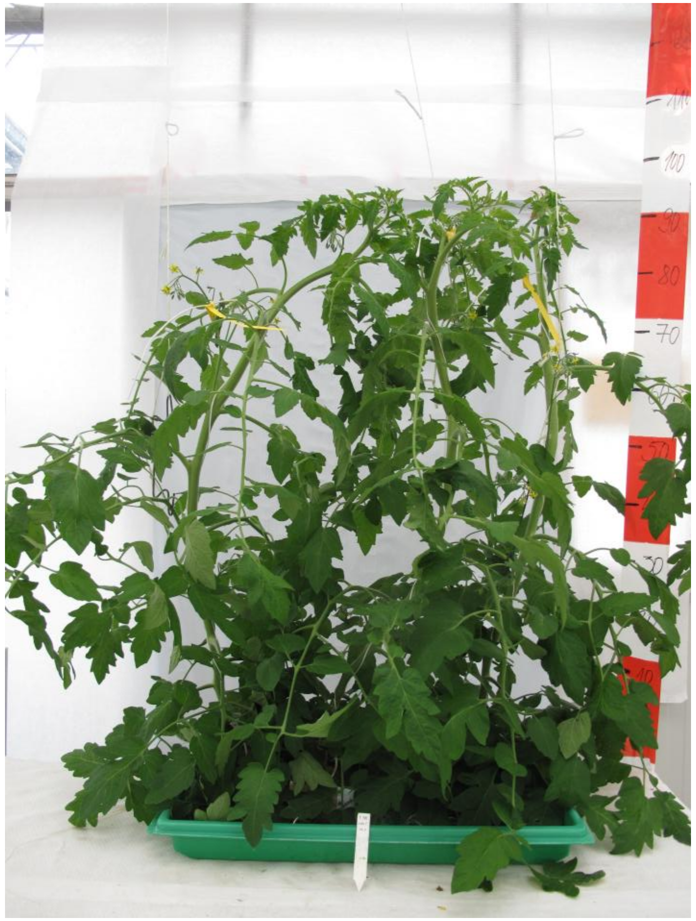

heat

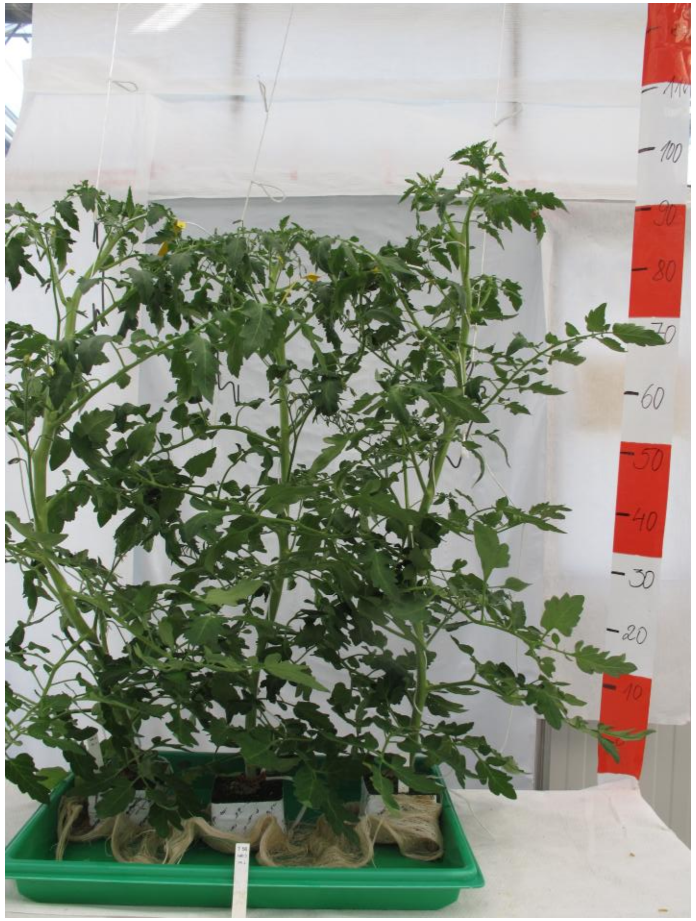

Supplement: Supplementary file 2 [file DataSheet2.pdf]
